# Cereblon-related mild intellectual disability disrupts response inhibition and uniformity of group–individual strategies

**DOI:** 10.3389/fnins.2026.1782687

**Published:** 2026-07-13

**Authors:** Chan Hee Kim, Kyogu Lee, Jeong-Eun Seo, Yoon Jeong Heo, Se-Young Choi

**Affiliations:** 1 Department of Physiology, Dental Research Institute, Seoul National University School of Dentistry, Seoul, Republic of Korea; 2 Department of Intelligence and Information, Artificial Intelligence Institute, Seoul National University, Seoul, Republic of Korea; 3 Department of Musicology, College of Music, Seoul National University, Seoul, Republic of Korea; 4 Department of Korean Music, Seoul National University, Seoul, Republic of Korea

**Keywords:** auditory duration discrimination, behavioral strategy, group-level and individual-level responses, intellectual disability, reaction time and latency, response inhibition and impulsivity

## Abstract

Temporal processing, including duration discrimination, is essential for survival and communication across species. Intellectual disability (ID), which can arise from diverse causes, including mutation in Cereblon (CRBN) gene, impairs duration discrimination. CRBN-related ID is associated with abnormal cognitive behaviors and may disrupt both perceptual and behavioral processes involved in duration discrimination. However, cross-species behavioral strategies, their variation with ID, and the behavioral indices underlying these strategies remain unclear. Here, humans and wild-type (WT) mice with typical intelligence and CRBN knockout (KO) mice with ID, all females across species, performed an auditory duration discrimination task involving 10-s and 2-s cues. All groups successfully distinguished the 10-s and 2-s cues, but their latency-based behavioral strategies diverged. In humans and WT mice, reaction time and latency for both stimuli clustered between 2 and 5 s, whereas in KO mice, they varied depending on the stimulus duration. WT mice and humans consistently delayed their responses by approximately 2 s relative to the 2-s cue length, reflecting response inhibition and alignment between group-level and individual-level responses. In contrast, KO mice exhibited more variable response patterns, consistent with impulsivity and misalignment between group-level and individual-level responses. These findings suggest that CRBN-related ID preserves duration discrimination while altering the behavioral response pattern associated with typical intelligence.

## Introduction

Duration is a fundamental element for interpreting external stimuli and enabling communication. Auditory signals are organized into discrete units, such as phonemes or tones, whose durations convey meaning in contexts ranging from animal vocalizations to human speech and music. Humans can discriminate duration (Creelman, 1962), and similar abilities have been demonstrated in animals (Klink and Klump, 2004; Penney et al., 2008; Van Rooyen et al., 2008; Li et al., 2021). Although they do not share the same language, they exhibit comparable behavioral responses in tasks requiring discrimination between tones of different durations (Hellstrom and Almkvist, 1997). Sensory discrimination is closely linked to intelligence (Melnick et al., 2013); even mild intellectual disability (ID; formerly mental retardation) can impair fundamental cognitive abilities (Glascher et al., 2009; Chen et al., 2017), including duration discrimination, leading to delayed or less accurate responses compared with individuals of typical intelligence (DeFord, 1996; Rattat and Collie, 2020; Gourlat et al., 2024). ID is characterized by significant deficits in intellectual functioning and adaptive behavior across conceptual, social, and practical domains (Schalock et al., 2021). This study investigated whether humans and animals exhibit comparable behavioral strategies in an auditory duration discrimination task and whether these strategies are influenced by intelligence.

Behavior during task performance often depends on cognitive strategies (Harris and Pressley, 1991) that optimize learning, problem solving, and performing. Behavioral variability may reflect differences in these strategic approaches (Whitely, 1978; Hertwig and Gigerenzer, 2011). Reaction time (RT), defined as response latency, and correct rate (CR), reflecting response accuracy, are fundamental behavioral measures in both humans and animals. RT is particularly informative for evaluating cognitive strategies (Hockley, 1984; Chen et al., 2017; De Boeck and Jeon, 2019) and may correlated with CR while being influenced by both subject- and stimulus-related factors. In mice, RT differs methodologically from human RT due to differences in the apparatus, protocol, and the repetitive nature of behavioral responses. Building on our previous work (Kim, 2025), which defined and categorized RT-related indices derived from mouse responses to conditioned stimuli (CSs) of varying durations, we selected 10-s and 2-s white noises for the present duration discrimination task. This contrast was chosen to maximize perceptual contrast and to elicit distinct RT patterns. We evaluated the extent to which RT-related indices captured characteristics specific to the subjects and how they associated with CR (Supplementary Figure 1). RTs to 10-s and 2-s conditions may reveal differences in response patterns across species and levels of cognitive performance. RT has been associated with several behavioral processes, including impulsivity (de Wit, 2009), response inhibition (Burle et al., 2004), and behavioral strategies involved in decision making (Wierda et al., 2025). Moreover, the peak distributions of head entry timing in mice reveal the RT patterns that resemble those observed in humans (Kim, 2025). These RT-based behavioral indices would therefore be useful for characterizing heterogeneous behavioral phenotypes associated with intellectual disability.

Transgenic animal models with targeted gene suppression have been instrumental in overcoming the limitations of patient-based research and in elucidating the neural mechanisms underlying ID (Verma et al., 2019). In the present study, we generated an ID model via mutations in the Cereblon (CRBN) gene, which cause mild ID (Lee et al., 2010; Sawamura et al., 2015), the most common form of ID (Patel et al., 2020). CRBN has been implicated in cognitive processes and memory function (Higgins et al., 2010; Song et al., 2018), and its dysfunction has been associated with atypical cognitive functions (Rajadhyaksha et al., 2012; Bavley et al., 2018; Choi et al., 2018) and autism spectrum disorder (ASD) (Sato et al., 2021). Individuals with ASD have been reported to show reduced capacity to differentiate time intervals (Falter et al., 2012); however, it remains unclear whether these impairments reflect a domain-general deficit in time perception or arise from more specific cognitive or sensory mechanisms (Casassus et al., 2019). These findings suggest that CRBN deficiency may affect both perceptual and behavioral performance in duration discrimination tasks. However, whether duration discrimination is preserved in individuals with CRBN-related ID and how it differs from that of individuals with typical intelligence, remains poorly understood. Because ID is highly heterogeneous (Kaufman et al., 2010) and CRBN mutations cause autosomal recessive nonsyndromic ID (Bavley et al., 2018), isolating CRBN-specific phenotypes in humans is challenging. CRBN knockout (KO) mice therefore provide a valuable model for investigating behavioral characteristics specific to this gene, and integrating human and animal findings may contribute to improved diagnostic and educational approaches.

**Figure 1:**
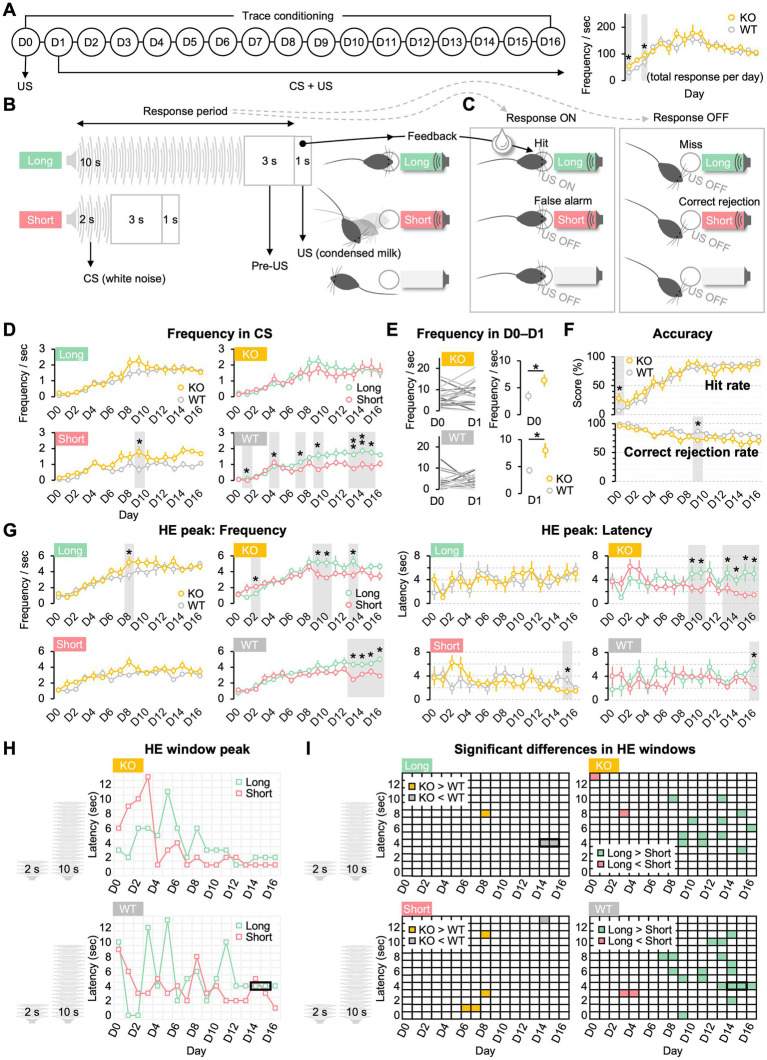
Mouse experiment and behavioral responses. **(A)** Trace conditioning over 16 days showed no difference in total HEs per day between KO and WT mice. **(B)** Mice were trained on a duration discrimination task involving 10-s (Go) and 2-s (No-Go) conditions. **(C)** In the 10-s condition, US delivery followed an HE between CS onset and pre-US offset. A hit was defined as an HE occurring within 10 s of CS onset. **(D)** Significant group and condition differences in HE frequency appeared only during the CS period in late training days. **(E)** On D0–D1, KO mice had higher HE frequency than WT. **(F)** Accuracy (hits and correct rejections) during CS did not prominently differ across groups or conditions. **(G)** HE peak frequency varied by condition. HE peak latency was stable in WT mice but variable in KO mice. **(H)** In late training, HE window peak latency occurred between 1 and 3 s across conditions in KO mice, whereas WT mice peaked after 2 s. **(I)** On D14–D15, the 4–5 s window showed group differences in the 10-s condition and condition differences (10-s vs. 2-s) in WT mice (Supplementary Figure 7). This interval overlapped with the HE window peak (**H**; Supplementary Figure 6), suggesting a critical decision period for the 10-s condition. See Supplementary Figures 2–7 and Supplementary Tables 1–5 for statistics. Statistical tests: Wilcoxon signed-rank, Mann–Whitney U; *p < 0.05, **p < 0.01 (*N* = 9). Error bars: 95% CI. D, day; CS, conditioned stimulus; US, unconditioned stimulus; Long, 10-s condition; Short, 2-s condition; WT, wild-type; KO, CRBN KO; HE, head entry.

The Go/No-Go paradigm is widely used to probe both perception (e.g., discrimination between 10-s and 2-s durations) and behavior (e.g., response inhibition and impulsivity) (Bezdjian et al., 2009; Loos et al., 2010; Berditchevskaia et al., 2016). In the mouse experiment, this paradigm was adapted such that a reward (unconditioned stimulus, US) was delivered only in 10-s (Go) condition, whereas human participants performed a duration discrimination task to account for the tendency of mice to respond even in No-Go trials (see “Methods” and Figure 1). Despite these methodological differences, both paradigms were designed to assess duration evaluation and response inhibition. Three groups were examined: wild-type (WT) mice, CRBN KO mice, and healthy human participants. We investigated behavioral strategies in humans and mice and examined how CRBN-related ID alters perceptual and behavioral responses to auditory cues of different durations. We further evaluated which behavioral indices differentiate perception from behavior and inhibition from impulsivity in both species. We hypothesized that humans would exhibit response patterns similar to those of WT mice, whereas KO mice would display atypical behavioral responses resulting from perceptual impairments. Because CRBN may affect both perception and behavior, its dysfunction was expected to impair duration discrimination, sound–reward associations, and the regulation of response inhibition and impulsivity. Given that auditory duration discrimination is fundamental to survival (Smith and Harper, 2003) and communication (Oxenham, 2018), and is closely linked to intelligence and language (Sturrock et al., 2021), our findings may provide insights into how altered cognitive strategies, particularly those involving impulsivity and inhibition in decision making (Christodoulou et al., 2006), contribute to communication difficulties in CRBN-related ID, especially in social contexts (Fernandez and Garner, 2007) (Supplementary Figure 1).

## Results

Behavioral data were collected to quantify response timing during task performance. In mice, head entries (HEs) into the food cup were variable, whereas humans exhibited a one-to-one response pattern (see Methods). Behavioral indices, including accuracy and latency, were used for both species. To accommodate protocol differences, human performance was evaluated using correct rate (CR) and reaction time (RT), whereas mouse performance was assessed using HE accuracy and latency, alongside HE frequency as a measure of repetitive behavior. To facilitate cross-species comparison, the two conditions—10-s and 2-s white noise cues presented to all subjects—are hereafter referred to as “10-s” and “2-s” conditions, respectively, even though task designs were adapted to the behavioral and cognitive characteristics of each species.

### Mouse behavioral data

Behavioral analyses for WT and KO mice included: (1) HE frequency, (2) HE accuracy (hit and correct rejection rates), (3) HE and fastest HE (FHE) peak frequency and latency, and (4) HE window peak frequency and latency derived from segmented latency windows. These indices were used to assess behavioral performance, stimulus discrimination, and temporal decision strategies. Indices (1)–(3) were calculated within individual CS periods, whereas index (4) was based on segmented time windows aligned to CS onset, independent of CS duration. Analyses of (4) were specifically conducted to identify the time point of decision making within 0–14 s after CS onset, encompassing the CS, pre-US, and US periods.

### HE frequency: differences occurred only during CS period

HE frequency was calculated for the CS, pre-US, US, pre-CS, post-CS, and entire period on each training day. Group differences were limited, including during the CS period (Figure 1D). WT mice showed condition-specific differences (10-s vs. 2-s) on D1, D5, D7, D9, and D13–D15, indicating successful cue discrimination. Significant differences were rarely observed during non-CS periods (Supplementary Figure 2A), and total HEs per day did not differ between groups during the late training phase (Figure 1A). These findings indicate that KO mice did not exhibit a generalized increase in responding (Bavley et al., 2018). Overall, HE frequency thus did not reflect habitual behavior in either group. See Supplementary Table 1 for complete statistics.

### HE frequency on initial days (D0 and D1): KO mice adapted faster

Group differences in HE frequencies of merged conditions were observed on D0 (US introduced) and D1 (CS and US introduced). KO mice showed higher HE frequencies than WT mice on both days (Mann–Whitney U test, D0, *Z* = −2.471, *p* = 0.013; D1, *Z* = −2.078, *p* = 0.037; Figure 1E). Within-group comparisons between D0 and D1 were not significant (Wilcoxon signed-ranks test, *p* > 0.05; Supplementary Table 1). These findings suggest that KO mice may have adapted more rapidly to the novel training environment, exhibiting greater engagement and less hesitation, whereas WT mice appeared more cautious during the initial stages of training.

### HE accuracy: both groups learned the task

HE accuracy was calculated from HEs emitted during the 10-s or 2-s CS periods. Both WT and KO mice showed high hit and correct rejection rates, with few significant differences (Figure 1F; Supplementary Figure 2B). During early training (D0–D3, D5/D6 in both groups), correct rejection rates exceeded hit rates (Wilcoxon signed-ranks test, *p* < 0.05). These differences largely disappeared with training, except for a higher hit rate on D16 in WT mice (Supplementary Figure 2C; Supplementary Table 2).

### HE peak: WT and KO mice discriminated with different decision time

HE peak frequency and latency, extracted from the individual HE distributions of all subjects (Kim, 2025), indicate preferred decision times. Both groups showed more HEs in the 10-s condition than in the 2-s condition, reflecting successful discrimination (Figure 1G). HE peak frequency was higher in the 10-s condition on D13–D16 (WT) and D2, D9, D10, D13 (KO), while latency was longer in the 10-s condition on D16 (WT) and D9, D10, D13–D16 (KO) (Wilcoxon signed-ranks test, *p* < 0.05 in all cases). KO mice responded faster to 2-s (1–2 s) than to 10-s (4–6 s) condition, whereas WT mice delayed responding to both cues, typically until 3–5 s. These latency patterns suggest that KO mice tended to initiate responding shortly after cue onset and subsequently adjusted their behavior according to cue duration, whereas WT mice delayed responding before committing to a response. Group differences were limited to D8 (10-s, frequency: WT < KO) and D15 (2-s, latency: WT > KO, Mann–Whitney U test, *p* < 0.05). See Supplementary Table 3 for complete statistics.

### FHE peak: response patterns were similar between groups

Latency and frequency of the first responses following CS onset did not differ between groups. FHE peak measures exhibited minimal variation across training days and experimental conditions (Supplementary Figure 2D; see Kim, 2025 for extraction details). Significant condition differences were observed only on D0 (2-s, frequency) and D4/D9 (2-s, latency; Wilcoxon signed-ranks test, *p* < 0.05). Responses to 10-s and 2-s conditions were otherwise largely comparable, except WT latency on D16. KO mice did not respond immediately to cues. Overall, FHE analyses provided little evidence for immediate cue triggered responding in either group. See Supplementary Table 3 for statistical details.

### HE and FHE peak analyses: KO mice showed delayed responses to 2-s condition

HE and FHE peak frequency patterns were similar across conditions in both groups (Supplementary Figure 3A). Latency differences were generally absent, except in the 10-s condition in KO on D13. In contrast, our previous study found that only the 2-s condition failed to reach significance (Kim, 2025). Correlations between HE and FHE peak frequencies in the 2-s condition during late training (Supplementary Figure 3B) were significant in KO mice but weaker in WT mice. Similarly, correlation between HE and FHE peak latencies in the 2-s condition during late training were also significant in KO mice, indicating premature responding (Supplementary Figure 3C). In HE peak latency, the proportion of KO mice responding within 2 s during the 2-s condition increased across training, whereas WT mice showed no such trend (Supplementary Figure 3D), suggesting persistent responding to the unrewarded 2-s condition in KO mice. See Supplementary Table 3 for full statistics.

### Latency window segmentation: WT mice showed delayed responses compared to KO mice

We analyzed group behavior by tracing individual HE windows across the 14-s period following CS onset. HEs were segmented into fixed latency windows (0–2 s, 0–10 s, and 1-s bins up to 14 s), regardless of condition (Supplementary Figure 4). In the 2-s and 10-s latency window analyses (0–2 s and 0–10 s from CS onset), group differences were not prominent. Condition differences were significant in the 0–10 s window across training days (D1, D4–D6, D8, D9, and D11–D16 for WT mice; D2 and D4–D16 for KO mice; Wilcoxon signed-ranks test, *p* < 0.05 in all cases). In the 0–2 s window, significant differences were observed only on D13 in KO mice and D8 in WT mice (*p* < 0.05; Supplementary Figure 4; Supplementary Table 4). These findings suggest that cue effects were broadly comparable between groups, as both conditions shared the same auditory stimulus during the 0–2 s window and diverged only during the subsequent 2–10 s window.

For 1-s bin analyses, we identified the peak bin from HE traces across the 0–14 s period for each training day (HE window peak; Supplementary Figures 5, 6) and assessed its overlap with bins showing significant group or condition differences (Supplementary Figure 7). WT mice showed HE window peaks at 4–5 s in the 10-s condition, particularly on D14–D15, with overlapping significant differences in both between-group and within-group comparisons (Figures 1H,I). In contrast, KO mice responded earlier, with peaks at 1–2 s in the 2-s condition and 2–3 s in the 10-s condition. The prominent HE window peaks observed in the 10-s condition reflected lower responses compared with the 2-s condition in WT mice and relative to both conditions in KO mice (Supplementary Figure 6). Across D12–D16, HE window peaks in the 10-s condition remained consistently within the 4–5 s bin in WT mice, whereas peaks in the 2-s condition were more variable. During D12–D16, response latencies were longer in WT mice than in KO mice for both conditions, except in the 2-s condition on D12. These temporal patterns indicate that KO mice initiated responding earlier and maintained responding across conditions, whereas WT mice delayed responding, with peak activity concentrated within the 4–5 s window and reduced responding during the 2-s condition. See Supplementary Figure 6 for the frequency and latency of daily peaks, Supplementary Figure 7 and Supplementary Table 5 for the statistics for HE frequencies across 1-s bins (0–14 s).

### Human behavioral data

#### CR and RT: humans paused responses until 2 s

**Figure 2:**
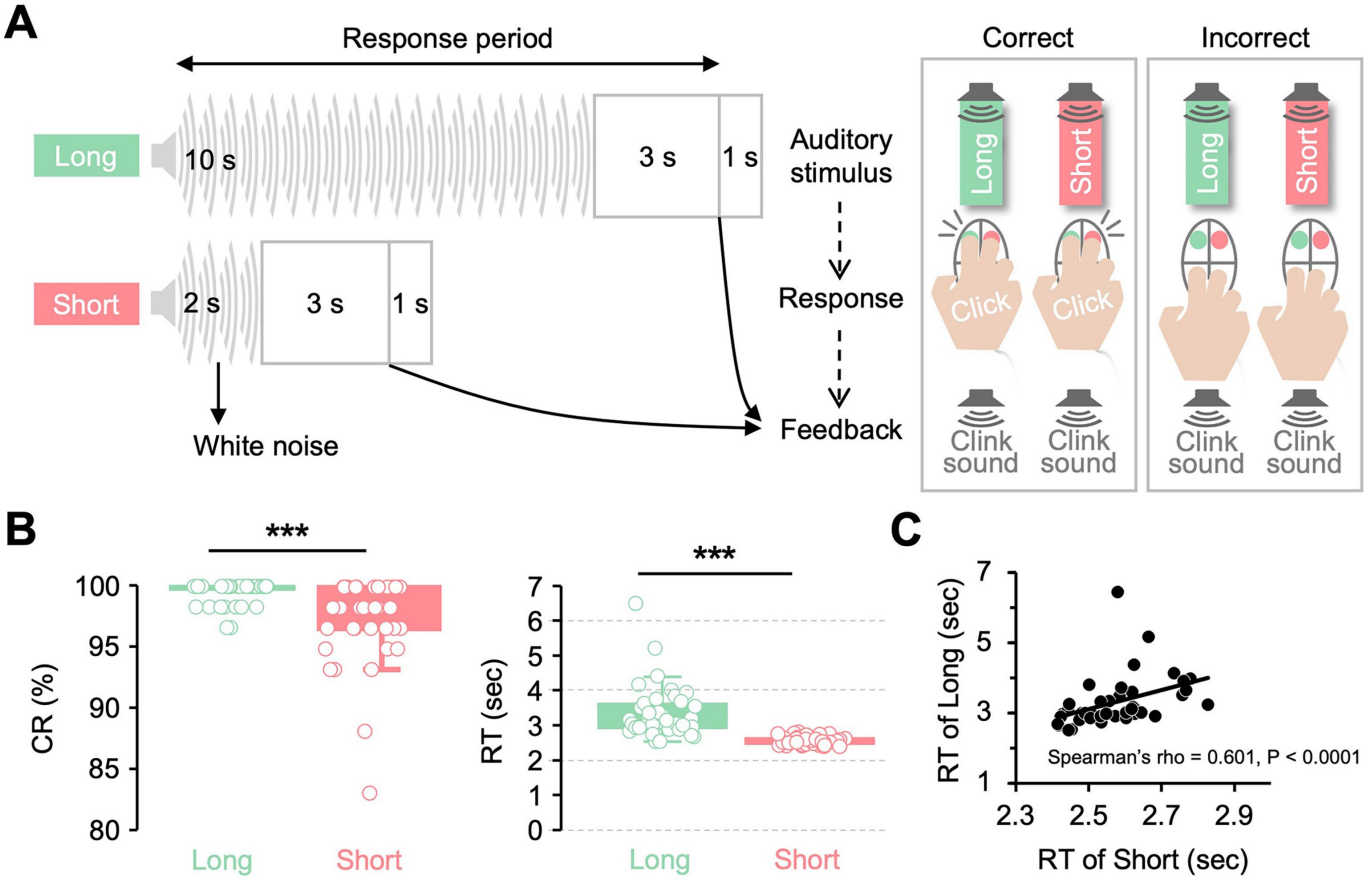
Human experiment and behavioral responses. **(A)** Participants performed a duration discrimination task with 10-s and 2-s conditions. **(B)** Correct rate (CR) was higher in the 10-s condition (99.542 ± 0.924%) than in the 2-s condition (97.333 ± 3.393%; Wilcoxon, *Z* = − 4.107, *p* < 0.0001). Reaction time (RT) was longer in the 10-s condition than in the 2-s condition (*Z* = − 5.511, *p* < 0.0000001). No subject had a mean RT < 2 s. **(C)** RTs of 10-s and 2-s conditions were positively correlated (Spearman’s *ρ* = 0.601, *p* < 0.0001). See Supplementary Table 6 for full statistics. Statistical tests: Wilcoxon signed-rank and Spearman’s rank correlation coefficient; ***p < 0.001 (*N* = 40). In each box plot, the box spans the interquartile range, the line marks the median, and whiskers indicate the most extreme non-outlier values. CR, correct rate; RT, reaction time; Long, 10-s condition; Short, 2-s condition.

In the duration discrimination task (Figure 2A), all participants rated the task as “easy” or “very easy” on a 5-point Likert scale (very easy, easy, neutral, difficult, very difficult). Mean CR was high in both conditions (10-s: 99.542% ± 0.924%; 2-s: 97.333% ± 3.393%), with a small but significant difference in the 10-s condition (Wilcoxon signed-rank test, *Z* = − 4.107, *p* < 0.0001; Figure 2B). Mean RTs were significantly longer in the 10-s condition (3.316 ± 0.739 s) than in the 2-s condition (2.576 ± 0.111 s) (Wilcoxon signed-rank test, *Z* = − 5.511, *p* < 0.0000001; Figure 2B). Notably, all responses occurred after 2 s. Only two participants exhibited RTs > 5 s, and no participant responded within 2 s. Subjective reports indicated that participants typically responded immediately after the 2-s offset in the 2-s condition, whereas in the 10-s condition they continuously monitored elapsed time by estimating the 2-s duration throughout the stimulus presentation. Consistent with these reports, RTs in 10-s and 2-s conditions were positively correlated (Spearman’s *ρ* = 0.601, *p* < 0.0001; Supplementary Table 6).

### Mouse–human comparison

#### RT and latencies: humans and WT mice showed similar pattern

**Figure 3:**
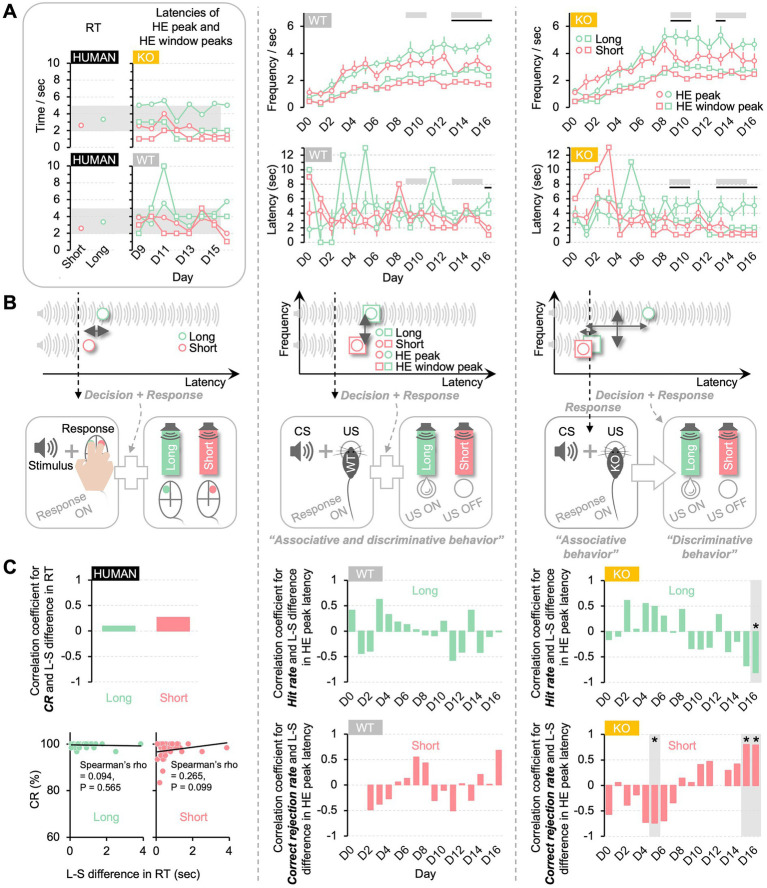
RT/latency and CR/accuracy in humans and mice. **(A)** Human RTs overlapped with the HE peak and HE window latencies of WT mice, but not those of KO mice (gray areas, left). Gray bars (middle, right) mark comparable response periods; black bars mark training periods with significant HE peak differences. **(B)** WT mice showed consistent HE and HE window peaks (> 2 s), similar to humans. KO mice showed inconsistent peaks, with the HE peak delayed beyond 2 s only in the 10-s condition. In WT mice and humans, associative and discriminative processes coincided; in KO mice, they dissociated into two stages. **(C)** The L–S differences in HE latency were correlated with accuracy only in KO mice (Spearman, *N* = 9, *p* < 0.05): latency was negatively correlated with hit rate on D16, positively correlated with correct rejection on D5, D15, D16. No significant correlations in humans (*N* = 40) or WT mice (*N* = 9). See Supplementary Figure 8 and Supplementary Table 7. Error bars: 95% CI. RT, reaction time; HE, head entry; D, training day; Long, 10-s condition; Short, 2-s condition; L–S, Long–Short; WT, wild-type; KO, CRBN KO; CS, conditioned stimulus; US, unconditioned stimulus.

HE peak latency (mean of individual HE peak latencies) and HE window peak latency (HE peak latency of grand average across subjects) were compared over 0–14 s window (Supplementary Figure 5). WT mice showed alignment within 2–5 s latencies in both 10-s and 2-s conditions, paralleling human RTs clustered around 2 s (Figure 3A). In contrast, KO mice showed earlier responses (<2 s) and less consistent HE window peak latencies, alongside delayed HE peak latency for the 10-s condition (Figures 3A,B). These results suggest that individual variability and impulsivity in KO mice influenced group-level HE window peak estimates.

#### Correlation between latency and accuracy: humans and WT mice showed similar pattern

No significant correlation was observed between latency (RT or HE peak latency) and accuracy (CR) (Spearman’s rank correlation coefficient, *p* > 0.05 in all cases) in humans or WT mice. In contrast, the Long–Short difference in the HE peak latency in KO mice exhibited a negative correlation with the hit rate (Spearman’s ρ = − 0.808, *p* = 0.017 on D16; Figure 3C). Regarding the correct rejection rate, it showed a negative correlation on D5 (ρ = − 0.735, *p* = 0.048) but positive correlations on D15 (ρ = 0.798, *p* = 0.020) and D16 (ρ = 0.788, *p* = 0.023) (Figure 3C). Additional analyses using raw RT/latency and CR/accuracy measures yielded consistent results exclusively in KO mice (Supplementary Figure 8). These findings indicate that shorter latencies in KO mice improved No-Go performance but impaired Go performance, consistent with an impulsivity bias. In contrast, humans and WT mice showed stable, delayed response patterns that were not associated with stimulus duration. This suggests that individuals with typical intelligence exhibited more consistent behavioral strategies, showing reduced impulsivity. See Supplementary Table 7 for full statistics.

## Discussion

### Behavioral strategy and RT/latency

**Figure 4:**
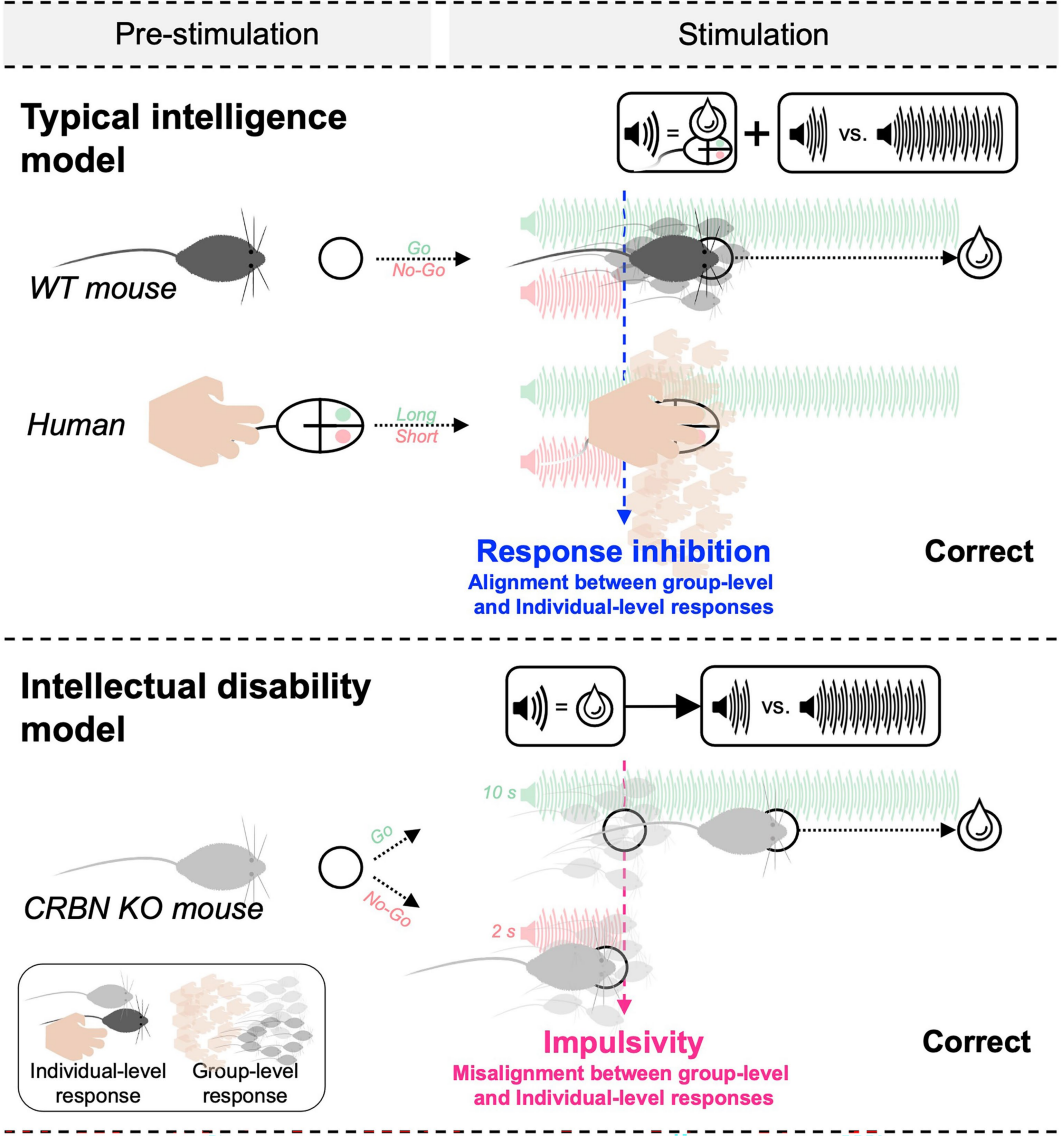
Intelligence-dependent behavioral responses and intelligence-independent perceptual responses. All groups were able to successfully discriminate the stimuli, regardless of intelligence level. However, WT mice and humans with typical intelligence exhibited response inhibition during decision making until 2 s (the offset of the 2-s condition). In contrast, CRBN KO mice with ID showed impulsive responses around 2 s in both 10-s and 2-s conditions, indicating a dissociation between associative and discriminative processes. Furthermore, impulsivity in KO mice led to a misalignment between the group-level response (HE window peak) and the individual-level response (HE peak). In contrast, humans and WT mice exhibited alignment between group-level and individual-level responses.

This study investigated behavioral response patterns across species with typical intelligence and their alterations in the ID phenotype caused by CRBN deficiency. All subjects, regardless of intelligence, discriminated between two auditory conditions, as reflected in accuracy (CR). However, RT and latency in behavioral responses differed between subjects with ID and those with typical intelligence. The objective of the present study was to identify behavioral indices that may capture aspects of human-like behavioral strategies in mice from multiple RT-derived measures (Supplementary Figure 1B). The HE peak, FHE peak, and HE window peak are latency-based measures derived from RT distributions (Kim, 2025). These indices would provide complementary information regarding behavioral inhibition, impulsivity, and behavioral strategies (Burle et al., 2004; de Wit, 2009; Wierda et al., 2025). Behavioral data of latency were interpreted within two frameworks: (1) response inhibition versus impulsivity, and (2) alignment versus misalignment between group-level and individual-level responses (Figures 3, 4). CRBN KO mice exhibited impulsivity and misalignment, whereas WT mice and humans without ID showed response inhibition and alignment. RT and latency measures, including the HE peak and HE window peak, differentiated behavioral patterns (Supplementary Figure 1). This indicates that behavioral strategies in auditory temporal discrimination are clearly reflected in RT and latency measures.

### Perception and behavior

Accuracy (CR) and HE peak frequency reflected recognition of the two conditions across all groups (Figures 1F,G). In mice, a higher HE frequency generally indicates successful task acquisition. HE frequency were not consistent in WT and KO mice, unlike HE peak frequency. In addition, HE frequency during the CS period and HE window peak frequency did not clearly differentiate the two conditions in KO mice (Figures 1D, 3A). These results in KO mice were not due to overresponsiveness (Figure 1A). Behavioral indices were categorized into accuracy (CR), latency (RT), and frequency. In WT mice and humans, accuracy and CR reflected stimulus perception, while latency reflected behavioral strategies. In WT mice, stimulus perception was clearly reflected in all frequency measures. In KO mice, however, perception and behavior were intermixed within the indices: perception was reflected only in HE peak frequency, whereas HE frequency and HE window peak frequency, which were unrelated to perception, likely reflected behavioral artifacts (Supplementary Figure 1C).

### Response inhibition and impulsivity

Impulsive behaviors likely contributed to the behavioral artifacts observed in KO mice (Kagan, 1966; Messer, 1976). The Go/No-Go paradigm assesses both perception and behavior, including impulsivity and response inhibition (Wright et al., 2014; Berditchevskaia et al., 2016). Mice learned both CS–US associations and discrimination between 10-s and 2-s durations during training, but KO mice might initiate premature responses before deciding whether to continue or inhibit responding. These behaviors disrupted the alignment between HE peak frequency and HE window peak frequency (Figure 3A), enhanced HE peak–FHE peak alignment, and increased responses within 2 s during the No-Go condition (Supplementary Figures 3B,D). In a previous study using the same apparatus (Kim, 2025), freely moving C57BL/6NCrljOri mice reached the food cup within 2 s. In contrast, WT mice delayed responses until after 2 s, reflecting response inhibition (Andrzejewski et al., 2011; Caglayan et al., 2021), and typically responded between 2 and 5 s after stimulus onset, allowing simultaneous processing of association and discrimination. Impulsivity has been documented in both animals and humans (Dalley et al., 2011). Human studies have assessed impulsivity using self-report questionnaires (Horn et al., 2003) and meta-analyses (Oosterlaan et al., 1998; Hung et al., 2018). In the present study, a duration discrimination task rather than Go/No-Go paradigm was used to assess human responses. Similar to WT mice, humans paused for approximately 2 s after stimulus onset, implying a comparison of 10-s and 2-s conditions, before responding between 2 and 5 s (Figure 4). This 2-s point marks a critical threshold for response inhibition.

### Group-level and individual-level responses

The “HE peak” reflects the average timing of individual responses, whereas the “HE window peak” reflects the group-level peak from averaged data (Supplementary Figure 5). When individual and group peaks across 1-s bins (0–14 s) coincide, they are considered aligned, indicating alignment between group-level and individual-level responses. WT mice showed such alignment, suggesting shared decision timing, whereas, KO mice exhibited misalignment, reflecting difference between individual and group strategies (Figure 4). The overlap between HE peak and HE window peak within the 2–5 s interval in WT mice resembled the RT pattern in humans. Both species exhibited a delay of approximately 2 s following the stimulus onset, indicative of response inhibition. Group averages often mask individual variability, as demonstrated in studies of insect learnings (Pamir et al., 2011; Lichtenstein et al., 2017; Knebel et al., 2019; Hertel et al., 2020) and human behavioral tasks (Judd et al., 2024). In the present study, differences between group and individual patterns arose from the analytical approaches used (Supplementary Figure 5). The HE window peak was derived from averaged data across all subjects, whereas the HE peak represented the average of individual peaks. HE peak frequencies vary according to the latencies of individual subjects, whereas HE window peak frequency is time-locked. Thus, HE peak and HE window peak serve as indicators of alignment or misalignment between group-level and individual-level behavioral responses.

### Human and mouse

Auditory duration discrimination is an intrinsic ability shared by humans and animals. However, interspecies differences complicate experimental design and interpretation. Even when healthy humans and mice show similar behavioral patterns, methodological differences are unavoidable because of distinct repertoires and extended training required for animals (Supplementary Figure 1A). Humans entered the task with strategies shaped by prior experience, while mice were tested in a fixed cage environment. Humans completed three sessions within a single day, whereas mice underwent a 16-day training protocol that included pre-conditioning (Supplementary Figure 1A). Mice were trained a reward-based Go/No-Go conditioning, whereas humans performed a button-click duration discrimination task without reward contingency. Food reward likely served as a strong motivational factor in the mice, and auditory sensitivity, which is essential for survival, appeared to remain intact even in KO mice with mild ID, enabling accurate perception. However, unlike the HE accuracy observed in mice, CR in human subjects exhibited a distinct difference between 10-s and 2-s conditions (Figure 2). These findings suggest that button-clicking in humans involves a faster and more sensitive motor response mediated by the hand, whereas mice perform a more demanding whole-body response directed toward a food cup. Our findings suggest that the modality of behavioral responses differs fundamentally between humans and mice.

Despite these differences in response modalities—head entry in mice versus mouse click responses in humans—both species converged on a common behavioral strategy within the 2–5 s time window (Figures 3, 4). Human reported using the shorter 2-s condition as a reference, and although mice could not report their strategies, RTs and latency measures in both humans and WT mice reflected comparable behavioral patterns. The approximately 2-s pause indicates response inhibition, suggesting an intrinsic behavioral strategy (Harris and Pressley, 1991) that generalizes across species with typical intelligence while allowing individual variation. These findings indicate that although strategies are learned, the ability to acquire them during task performance is a conserved cross-species trait. The behavioral indices indicate identified similar response patterns rather than identical behavioral paradigms, reflecting specifies-specific adaptation. However, although these results were consistent across species, their generalizability is limited by the small sample size and specific experimental conditions. Additional studies including male subjects will be necessary to provide a more comprehensive understanding.

### ID and CRBN

Previous studies have linked CRBN to deficits in working memory (Gourlat et al., 2024), cognitive performance (Jenni et al., 2015), stress-related behavior (Akber et al., 2022), and depression (Jung et al., 2024), as well as to bipolar disorder, which is associated with impulsivity and impaired decision making (Christodoulou et al., 2006). Our study confirmed impulsive behaviors preceding decision making in CRBN KO mice; however, perceptual discrimination, as reflected by accuracy and HE peak frequency, was preserved. This finding contrasts with the delayed or impaired responses reported in some ID populations (DeFord, 1996; Rattat and Collie, 2020; Gourlat et al., 2024). Furthermore, KO mice did not show abnormal repetitive behavior, consistent with prior findings (Bavley et al., 2018) and unlike observations reported in ASD patients (Boyd et al., 2012).

When first exposed to the US (milk) and CS (75-dB white noise), KO mice were more active than WT mice (Figure 1E). Their learning curve, assessed by HE peak frequency, indicated faster task acquisition (Figure 1G), possibly reflecting fearlessness associated with memory and learning deficits (Rajadhyaksha et al., 2012). Only KO mice showed significant correlation between RT/latency and accuracy measures (Figure 3C). Delayed responses reduced hit rates, while faster responses decreased correct rejection rates, a pattern consistent with impulsive behavior. These behaviors may also reflect reduced neophobia and heightened attention to reward-related cues, supporting robust CS–US associations. Despite their impulsivity, perceptual accuracy remained intact, indicating that the ID phenotype reflects alternative behavioral strategies rather than reduced intelligence. KO mice might simply adopt a different response strategy.

However, these behavioral strategies were not uninformed within the KO group, as evidenced by the misalignment between the individual-level HE peak and group-level HE window peak. From the perspective of WT mice, KO mice responded more impatiently and excessively. The similar timing of response inhibition observed in humans and WT mice reflects comparable behavioral response patterns that were altered in KO mice. Consequently, the impulsivity of KO mice may appear as unexpected behavioral noise when viewed from the WT perspective. In human communication, mutual predictability is essential, and atypical responses of KO mice—and by extension, individuals with ID—may fall outside this implicit predictability. These findings suggest that understanding ID requires focusing not on deficits, limitations, or notions of inferiority, but on differences in behavioral strategies and patterns of behavior. Effective interventions (Yildirim et al., 2010) should begin with a deeper understanding of how individuals approach tasks and respond to tasks.

## Limitation

The Go/No-Go paradigm used in this study, which distinguished between 2-s and 10-s auditory cues, was a relatively simple and stimulus-fixed task compared with those used in previous studies. This paradigm was applied only to mice, whereas humans were tested using the same stimuli but a different behavioral task. Despite these methodological differences—WT mice running toward a food cup versus humans making a button-click response–response latencies were remarkably similar (2–5 s). However, cross-species interpretations should be approached with caution, as time perception and duration estimation may be influenced by species specific factors, including body size, metabolic rate, sensory processing, and behavioral timescales (Healy et al., 2013). Although humans and mice share partially overlapping neural representations of temporal information, the underlying timing mechanisms likely differ across species (Yu et al., 2022). Therefore, the behavioral indices identified in this study may reflect analogous behavioral patterns rather than direct evidence of shared temporal processing mechanisms.

To establish stronger interspecies comparability, future research should test both species using an identical duration discrimination paradigm. In addition. This study includes only female subjects, who are generally more sensitive to auditory cues (Artieda et al., 1992; Krizman et al., 2012; Williams et al., 2015; Conte et al., 2017). Incorporating male cohorts in future works will therefore be important for evaluating potential sex differences.

Although the interpretation that CRBN KO mice exhibit impulsive behavior is supported by the present findings, the current paradigm did not include conventional behavioral assays specifically designed to assess impulsivity. Future studies employing established paradigms, such as the stop-signal task and the five-choice serial reaction time task (5-CSRTT), are warranted to provide additional support for the present interpretation of impulsivity and response inhibition.

In addition, the conclusions of this study are derived primarily from behavioral observations. As a result, the neural mechanisms through which CRBN deficiency influences inhibitory control and decision making remain unclear. Elucidating these mechanisms will require complementary approaches, including neural recordings, circuit-level analyses, and pharmacological investigations. Furthermore, our findings obtained in CRBN KO mice should be replicated in other ID models (Iwase et al., 2016; Golden et al., 2018; Vazquez-Oliver et al., 2020; Araki et al., 2023; Hirai et al., 2023) representing varying degree of ID severity (Patel et al., 2020).

## Conclusion

Time processing, which is essential for survival (Smith and Harper, 2003) and communication (Oxenham, 2018), is known to be impaired in disorders such as autism and schizophrenia (Vatakis and Allman, 2015). ID has also been associated with impairments in time perception (Janeslätt et al., 2010), duration judgment (Rattat and Collie, 2020), and behavioral measures such as RT (Yildirim et al., 2010). Our findings suggest that CRBN mutations causing mild ID (Lee et al., 2010; Sawamura et al., 2015) primarily affect behavior and behavioral strategies rather than perceptual discrimination. Behavioral indices could be categorized into perception- and behavior-related measures; in CRBN KO mice, these components were intermixed, whereas they remained distinct in WT mice. CRBN influenced response inhibition during both sound-reward association and duration discrimination. RT and latency measures revealed similarities in behavioral timing between humans and mice with typical intelligence. Finally, auditory duration discrimination appears to be a fundamental capacity shared across species, and the ability to acquire behavioral strategies during task performance may represent a conserved cross-species characteristics, although its expression may be altered in ID.

## Methods

### Mouse experiment

#### Subjects

The subjects were 18 mice divided into two groups: C57BL/6NCrljOri and CRBN KO mice (*N* = 9 mice per group, aged 7 weeks). All subjects were female, based on evidence that females exhibit greater sensitivity to auditory signals (Artieda et al., 1992; Krizman et al., 2012; Williams et al., 2015; Conte et al., 2017) and to facilitate comparison with the human subjects. All animals were obtained from the Experimental Animal Center at Seoul National Dental University. Each mouse was housed individually and placed on a food-restriction schedule to maintain its body weight at 85% of the normal free-feeding weight throughout the experiment. Water was provided ad libitum. The animals were maintained on a 12-h light–dark cycle, with lights on from 8:00 a.m. to 8:00 p.m. Behavioral training was conducted between 8 and 10 weeks of age. All animal procedures were reviewed and approved by the Institutional Animal Care and Use Committee of Seoul National University (SNU IACUC; SNU-230309-1, SNU-200804-4-1).

#### Apparatus

All experimental procedures were programmed and controlled using MED-PC V software (model SOF-735). Behavioral experiments were conducted in operant conditioning chambers (model EBV-307W-CT, Med Associates; interior dimensions: 21.6 × 17.8 × 12.7 cm). Each chamber was equipped with a stainless steel grid floor consisting of 24 rods (diameter: 0.32 cm; model ENV-307W-GF; floor dimensions: 17.8 × 15.2 × 5.7 cm) and was enclosed within a sound-attenuating MDF cubicle (model ENV-022MD). A house light (28 V DC, 1,000 mA; model ENV-315 W) remained illuminated throughout each session on every training day. Auditory cues were presented at 75 dB (Tosun et al., 2016) using a white noise amplifier (10–25,000 Hz; model ENV-325SW) and were delivered through a speaker mounted inside the chamber (model ENV-324 W). Background noise generated by the ventilation fans within cubicle (model ENV-025F) was approximately 65 dB. Rewards consisted of condensed milk delivered into a 0.5-cc stainless steel receptacle via a liquid pipe (model ENV-303LPHD) connected to a syringe pump (model PHM-100A-EURO). Head entries (HEs) were automatically recorded when mice interrupted the infrared beam within the liquid pipe (detector model ENV-303HDW).

#### Stimulus

In the task, two types of stimuli were used: a conditioned stimulus (CS) and an unconditioned stimulus (US). The CS consisted of white noise presented at two durations, 10-s and 2-s (Figure 1B). These durations were selected based on our previous study (Kim, 2025), which found no significant differences in response frequency or accuracy between the two CS conditions. In the trace conditioning paradigm, the US consisted of a 1-s delivery of condensed milk. The US was delivered only following the 10-s CS, occurring 14–15 s after CS onset, and was never delivered following the 2-s CS. No stimulation was presented during the pre-US interval (i.e., the period between CS offset and US onset). To investigate impulsivity and behavioral strategies underlying decision making, only the 10-s CS was paired with the US. We reasoned that paring the US with the 2-s CS would allow subjects to predict reward after only 2 s, encouraging rapid approach behavior without requiring discrimination of the stimulus duration. In contrast, pairing the US with the 10-s CS required subjects to withhold responding beyond the 2-s time point and evaluate whether the stimulus continued, thereby promoting stimulus-based decision making rather than immediate reward seeking. This design enabled assessment of whether subjects relied on stimulus evaluation rather than immediate reward-seeking responses during duration discrimination. For this reason, we did not include a reversed condition in which the 2-s CS was paired with the US and the 10-s CS was not.

#### Procedure

Given the ID phenotype in CRBN KO mice, we implemented a simplified Go/No-Go paradigm in which a reward of US was provided only in the Go (10-s) trials and no punishment was administered (Figure 1C). Mice were required to determine whether to make an HE response into the food cup during Go trials to obtain a reward. This protocol also permitted repeated responses, allowing assessment of behavioral differences between WT and KO mice throughout operant conditioning, particularly because *CRBN* has been associated with ASD-like repetitive behaviors (Boyd et al., 2012).

Before the main trace conditioning phase, mice underwent an 8-day preconditioning period. During this period, food restriction was applied to maintain body weight at 85% of baseline levels, and mice were acclimated to handling and to the US. On the final day of preconditioning, a session was conducted in the experimental chamber during which the US was delivered without presentation of the CS. The main experiment was conducted over 16 consecutive days (Figure 1A). Each daily session consisted 20 trials, including 10 trials with the 10-s CS and 10 trials with the 2-s CS. The total duration of each session was approximately 1 h per mouse, including preparation time. Intertrial intervals were fixed at 90 s. During each trial, the US (a 1-s delivery of condensed milk) was administered at the end of the Pre-US interval following an HE response, except for the adaptation period (D0–D2). No punishment was administered for missed or premature responses. Mice were allowed to move freely within the chamber throughout the session. The US was delivered via a syringe pump connected to a liquid pipe and food cup, with the pump activated only during the US interval. In this study, a behavioral response directed toward the food cup to consume the reward was defined as a “head entry” (HE) and was recorded with a temporal resolution of 10 ms.

#### Analysis

Data were analyzed from 18 mice (9 CRBN KO and 9 WT mice) under two CS conditions (10-s and 2-s). First, HE frequencies were calculated for individual time windows. Total HE frequency were computed across each session. HE frequencies were also calculated during the CS, pre-US, US, pre-CS, and post-CS periods. The pre-CS period was defined as the 30-s interval preceding CS onset, whereas the post-CS period was defined as the 30-s interval following CS offset. The number of HEs for each subject was normalized to frequency per second. Second, HE accuracy was evaluated by the occurrence of HEs during the CS period in the 10-s and 2-s conditions. Accuracy was calculated on a per-trial basis and expressed as a percentage, with one correct trial corresponding to 10%. Hits and misses were defined based on responses during 10-s CS trials, whereas correct rejection and false alarm were defined based on responses during 2-s CS trials. Third, the frequency and latency values for the HE and first HE (FHE) peaks were extracted for individual subjects in accordance with our previous study (Kim, 2025). The analysis window for both HE and FHE peaks was defined as 0–10 s after CS onset across all groups. HE and FHE peaks reflected the most frequent response timing of individual subjects. Fourth, to characterize changes in HE density pooled across all subjects, we estimated both the frequency and latency of individual peaks within 1-s latency bin from 0 to 14 s, encompassing the CS, pre-US, and US periods. Using these frequency and latency measure, HE window peaks were extracted across the 16 training days and the time windows from 0 to 14 s (see Supplementary Figure 5 for the extraction of HE window peak and HE peak). Unlike the HE peak, the HE window peak reflected time-locked changes in HE frequency. The HE window peak analyses were conducted as follow-up analyses guided by hypotheses derived from significant effects identified around 4 s in the HE peak latency measure. The HE window peak data from all training days and time windows, presented in Supplementary Figures 6, 7 and Supplementary Table 5, are provided as supplementary data supporting interpretation of the 4-s latency effect rather than to identify significant effects through exploratory segmentation. In addition to the 1-s window, HE frequencies were also estimated within the 0–2 s and 0–10 s windows after CS onset to examine the influence of the decision time point on discrimination between the 10-s and 2-s conditions. Using 1-s windows, we assessed whether significant group and condition effects converged within the same temporal bins. Finally, correlation analyses were conducted between individual measures of latency and accuracy.

Behavioral data were non-normally distributed; therefore, all analyses were conducted using nonparametric tests. The analyses focused on genotype differences and condition differences within each genotype, which were assessed separately and independently rather than through genotype × condition interaction analyses as in factorial ANOVA. Within-genotype comparisons (10-s vs. 2-s) were performed using the Wilcoxon signed-rank test, whereas between-genotype comparisons (KO vs. WT) were performed using the Mann–Whitney U test. Correlations were examined using Spearman’s rank correlation coefficient (*α* < 0.05). Multiple comparisons for the two conditions in the correlation analyses and for comparisons between HE and HE peaks were corrected using the Bonferroni method. All analyses were conducted using MATLAB (version 9.12.0.2039608; MathWorks Inc., Natick, MA, USA) and SPSS (version 25.0; IBM, Armonk, NY, USA).

### Human experiment

#### Subjects

Forty healthy participants (mean age = 23.750 ± 1.822 years, range = 21–26 years) were recruited for the study. All participants were female, consistent with the mouse subjects, based on evidence suggesting greater sensitivity to auditory signals in females (Artieda et al., 1992; Krizman et al., 2012; Williams et al., 2015; Conte et al., 2017). Participants were recruited from Seoul National University. The age range was selected to correspond to the developmental stage of the mouse subjects (Dutta and Sengupta, 2016). All participants were right-handed and had normal hearing. On a 5-point Likert scale (very easy, easy, normal, hard, very hard), all participants rated the task as either “very easy” or “easy.” This study was approved by the Institutional Review Board of the Clinical Research Institute, Seoul National University School of Dentistry (Approval No. S-D20210011). All experimental procedures complied with ethical guidelines. Written informed consent was obtained from all participants prior to the experiment. Participants were compensated for their time after completing the task.

#### Procedure

The auditory stimuli consisted of two types of white-noise durations, 10 s and 2 s (referred to as 10-s and 2-s conditions, respectively), identical to those used in the mouse experiment. The Go/No-Go contingency used in the mouse experiment was not adapted for the human experiment. This approach was chosen to better align with the objectives of the mouse study while allowing responses to be collected in both conditions, including 2-s condition, which typically elicited No-Go behavior in mice. To obtain RT measures comparable to the HE data observed in mice, participants were instructed to discriminate between 10-s and 2-s conditions. The experiment comprised three sessions. Each session included 20 trials per condition (10-s and 2-s), presented with a 5 s intertrial interval. In each trial, participants were instructed to click the left mouse button in the 10-s condition and the right mouse button for 2-s condition, using the index and/or middle fingers of their right hand (Figure 2A). Participants were required to make at least one click during sound presentation and before the onset of a subsequent “clink” sound. Before the main task, participants completed a training session consisting of approximately 10 practice trials per condition. Unlike in the mouse experiment, no rewards (e.g., food) were provided. Instead, participants were instructed to concentrate on the task and minimize errors. Following the experiment, participants completed a questionnaire regarding task difficulty and the strategies they used during the task. The total duration of the experiment, including preparation time, was approximately 1 h. Auditory stimuli were generated using STIM2 software (Neuroscan, Charlotte, NC, United States) and presented at 60 dB SPL through stereo headphones (Sony MDR-X50AP).

#### Analysis

Reaction time (RT) and correct rate (CR) were calculated for both 10-s and 2-s conditions. RT values were calculated only from correct trials. For statistical analysis, the mean value across all trials for each subject was computed for each participant. Because the dataset did not consistently satisfy the assumption of normality, nonparametric tests were used, consistent with the analyses conducted for the mouse data. These included the Wilcoxon signed-rank test, the Mann–Whitney U test, and Spearman’s rank correlation coefficient, with a significance threshold of *α* < 0.05. Bonferroni correction was applied for multiple comparisons in the correlation analyses. All analyses were conducted using SPSS (version 25.0; IBM, Armonk, NY, USA).

## Data Availability

The original contributions presented in the study are included in the article/Supplementary material, further inquiries can be directed to the corresponding authors.
