# Supplementary material for: Cereblon-related mild intellectual disability disrupts response inhibition and uniformity of group–individual strategies
Source: Front Neurosci. 2026 Jul 13;20:1782687. doi: 10.3389/fnins.2026.1782687 (PMC13402522; doi:10.3389/fnins.2026.1782687)
Supplement: Supplementary file 1 [file Data_sheet_1.pdf]

Supplementary tables

Table number	Behavioral indices		Ceontets
Supplementary Table 1	HE frequency	1_1	Total HE frequency
		1_2	HE frequency in CS
		1_3	HE frequency in pre-US
		1_4	HE frequency in US
		1_5	HE frequency in CS+pre-US
		1_6	HE frequency in pre-US+US
		1_7	HE frequency in pre-CS and post-CS
		1_8	HE frequency in D0 and D1
Supplementary Table 2	HE accuracy	2_1	Hit and correct rejection rate (CS)
		2_2	Hit and correct rejection rate (CS+pre-US)
		2_3	Hit rate vs. Correction rejection rate (CS)
Supplementary Table 3	HE and FHE peaks	3_1	HE peak frequency
		3_2	HE peak latency
		3_3	FHE peak frequency
		3_4	FHE peak latency
		3_5	HE vs. FHE in frequencies
		3_6	HE vs. FHE in latencies
		3_7	Corrrelation betweeen HE and FHE peak frequencies
		3_8	Corrrelation betweeen HE and FHE peak latencies
		3_9	Correlation between Short and Long in HE peak latency
Supplementary Table 4	HE frequency for 2-s and 10-s latency windows	4_1	HE frequency in 0–2 s window
		4_2	HE frequency in 0–10 s window
Supplementary Table 5	HE frequency for 1-s latency windows	5_1	HE frequency in 0-1 s
		5_2	HE frequency in 1-2 s
		5_3	HE frequency in 2-3 s
		5_4	HE frequency in 3-4 s
		5_5	HE frequency in 4-5 s
		5_6	HE frequency in 5-6 s
		5_7	HE frequency in 6-7 s
		5_8	HE frequency in 7-8 s
		5_9	HE frequency in 8-9 s
		5_10	HE frequency in 9-10 s
		5_11	HE frequency in 10-11 s
		5_12	HE frequency in 11-12 s
		5_13	HE frequency in 12-13 s
		5_14	HE frequency in 13-14 s
Supplementary Table 6	CR and RT	6_1	CR
		6_2	RT
		6_3	Correlation between Long and Short in RT
Supplementary Table 7	Correlation between RT/Latency and CR/Accuracy	7_1	Human: Correlation between LATENCY (L–S difference in RT or RT) and ACCURACY (CR)
		7_2	Mouse: Correlation between LATENCY (L–S difference in HE peak latency) and ACCURACY (hit and correct rejection rates)
		7_3	Mouse: Correlation between LATENCY (HE peak latency) and ACCURACY (hit and correct rejection rates)

Abbreviations: HE, head entry; CS, conditioned stimulus; US, unconditioned stimulus; CR, correct rate; RT, reaction time.

1_1

Total HE frequency									
Table	Related Figure	Day	Group	Mean	SEM	Stastistical test	Z	P	*
Table S1	Figure 1D	D0	KO	54.722	8.545	Mann-Whitney U Test	-1.988	0.047	*
			WT	30.000	10.141				
		D1	KO	76.778	10.768		-1.634	0.102	
			WT	47.389	9.368				
		D2	KO	95.222	10.613		-2.518	0.012	*
			WT	68.778	4.930				
		D3	KO	108.778	16.707		-0.221	0.825	
			WT	125.778	17.680				
		D4	KO	139.111	10.961		0.000	1.000	
			WT	148.889	12.382				
		D5	KO	119.222	10.801		-0.751	0.453	
			WT	142.889	18.461				
		D6	KO	175.222	23.493		-1.810	0.070	
			WT	120.556	11.237				
		D7	KO	153.333	19.258		-0.044	0.965	
			WT	153.667	18.066				
		D8	KO	177.111	27.648		-0.442	0.659	
			WT	153.000	13.015				
		D9	KO	175.556	32.384		-0.486	0.627	
			WT	149.111	18.530				
		D10	KO	129.000	14.764		-0.221	0.825	
			WT	130.778	20.487				
		D11	KO	129.111	15.123		-0.044	0.965	
			WT	137.222	23.956				
		D12	KO	123.333	15.343		-0.132	0.895	
			WT	125.556	14.588				
		D13	KO	115.889	19.062		0.000	1.000	
			WT	106.111	9.398				
		D14	KO	124.444	13.474		-0.575	0.566	
			WT	106.000	9.985				
		D15	KO	103.111	13.007		-0.619	0.536	
			WT	114.111	14.496				
		D16	KO	105.000	13.089		-0.044	0.965	
			WT	100.556	10.205				

Supplementary Table 1. HE frequency. Differences between groups and conditions, focusing on CS, Pre-CS, and US periods, were analyzed separately. Analyses were performed using the Mann–Whitney U test and Wilcoxon signed-rank test. *p < 0.05 (N = 9). Abbreviations: HE, head entry; D, training day; KO, CRBN KO mice; WT, wild-type mice; CS, conditioned stimulus; US, unconditioned stimulus.

1_2

HE frequency in CS										
Table	Related Figure	Day	Conditon	Group	Mean	SEM	Stastistical test	Z	P	*
Table S1	Figure 1D	D0	Long	KO	0.228	0.090	Mann-Whitney U Test	-1.683	0.113	
				WT	0.072	0.032				
		D1	Long	KO	0.161	0.058		-0.544	0.605	
				WT	0.111	0.045				
		D2	Long	KO	0.272	0.082		-0.359	0.730	
				WT	0.256	0.103				
		D3	Long	KO	0.544	0.161		-0.671	0.546	
				WT	0.400	0.133				
		D4	Long	KO	0.811	0.176		-0.488	0.666	
				WT	0.878	0.179				
		D5	Long	KO	0.756	0.199		-0.490	0.666	
				WT	0.856	0.209				
		D6	Long	KO	1.356	0.209		-1.681	0.094	
				WT	0.900	0.142				
		D7	Long	KO	1.233	0.255		-0.312	0.796	
				WT	1.144	0.166				
		D8	Long	KO	2.133	0.378		-1.819	0.077	
				WT	1.433	0.181				
		D9	Long	KO	2.256	0.365		-1.459	0.161	
				WT	1.567	0.297				
		D10	Long	KO	1.833	0.370		-0.355	0.730	
				WT	1.589	0.263				
		D11	Long	KO	1.856	0.452		-0.177	0.863	
				WT	1.689	0.337				
		D12	Long	KO	1.678	0.213		-0.354	0.730	
				WT	1.756	0.221				
		D13	Long	KO	1.811	0.328		-0.354	0.730	
				WT	1.622	0.179				
		D14	Long	KO	1.700	0.260		-0.486	0.666	
				WT	1.856	0.221				
		D15	Long	KO	1.711	0.280		-0.267	0.796	
				WT	1.744	0.221				
		D16	Long	KO	1.522	0.146		-0.665	0.546	
				WT	1.600	0.166				
Table S1	Figure 1D	D0	Short	KO	0.139	0.084	Mann-Whitney U Test	-1.032	0.489	
				WT	0.056	0.056				
		D1	Short	KO	0.306	0.116		-2.523	0.050	
				WT	0.000	0.000				
		D2	Short	KO	0.444	0.123		-1.518	0.161	
				WT	0.194	0.069				
		D3	Short	KO	0.556	0.176		-0.278	0.796	
				WT	0.611	0.162				
		D4	Short	KO	1.111	0.341		-0.495	0.666	
				WT	1.111	0.217				
		D5	Short	KO	0.833	0.186		-0.285	0.796	
				WT	0.778	0.206				
		D6	Short	KO	0.944	0.212		-1.423	0.190	
				WT	0.556	0.212				
		D7	Short	KO	1.500	0.354		-1.606	0.161	
				WT	0.667	0.083				
		D8	Short	KO	1.444	0.475		-0.270	0.796	
				WT	1.111	0.247				
		D9	Short	KO	1.778	0.426		-2.303	0.024	*
				WT	0.667	0.144				
		D10	Short	KO	1.444	0.306		-1.097	0.297	
				WT	0.944	0.242				
		D11	Short	KO	1.278	0.426		-0.136	0.931	
				WT	1.111	0.217				
		D12	Short	KO	1.444	0.327		-0.814	0.436	
				WT	1.056	0.242				
		D13	Short	KO	1.389	0.415		-1.078	0.297	
				WT	0.722	0.147				
		D14	Short	KO	1.889	0.423		-1.653	0.113	
				WT	1.000	0.250				
		D15	Short	KO	1.833	0.583		-1.033	0.340	
				WT	0.833	0.264				
		D16	Short	KO	1.667	0.486		-0.760	0.489	
				WT	1.056	0.227				

HE frequency in CS										
Table	Related Figure	Day	Group	Condition	Mean	SEM	Stastistical test	Z	P	*
Table S1	Figure 1D	D0	KO	Long	0.228	0.090	Wilcoxon Signed Ranks Test	-0.593	0.553	
				Short	0.139	0.084				
		D1	KO	Long	0.161	0.058		-1.014	0.310	
				Short	0.306	0.116				
		D2	KO	Long	0.272	0.082		-0.946	0.344	
				Short	0.444	0.123				
		D3	KO	Long	0.544	0.161		-0.070	0.944	
				Short	0.556	0.176				
		D4	KO	Long	0.811	0.176		-0.845	0.398	
				Short	1.111	0.341				
		D5	KO	Long	0.756	0.199		-0.560	0.575	
				Short	0.833	0.186				
		D6	KO	Long	1.356	0.209		-1.244	0.214	
				Short	0.944	0.212				
		D7	KO	Long	1.233	0.255		-0.772	0.440	
				Short	1.500	0.354				
		D8	KO	Long	2.133	0.378		-1.332	0.183	
				Short	1.444	0.475				
		D9	KO	Long	2.256	0.365		-1.122	0.262	
				Long	1.778	0.426				
		D10	KO	Short	1.833	0.370		-1.547	0.122	
				Long	1.444	0.306				
		D11	KO	Short	1.856	0.452		-1.542	0.123	
				Long	1.278	0.426				
		D12	KO	Short	1.678	0.213		-0.833	0.405	
				Long	1.444	0.327				
		D13	KO	Short	1.811	0.328		-1.483	0.138	
				Long	1.389	0.415				
		D14	KO	Short	1.700	0.260		-0.593	0.553	
				Long	1.889	0.423				
		D15	KO	Short	1.711	0.280		-0.296	0.767	
				Long	1.833	0.583				
		D16	KO	Short	1.522	0.146		0.000	1.000	
				Long	1.667	0.486				
Table S1	Figure 1D	D0	WT	Long	0.072	0.032	Wilcoxon Signed Ranks Test	-0.677	0.498	
				Short	0.056	0.056				
		D1	WT	Long	0.111	0.045		-2.214	0.027	*
				Short	0.000	0.000				
		D2	WT	Long	0.256	0.103		0.000	1.000	
				Short	0.194	0.069				
		D3	WT	Long	0.400	0.133		-1.892	0.058	
				Short	0.611	0.162				
		D4	WT	Long	0.878	0.179		-2.120	0.034	*
				Short	1.111	0.217				
		D5	WT	Long	0.856	0.209		-0.416	0.677	
				Short	0.778	0.206				
		D6	WT	Long	0.900	0.142		-1.334	0.182	
				Short	0.556	0.212				
		D7	WT	Long	1.144	0.166		-2.077	0.038	*
				Short	0.667	0.083				
		D8	WT	Long	1.433	0.181		-0.912	0.362	
				Short	1.111	0.247				
		D9	WT	Long	1.567	0.297		-2.366	0.018	*
				Long	0.667	0.144				
		D10	WT	Short	1.589	0.263		-1.727	0.084	
				Long	0.944	0.242				
		D11	WT	Short	1.689	0.337		-1.365	0.172	
				Long	1.111	0.217				
		D12	WT	Short	1.756	0.221		-1.823	0.068	
				Long	1.056	0.242				
		D13	WT	Short	1.622	0.179		-2.524	0.012	*
				Long	0.722	0.147				
		D14	WT	Short	1.856	0.221		-2.429	0.015	*
				Long	1.000	0.250				
		D15	WT	Short	1.744	0.221		-2.312	0.021	*
				Long	0.833	0.264				
		D16	WT	Short	1.600	0.166		-1.755	0.079	
				Long	1.056	0.227				

1_3

HE frequency in pre-US										
Table	Related Figure	Day	Conditon	Group	Mean	SEM	Stastistical test	Z	P	*
Table S1	Figure S2A	D0	Long	KO	0.222	0.073	Mann-Whitney U Test	-1.415	0.222	
				WT	0.370	0.331				
		D1	Long	KO	0.093	0.049		0.000	1.000	
				WT	0.093	0.049				
		D2	Long	KO	0.259	0.104		-1.222	0.297	
				WT	0.074	0.040				
		D3	Long	KO	0.333	0.147		-1.218	0.258	
				WT	0.630	0.171				
		D4	Long	KO	0.889	0.385		-0.585	0.605	
				WT	0.815	0.158				
		D5	Long	KO	1.259	0.265		-1.348	0.190	
				WT	0.778	0.229				
		D6	Long	KO	1.407	0.411		-1.717	0.094	
				WT	0.444	0.124				
		D7	Long	KO	1.222	0.503		-0.539	0.605	
				WT	0.852	0.300				
		D8	Long	KO	1.593	0.293		-1.249	0.222	
				WT	1.074	0.318				
		D9	Long	KO	1.815	0.545		-0.310	0.796	
				WT	1.630	0.522				
		D10	Long	KO	0.815	0.420		-0.722	0.489	
				WT	1.222	0.423				
		D11	Long	KO	0.778	0.397		-0.857	0.436	
				WT	1.741	0.724				
		D12	Long	KO	1.111	0.550		-0.405	0.730	
				WT	1.000	0.329				
		D13	Long	KO	1.000	0.272		-0.134	0.931	
				WT	1.000	0.329				
		D14	Long	KO	0.667	0.347		-1.419	0.190	
				WT	1.037	0.312				
		D15	Long	KO	0.963	0.349		-0.139	0.931	
				WT	1.111	0.419				
		D16	Long	KO	1.222	0.441		-0.319	0.796	
				WT	0.889	0.324				
Table S1	Figure S2A	D0	Short	KO	0.130	0.037	Mann-Whitney U Test	-0.576	0.605	
				WT	0.111	0.056				
		D1	Short	KO	0.407	0.125		-2.146	0.050	
				WT	0.074	0.049				
		D2	Short	KO	0.370	0.117		-0.047	1.000	
				WT	0.352	0.098				
		D3	Short	KO	0.667	0.176		-0.181	0.863	
				WT	0.630	0.161				
		D4	Short	KO	0.963	0.171		-1.803	0.077	
				WT	0.519	0.148				
		D5	Short	KO	1.296	0.245		-0.179	0.863	
				WT	1.222	0.351				
		D6	Short	KO	1.333	0.266		-1.121	0.297	
				WT	0.926	0.271				
		D7	Short	KO	1.333	0.208		-0.134	0.931	
				WT	1.259	0.253				
		D8	Short	KO	2.037	0.321		-2.046	0.040	*
				WT	1.185	0.201				
		D9	Short	KO	1.667	0.289		-0.134	0.931	
				WT	1.630	0.232				
		D10	Short	KO	1.593	0.341		-0.089	0.931	
				WT	1.630	0.275				
		D11	Short	KO	1.704	0.406		-0.045	1.000	
				WT	1.593	0.265				
		D12	Short	KO	1.370	0.344		-0.223	0.863	
				WT	1.370	0.280				
		D13	Short	KO	1.556	0.248		-1.235	0.258	
				WT	1.333	0.079				
		D14	Short	KO	1.370	0.251		-0.947	0.387	
				WT	0.963	0.232				
		D15	Short	KO	1.074	0.241		-0.178	0.863	
				WT	1.111	0.289				
		D16	Short	KO	1.370	0.225		-0.458	0.666	
				WT	1.148	0.137				

HE frequency in pre-US										
Table	Related Figure	Day	Group	Condition	Mean	SEM	Stastistical test	Z	P	*
Table S1	Figure S2A	D0	KO	Long	0.222	0.073	Wilcoxon Signed Ranks Test	-0.850	0.395	
				Short	0.130	0.037				
		D1	KO	Long	0.093	0.049		-1.980	0.048	
				Short	0.407	0.125				
		D2	KO	Long	0.259	0.104		-0.948	0.343	
				Short	0.370	0.117				
		D3	KO	Long	0.333	0.147		-1.279	0.201	
				Short	0.667	0.176				
		D4	KO	Long	0.889	0.385		-0.943	0.345	
				Short	0.963	0.171				
		D5	KO	Long	1.259	0.265		-0.106	0.916	
				Short	1.296	0.245				
		D6	KO	Long	1.407	0.411		0.000	1.000	
				Short	1.333	0.266				
		D7	KO	Long	1.222	0.503		-0.593	0.553	
				Short	1.333	0.208				
		D8	KO	Long	1.593	0.293		-1.126	0.260	
				Short	2.037	0.321				
		D9	KO	Long	1.815	0.545		-0.526	0.599	
				Long	1.667	0.289				
		D10	KO	Short	0.815	0.420		-1.550	0.121	
				Long	1.593	0.341				
		D11	KO	Short	0.778	0.397		-1.963	0.050	
				Long	1.704	0.406				
		D12	KO	Short	1.111	0.550		-1.193	0.233	
				Long	1.370	0.344				
		D13	KO	Short	1.000	0.272		-1.691	0.091	
				Long	1.556	0.248				
		D14	KO	Short	0.667	0.347		-1.548	0.122	
				Long	1.370	0.251				
		D15	KO	Short	0.963	0.349		-0.179	0.858	
				Long	1.074	0.241				
		D16	KO	Short	1.222	0.441		-0.178	0.858	
				Long	1.370	0.225				
Table S1	Figure S2A	D0	WT	Long	0.370	0.331	Wilcoxon Signed Ranks Test	-0.447	0.655	
				Short	0.111	0.056				
		D1	WT	Long	0.093	0.049		-0.141	0.888	
				Short	0.074	0.049				
		D2	WT	Long	0.074	0.040		-2.214	0.027	*
				Short	0.352	0.098				
		D3	WT	Long	0.630	0.171		-0.086	0.931	
				Short	0.630	0.161				
		D4	WT	Long	0.815	0.158		-1.347	0.178	
				Short	0.519	0.148				
		D5	WT	Long	0.778	0.229		-1.897	0.058	
				Short	1.222	0.351				
		D6	WT	Long	0.444	0.124		-1.420	0.156	
				Short	0.926	0.271				
		D7	WT	Long	0.852	0.300		-1.606	0.108	
				Short	1.259	0.253				
		D8	WT	Long	1.074	0.318		-0.210	0.833	
				Short	1.185	0.201				
		D9	WT	Long	1.630	0.522		-0.059	0.953	
				Long	1.630	0.232				
		D10	WT	Short	1.222	0.423		-0.847	0.397	
				Long	1.630	0.275				
		D11	WT	Short	1.741	0.724		-0.070	0.944	
				Long	1.593	0.265				
		D12	WT	Short	1.000	0.329		-1.016	0.310	
				Long	1.370	0.280				
		D13	WT	Short	1.000	0.329		-1.018	0.309	
				Long	1.333	0.079				
		D14	WT	Short	1.037	0.312		-0.135	0.893	
				Long	0.963	0.232				
		D15	WT	Short	1.111	0.419		-0.059	0.953	
				Long	1.111	0.289				
		D16	WT	Short	0.889	0.324		-0.895	0.371	
				Long	1.148	0.137				

1_4

HE frequency in US

Table	Related Figure	Day	Conditon	Group	Mean	SEM	Stastistical test	Z	P	*
Table S1	Figure S2A	D0	Long	KO	0.000	0.000	Mann-Whitney U Test	-1.000	0.730	
				WT	0.056	0.056				
		D1	Long	KO	0.167	0.118		-0.544	0.730	
				WT	0.111	0.111				
		D2	Long	KO	0.389	0.232		-0.503	0.666	
				WT	0.556	0.256				
		D3	Long	KO	0.333	0.236		0.000	1.000	
				WT	0.333	0.236				
		D4	Long	KO	1.000	0.333		-0.095	0.931	
				WT	0.889	0.261				
		D5	Long	KO	0.444	0.242		-1.219	0.297	
				WT	1.667	0.726				
		D6	Long	KO	1.111	0.351		-1.046	0.340	
				WT	0.778	0.465				
		D7	Long	KO	0.556	0.242		-1.068	0.387	
				WT	0.222	0.147				
		D8	Long	KO	1.222	0.662		-0.190	0.863	
				WT	1.000	0.408				
		D9	Long	KO	0.889	0.512		-0.158	0.931	
				WT	1.111	0.655				
		D10	Long	KO	1.222	0.662		-0.252	0.863	
				WT	0.889	0.455				
		D11	Long	KO	1.000	0.373		-0.512	0.666	
				WT	1.444	0.530				
		D12	Long	KO	0.556	0.556		-0.910	0.546	
				WT	0.556	0.338				
		D13	Long	KO	0.667	0.441		-0.527	0.666	
				WT	0.667	0.289				
		D14	Long	KO	0.444	0.338		-0.680	0.666	
				WT	0.111	0.111				
		D15	Long	KO	0.556	0.338		-0.620	0.666	
				WT	0.222	0.147				
		D16	Long	KO	0.778	0.324		-0.591	0.605	
				WT	0.444	0.176				
Table S1	Figure S2A	D0	Short	KO	0.056	0.056	Mann-Whitney U Test	-1.627	0.222	
				WT	0.333	0.144				
		D1	Short	KO	0.278	0.147		0.000	1.000	
				WT	0.278	0.147				
		D2	Short	KO	0.500	0.204		-0.911	0.436	
				WT	0.222	0.121				
		D3	Short	KO	0.556	0.338		-0.264	0.863	
				WT	0.889	0.455				
		D4	Short	KO	0.444	0.176		-1.380	0.222	
				WT	1.444	0.475				
		D5	Short	KO	0.778	0.278		-1.101	0.340	
				WT	1.222	0.278				
		D6	Short	KO	1.111	0.423		-1.669	0.136	
				WT	0.444	0.338				
		D7	Short	KO	1.333	0.236		-0.047	1.000	
				WT	1.333	0.333				
		D8	Short	KO	1.667	0.707		-0.232	0.863	
				WT	0.889	0.261				
		D9	Short	KO	0.778	0.278		0.000	1.000	
				WT	0.778	0.278				
		D10	Short	KO	1.444	0.475		-0.552	0.605	
				WT	1.000	0.289				
		D11	Short	KO	0.889	0.389		-0.949	0.436	
				WT	0.444	0.338				
		D12	Short	KO	1.222	0.364		-0.233	0.863	
				WT	1.333	0.373				
		D13	Short	KO	0.778	0.278		-0.687	0.546	
				WT	0.556	0.294				
		D14	Short	KO	0.778	0.222		-2.004	0.063	
				WT	1.889	0.423				
		D15	Short	KO	0.889	0.351		-0.191	0.863	
				WT	0.778	0.324				
		D16	Short	KO	0.556	0.176		-0.653	0.605	
				WT	0.444	0.242				

HE frequency in US

Table	Related Figure	Day	Group	Condition	Mean	SEM	Stastistical test	Z	P	*
Table S1	Figure S2A	D0	KO	Long	0.000	0.000	Wilcoxon Signed Ranks Test	-1.000	0.317	
				Short	0.056	0.056				
		D1	KO	Long	0.167	0.118		-0.552	0.581	
				Short	0.278	0.147				
		D2	KO	Long	0.389	0.232		-0.557	0.577	
				Short	0.500	0.204				
		D3	KO	Long	0.333	0.236		-0.378	0.705	
				Short	0.556	0.338				
		D4	KO	Long	1.000	0.333		-1.186	0.236	
				Short	0.444	0.176				
		D5	KO	Long	0.444	0.242		-1.134	0.257	
				Short	0.778	0.278				
		D6	KO	Long	1.111	0.351		0.000	1.000	
				Short	1.111	0.423				
		D7	KO	Long	0.556	0.242		-2.333	0.020	*
				Short	1.333	0.236				
		D8	KO	Long	1.222	0.662		-0.690	0.490	
				Short	1.667	0.707				
		D9	KO	Long	0.889	0.512		0.000	1.000	
				Long	0.778	0.278				
		D10	KO	Short	1.222	0.662		-0.324	0.746	
				Long	1.444	0.475				
		D11	KO	Short	1.000	0.373		-0.184	0.854	
				Long	0.889	0.389				
		D12	KO	Short	0.556	0.556		-0.954	0.340	
				Long	1.222	0.364				
		D13	KO	Short	0.667	0.441		-0.171	0.864	
				Long	0.778	0.278				
		D14	KO	Short	0.444	0.338		-0.791	0.429	
				Long	0.778	0.222				
		D15	KO	Short	0.556	0.338		-0.647	0.518	
				Long	0.889	0.351				
		D16	KO	Short	0.778	0.324		-0.707	0.480	
				Long	0.556	0.176				
Table S1	Figure S2A	D0	WT	Long	0.056	0.056	Wilcoxon Signed Ranks Test	-1.518	0.129	
				Short	0.333	0.144				
		D1	WT	Long	0.111	0.111		-0.756	0.450	
				Short	0.278	0.147				
		D2	WT	Long	0.556	0.256		-1.633	0.102	
				Short	0.222	0.121				
		D3	WT	Long	0.333	0.236		-1.289	0.197	
				Short	0.889	0.455				
		D4	WT	Long	0.889	0.261		-0.850	0.396	
				Short	1.444	0.475				
		D5	WT	Long	1.667	0.726		-0.514	0.607	
				Short	1.222	0.278				
		D6	WT	Long	0.778	0.465		-0.542	0.588	
				Short	0.444	0.338				
		D7	WT	Long	0.222	0.147		-2.271	0.023	*
				Short	1.333	0.333				
		D8	WT	Long	1.000	0.408		-0.108	0.914	
				Short	0.889	0.261				
		D9	WT	Long	1.111	0.655		-0.422	0.673	
				Long	0.778	0.278				
		D10	WT	Short	0.889	0.455		-0.172	0.863	
				Long	1.000	0.289				
		D11	WT	Short	1.444	0.530		-1.841	0.066	
				Long	0.444	0.338				
		D12	WT	Short	0.556	0.338		-1.063	0.288	
				Long	1.333	0.373				
		D13	WT	Short	0.667	0.289		-0.276	0.783	
				Long	0.556	0.294				
		D14	WT	Short	0.111	0.111		-2.555	0.011	*
				Long	1.889	0.423				
		D15	WT	Short	0.222	0.147		-1.265	0.206	
				Long	0.778	0.324				
		D16	WT	Short	0.444	0.176		0.000	1.000	
				Long	0.444	0.242				

1_5

HE frequency in CS + pre-US

Table	Related Figure	Day	Conditon	Group	Mean	SEM	Stastistical test	Z	P	*
Table S1	Figure S2A	D0	Long	KO	0.226	0.084	Mann-Whitney U Test	-1.656	0.113	
				WT	0.141	0.099				
		D1	Long	KO	0.145	0.054		-0.361	0.730	
				WT	0.107	0.042				
		D2	Long	KO	0.269	0.075		-0.535	0.605	
				WT	0.214	0.078				
		D3	Long	KO	0.487	0.130		-0.223	0.863	
				WT	0.453	0.130				
		D4	Long	KO	0.829	0.199		-0.133	0.931	
				WT	0.863	0.152				
		D5	Long	KO	0.872	0.187		-0.311	0.796	
				WT	0.829	0.187				
		D6	Long	KO	1.368	0.237		-1.642	0.113	
				WT	0.795	0.113				
		D7	Long	KO	1.231	0.268		-0.222	0.863	
				WT	1.077	0.182				
		D8	Long	KO	2.009	0.323		-2.210	0.024	*
				WT	1.350	0.202				
		D9	Long	KO	2.154	0.354		-1.284	0.222	
				WT	1.581	0.329				
		D10	Long	KO	1.598	0.299		-0.310	0.796	
				WT	1.504	0.269				
		D11	Long	KO	1.607	0.391		-0.133	0.931	
				WT	1.701	0.404				
		D12	Long	KO	1.538	0.248		0.000	1.000	
				WT	1.581	0.190				
		D13	Long	KO	1.624	0.270		-0.089	0.931	
				WT	1.479	0.185				
		D14	Long	KO	1.462	0.231		-0.531	0.605	
				WT	1.667	0.208				
		D15	Long	KO	1.538	0.198		-0.177	0.863	
				WT	1.598	0.220				
		D16	Long	KO	1.453	0.158		-0.398	0.730	
				WT	1.427	0.181				
Table S1	Figure S2A	D0	Short	KO	0.133	0.041	Mann-Whitney U Test	-1.122	0.297	
				WT	0.089	0.048				
		D1	Short	KO	0.367	0.109		-2.507	0.019	*
				WT	0.044	0.029				
		D2	Short	KO	0.400	0.099		-0.813	0.436	
				WT	0.289	0.065				
		D3	Short	KO	0.600	0.133		-0.091	0.931	
				WT	0.622	0.122				
		D4	Short	KO	1.000	0.186		-1.541	0.136	
				WT	0.756	0.104				
		D5	Short	KO	1.111	0.180		-0.621	0.546	
				WT	1.044	0.262				
		D6	Short	KO	1.156	0.194		-1.336	0.190	
				WT	0.778	0.209				
		D7	Short	KO	1.400	0.240		-0.905	0.387	
				WT	1.000	0.149				
		D8	Short	KO	1.778	0.261		-1.957	0.050	
				WT	1.156	0.152				
		D9	Short	KO	1.711	0.304		-1.378	0.190	
				WT	1.244	0.172				
		D10	Short	KO	1.533	0.269		-0.715	0.489	
				WT	1.333	0.170				
		D11	Short	KO	1.533	0.320		-0.133	0.931	
				WT	1.400	0.231				
		D12	Short	KO	1.400	0.240		-0.668	0.546	
				WT	1.244	0.124				
		D13	Short	KO	1.489	0.186		-1.935	0.063	
				WT	1.089	0.075				
		D14	Short	KO	1.578	0.209		-2.002	0.050	
				WT	0.978	0.122				
		D15	Short	KO	1.378	0.310		-1.201	0.258	
				WT	1.000	0.167				
		D16	Short	KO	1.489	0.214		-2.229	0.031	
				WT	1.111	0.068				

HE frequency in CS + pre-US

Table	Related Figure	Day	Group	Condition	Mean	SEM	Stastistical test	Z	P	*
Table S1	Figure S2A	D0	KO	Long	0.226	0.084	Wilcoxon Signed Ranks Test	-1.008	0.314	
				Short	0.133	0.041				
		D1	KO	Long	0.145	0.054		-1.540	0.123	
				Short	0.367	0.109				
		D2	KO	Long	0.269	0.075		-1.823	0.068	
				Short	0.400	0.099				
		D3	KO	Long	0.487	0.130		-0.420	0.674	
				Short	0.600	0.133				
		D4	KO	Long	0.829	0.199		-1.352	0.176	
				Short	1.000	0.186				
		D5	KO	Long	0.872	0.187		-1.718	0.086	
				Short	1.111	0.180				
		D6	KO	Long	1.368	0.237		-0.770	0.441	
				Short	1.156	0.194				
		D7	KO	Long	1.231	0.268		-1.007	0.314	
				Short	1.400	0.240				
		D8	KO	Long	2.009	0.323		-1.007	0.314	
				Short	1.778	0.261				
		D9	KO	Long	2.154	0.354		-1.960	0.050	
				Long	1.711	0.304				
		D10	KO	Short	1.598	0.299		-0.059	0.953	
				Long	1.533	0.269				
		D11	KO	Short	1.607	0.391		-0.593	0.553	
				Long	1.533	0.320				
		D12	KO	Short	1.538	0.248		-0.356	0.722	
				Long	1.400	0.240				
		D13	KO	Short	1.624	0.270		-0.420	0.674	
				Long	1.489	0.186				
		D14	KO	Short	1.462	0.231		-0.474	0.635	
				Long	1.578	0.209				
		D15	KO	Short	1.538	0.198		-0.296	0.767	
				Long	1.378	0.310				
		D16	KO	Short	1.453	0.158		-0.178	0.859	
				Long	1.489	0.214				
Table S1	Figure S2A	D0	WT	Long	0.141	0.099	Wilcoxon Signed Ranks Test	-0.135	0.893	
				Short	0.089	0.048				
		D1	WT	Long	0.107	0.042		-1.014	0.310	
				Short	0.044	0.029				
		D2	WT	Long	0.214	0.078		-1.532	0.125	
				Short	0.289	0.065				
		D3	WT	Long	0.453	0.130		-1.400	0.161	
				Short	0.622	0.122				
		D4	WT	Long	0.863	0.152		-0.771	0.441	
				Short	0.756	0.104				
		D5	WT	Long	0.829	0.187		-0.770	0.441	
				Short	1.044	0.262				
		D6	WT	Long	0.795	0.113		-0.178	0.859	
				Short	0.778	0.209				
		D7	WT	Long	1.077	0.182		-0.415	0.678	
				Short	1.000	0.149				
		D8	WT	Long	1.350	0.202		-0.840	0.401	
				Short	1.156	0.152				
		D9	WT	Long	1.581	0.329		-1.245	0.213	
				Long	1.244	0.172				
		D10	WT	Short	1.504	0.269		-0.280	0.779	
				Long	1.333	0.170				
		D11	WT	Short	1.701	0.404		-0.593	0.553	
				Long	1.400	0.231				
		D12	WT	Short	1.581	0.190		-1.362	0.173	
				Long	1.244	0.124				
		D13	WT	Short	1.479	0.185		-2.073	0.038	
				Long	1.089	0.075				
		D14	WT	Short	1.667	0.208		-2.310	0.021	*
				Long	0.978	0.122				
		D15	WT	Short	1.598	0.220		-1.836	0.066	
				Long	1.000	0.167				
		D16	WT	Short	1.427	0.181		-1.423	0.155	
				Long	1.111	0.068				

1_6

HE frequency in pre-US + US

Table	Related Figure	Day	Conditon	Group	Mean	SEM	Stastistical test	Z	P	*
Table S1	Figure S2A	D0	Long	KO	0.167	0.055	Mann-Whitney U Test	-1.141	0.297	
				WT	0.292	0.247				
		D1	Long	KO	0.111	0.044		-0.201	0.863	
				WT	0.100	0.040				
		D2	Long	KO	0.292	0.114		-0.419	0.730	
				WT	0.194	0.073				
		D3	Long	KO	0.333	0.125		-1.103	0.297	
				WT	0.556	0.149				
		D4	Long	KO	0.917	0.315		-0.268	0.796	
				WT	0.833	0.144				
		D5	Long	KO	1.056	0.212		-0.178	0.863	
				WT	1.000	0.306				
		D6	Long	KO	1.333	0.351		-1.838	0.077	
				WT	0.528	0.188				
		D7	Long	KO	1.056	0.393		-0.809	0.436	
				WT	0.694	0.246				
		D8	Long	KO	1.500	0.344		-0.889	0.387	
				WT	1.056	0.311				
		D9	Long	KO	1.583	0.460		-0.664	0.546	
				WT	1.500	0.550				
		D10	Long	KO	0.917	0.470		-0.448	0.666	
				WT	1.139	0.384				
		D11	Long	KO	0.833	0.323		-0.535	0.605	
				WT	1.667	0.664				
		D12	Long	KO	0.972	0.539		-0.539	0.605	
				WT	0.889	0.304				
		D13	Long	KO	0.917	0.264		-0.133	0.931	
				WT	0.917	0.309				
		D14	Long	KO	0.611	0.334		-1.232	0.258	
				WT	0.806	0.235				
		D15	Long	KO	0.861	0.328		-0.182	0.863	
				WT	0.889	0.326				
		D16	Long	KO	1.111	0.398		-0.315	0.796	
				WT	0.778	0.258				
Table S1	Figure S2A	D0	Short	KO	0.111	0.033	Mann-Whitney U Test	-0.094	0.931	
				WT	0.167	0.075				
		D1	Short	KO	0.375	0.108		-1.820	0.077	
				WT	0.125	0.042				
		D2	Short	KO	0.403	0.125		-0.453	0.666	
				WT	0.319	0.081				
		D3	Short	KO	0.639	0.157		-0.134	0.931	
				WT	0.694	0.181				
		D4	Short	KO	0.833	0.132		-0.540	0.605	
				WT	0.750	0.156				
		D5	Short	KO	1.167	0.220		-0.178	0.863	
				WT	1.222	0.275				
		D6	Short	KO	1.278	0.255		-1.251	0.222	
				WT	0.806	0.199				
		D7	Short	KO	1.333	0.144		-0.179	0.863	
				WT	1.278	0.206				
		D8	Short	KO	1.944	0.327		-2.087	0.040	*
				WT	1.111	0.151				
		D9	Short	KO	1.444	0.249		-0.357	0.730	
				WT	1.417	0.177				
		D10	Short	KO	1.556	0.311		-0.400	0.730	
				WT	1.472	0.214				
		D11	Short	KO	1.500	0.325		-0.312	0.796	
				WT	1.306	0.242				
		D12	Short	KO	1.333	0.298		-0.359	0.730	
				WT	1.361	0.196				
		D13	Short	KO	1.361	0.221		-1.449	0.161	
				WT	1.139	0.073				
		D14	Short	KO	1.222	0.202		-0.134	0.931	
				WT	1.194	0.220				
		D15	Short	KO	1.028	0.193		-0.403	0.730	
				WT	1.028	0.226				
		D16	Short	KO	1.167	0.161		-0.765	0.489	
				WT	0.972	0.128				

HE frequency in pre-US + US

Table	Related Figure	Day	Group	Condition	Mean	SEM	Stastistical test	Z	P	*
Table S1	Figure S2A	D0	KO	Long	0.167	0.055	Wilcoxon Signed Ranks Test	-0.738	0.461	
				Short	0.111	0.033				
		D1	KO	Long	0.111	0.044		-2.201	0.028	*
				Short	0.375	0.108				
		D2	KO	Long	0.292	0.114		-1.300	0.194	
				Short	0.403	0.125				
		D3	KO	Long	0.333	0.125		-1.420	0.156	
				Short	0.639	0.157				
		D4	KO	Long	0.917	0.315		-0.344	0.731	
				Short	0.833	0.132				
		D5	KO	Long	1.056	0.212		-0.599	0.549	
				Short	1.167	0.220				
		D6	KO	Long	1.333	0.351		0.000	1.000	
				Short	1.278	0.255				
		D7	KO	Long	1.056	0.393		-1.129	0.259	
				Short	1.333	0.144				
		D8	KO	Long	1.500	0.344		-1.476	0.140	
				Short	1.944	0.327				
		D9	KO	Long	1.583	0.460		-0.254	0.799	
				Long	1.444	0.249				
		D10	KO	Short	0.917	0.470		-1.402	0.161	
				Long	1.556	0.311				
		D11	KO	Short	0.833	0.323		-1.666	0.096	
				Long	1.500	0.325				
		D12	KO	Short	0.972	0.539		-1.424	0.154	
				Long	1.333	0.298				
		D13	KO	Short	0.917	0.264		-1.371	0.170	
				Long	1.361	0.221				
		D14	KO	Short	0.611	0.334		-1.602	0.109	
				Long	1.222	0.202				
		D15	KO	Short	0.861	0.328		-0.357	0.721	
				Long	1.028	0.193				
		D16	KO	Short	1.111	0.398		0.000	1.000	
				Long	1.167	0.161				
Table S1	Figure S2A	D0	WT	Long	0.292	0.247	Wilcoxon Signed Ranks Test	-0.271	0.786	
				Short	0.167	0.075				
		D1	WT	Long	0.097	0.040		-0.552	0.581	
				Short	0.125	0.042				
		D2	WT	Long	0.194	0.073		-1.289	0.197	
				Short	0.319	0.081				
		D3	WT	Long	0.556	0.149		-1.394	0.163	
				Short	0.694	0.181				
		D4	WT	Long	0.833	0.144		-0.285	0.776	
				Short	0.750	0.156				
		D5	WT	Long	1.000	0.306		-1.205	0.228	
				Short	1.222	0.275				
		D6	WT	Long	0.528	0.188		-0.775	0.438	
				Short	0.806	0.199				
		D7	WT	Long	0.694	0.246		-2.254	0.024	*
				Short	1.278	0.206				
		D8	WT	Long	1.056	0.311		-0.085	0.932	
				Short	1.111	0.151				
		D9	WT	Long	1.500	0.550		.000d	1.000	
				Long	1.417	0.177				
		D10	WT	Short	1.139	0.384		-0.655	0.512	
				Long	1.472	0.214				
		D11	WT	Short	1.667	0.664		-0.356	0.722	
				Long	1.306	0.242				
		D12	WT	Short	0.889	0.304		-1.191	0.234	
				Long	1.361	0.196				
		D13	WT	Short	0.917	0.309		-0.775	0.438	
				Long	1.139	0.073				
		D14	WT	Short	0.806	0.235		-1.544	0.123	
				Long	1.194	0.220				
		D15	WT	Short	0.889	0.326		-0.535	0.592	
				Long	1.028	0.226				
		D16	WT	Short	0.778	0.258		-0.846	0.397	
				Long	0.972	0.128				

1_7

HE frequency in pre-CS										
Table	Related Figure	Day	Conditon	Group	Mean	SEM	Stastistical test	Z	P	*
Table S1	Figure S2A	D0	Long	KO	0.369	0.046	Mann-Whitney U Test	-2.121	0.031	*
				WT	0.217	0.088				
		D1	Long	KO	0.513	0.084		-2.123	0.031	*
				WT	0.233	0.054				
		D2	Long	KO	0.669	0.101		-2.255	0.024	*
				WT	0.376	0.04				
		D3	Long	KO	0.541	0.098		-0.708	0.489	
				WT	0.637	0.08				
		D4	Long	KO	0.737	0.101		-0.530	0.605	
				WT	0.807	0.123				
		D5	Long	KO	0.67	0.098		-0.886	0.387	
				WT	0.856	0.091				
		D6	Long	KO	0.941	0.157		-1.724	0.094	
				WT	0.585	0.096				
		D7	Long	KO	0.778	0.165		-1.106	0.297	
				WT	0.856	0.091				
		D8	Long	KO	0.693	0.151		-1.370	0.190	
				WT	0.904	0.108				
		D9	Long	KO	0.693	0.113		-0.133	0.931	
				WT	0.622	0.087				
		D10	Long	KO	0.437	0.067		-1.901	0.063	
				WT	0.704	0.088				
		D11	Long	KO	0.522	0.07		-1.510	0.136	
				WT	0.667	0.064				
		D12	Long	KO	0.5	0.052		-1.461	0.161	
				WT	0.707	0.114				
		D13	Long	KO	0.478	0.104		-0.887	0.387	
				WT	0.559	0.073				
		D14	Long	KO	0.615	0.084		-0.133	0.931	
				WT	0.633	0.096				
		D15	Long	KO	0.556	0.058		-1.105	0.297	
				WT	0.652	0.088				
		D16	Long	KO	0.556	0.078		-0.442	0.666	
				WT	0.596	0.071				
Table S1	Figure S2A	D0	Short	KO	0.193	0.051	Mann-Whitney U Test	-1.245	0.222	
				WT	0.119	0.035				
		D1	Short	KO	0.335	0.068		-1.336	0.190	
				WT	0.237	0.037				
		D2	Short	KO	0.38	0.049		-2.435	0.014	*
				WT	0.213	0.032				
		D3	Short	KO	0.478	0.097		-0.133	0.931	
				WT	0.515	0.1				
		D4	Short	KO	0.596	0.077		-0.044	1.000	
				WT	0.615	0.074				
		D5	Short	KO	0.444	0.072		-0.620	0.546	
				WT	0.519	0.064				
		D6	Short	KO	0.859	0.142		-1.460	0.161	
				WT	0.541	0.098				
		D7	Short	KO	0.648	0.086		-0.884	0.387	
				WT	0.781	0.125				
		D8	Short	KO	0.859	0.161		-1.150	0.258	
				WT	0.611	0.098				
		D9	Short	KO	0.763	0.205		-0.354	0.730	
				WT	0.781	0.159				
		D10	Short	KO	0.53	0.067		-0.444	0.666	
				WT	0.585	0.16				
		D11	Short	KO	0.533	0.056		-0.265	0.796	
				WT	0.633	0.173				
		D12	Short	KO	0.574	0.102		-0.620	0.546	
				WT	0.456	0.084				
		D13	Short	KO	0.526	0.113		-1.061	0.297	
				WT	0.348	0.061				
		D14	Short	KO	0.485	0.089		-1.336	0.190	
				WT	0.315	0.062				
		D15	Short	KO	0.307	0.083		-0.709	0.489	
				WT	0.396	0.085				
		D16	Short	KO	0.407	0.056		-1.863	0.063	
				WT	0.27	0.045				

HE frequency in post-CS										
Table	Related Figure	Day	Conditon	Group	Mean	SEM	Stastistical test	Z	P	*
Table S1	Figure S2A	D0	Long	KO	0.219	0.038	Mann-Whitney U Test	-1.552	0.121	
				WT	0.167	0.066				
		D1	Long	KO	0.283	0.052		-0.620	0.535	
				WT	0.319	0.116				
		D2	Long	KO	0.320	0.070		-0.753	0.451	
				WT	0.374	0.046				
		D3	Long	KO	0.515	0.110		-0.712	0.476	
				WT	0.648	0.097				
		D4	Long	KO	0.352	0.082		-0.355	0.722	
				WT	0.400	0.083				
		D5	Long	KO	0.344	0.056		-0.089	0.929	
				WT	0.330	0.057				
		D6	Long	KO	0.267	0.065		-0.222	0.825	
				WT	0.393	0.118				
		D7	Long	KO	0.285	0.081		-0.177	0.859	
				WT	0.267	0.084				
		D8	Long	KO	0.204	0.063		-0.045	0.964	
				WT	0.211	0.044				
		D9	Long	KO	0.211	0.084		-0.178	0.859	
				WT	0.204	0.064				
		D10	Long	KO	0.263	0.124		0.000	1.000	
				WT	0.130	0.044				
		D11	Long	KO	0.259	0.104		-0.672	0.502	
				WT	0.141	0.052				
		D12	Long	KO	0.178	0.097		-0.673	0.501	
				WT	0.126	0.047				
		D13	Long	KO	0.189	0.086		-1.243	0.214	
				WT	0.256	0.098				
		D14	Long	KO	0.215	0.094		-0.755	0.450	
				WT	0.181	0.030				
		D15	Long	KO	0.185	0.084		-1.064	0.287	
				WT	0.230	0.034				
		D16	Long	KO	0.178	0.086		-0.582	0.561	
				WT	0.200	0.092				
Table S1	Figure S2A	D0	Short	KO	0.200	0.050	Mann-Whitney U Test	-1.994	0.046	*
				WT	0.100	0.030				
		D1	Short	KO	0.352	0.083		-1.283	0.199	
				WT	0.198	0.051				
		D2	Short	KO	0.430	0.056		-2.087	0.037	*
				WT	0.291	0.043				
		D3	Short	KO	0.526	0.097		-0.577	0.564	
				WT	0.607	0.136				
		D4	Short	KO	0.770	0.092		-0.531	0.595	
				WT	0.741	0.100				
		D5	Short	KO	0.552	0.057		-1.813	0.070	
				WT	0.804	0.115				
		D6	Short	KO	0.944	0.165		-1.769	0.077	
				WT	0.578	0.098				
		D7	Short	KO	0.800	0.111		-0.221	0.825	
				WT	0.793	0.121				
		D8	Short	KO	0.911	0.170		-0.265	0.791	
				WT	0.881	0.124				
		D9	Short	KO	1.015	0.289		-0.398	0.690	
				WT	0.733	0.129				
		D10	Short	KO	0.630	0.127		-0.310	0.757	
				WT	0.670	0.127				
		D11	Short	KO	0.596	0.128		-0.532	0.595	
				WT	0.733	0.235				
		D12	Short	KO	0.574	0.129		-0.133	0.894	
				WT	0.559	0.103				
		D13	Short	KO	0.489	0.116		-0.265	0.791	
				WT	0.404	0.067				
		D14	Short	KO	0.496	0.072		-0.487	0.626	
				WT	0.430	0.066				
		D15	Short	KO	0.433	0.098		-0.266	0.790	
				WT	0.374	0.086				
		D16	Short	KO	0.385	0.084		-0.133	0.894	
				WT	0.400	0.067				

1_8

HE frequency in D0 and D1									
Table	Related Figure	Condition/Group		Mean	SEM	Stastistical test	Z	P	*
Table S1	Figure 1E	D0	KO	6.361	1.354	Mann-Whitney U Test	-2.471	0.013	*
			WT	3.444	0.919				
		D1	KO	7.944	1.354		-2.078	0.037	*
			WT	4.250	0.646				
		KO	D0	6.361	1.052	Wilcoxon Signed Ranks Test	-1.090	0.276	
			D1	7.944	1.354				
		WT	D0	3.444	0.919		-1.091	0.275	
			D1	4.250	0.646				

2_1

Hit and correct rejection rates (CS)

Table	Related Figure	Day	Condition	Group	Mean	SEM	Stastistical test	Z	P	*
Table S2	Figure 1F	D0	Hit	KO	8.89	3.51	Mann-Whitney U Test	-2.240	0.031	*
				WT	32.22	9.83				
		D1	Hit	KO	16.67	6.24		-0.460	0.666	
				WT	21.11	8.24				
		D2	Hit	KO	26.67	8.16		-0.315	0.796	
				WT	37.78	9.97				
		D3	Hit	KO	35.56	9.44		-1.126	0.297	
				WT	44.44	10.56				
		D4	Hit	KO	64.44	8.99		-0.045	1.000	
				WT	60.00	12.91				
		D5	Hit	KO	63.33	10.27		-0.314	0.796	
				WT	63.33	9.86				
		D6	Hit	KO	70.00	8.66		-1.027	0.340	
				WT	81.11	7.90				
		D7	Hit	KO	78.89	6.55		-0.268	0.796	
				WT	74.44	10.29				
		D8	Hit	KO	86.67	2.89		-1.697	0.113	
				WT	87.78	7.95				
		D9	Hit	KO	86.67	4.41		-1.855	0.077	
				WT	93.33	5.53				
		D10	Hit	KO	88.89	4.23		-0.417	0.730	
				WT	85.56	10.42				
		D11	Hit	KO	94.44	2.42		-0.722	0.489	
				WT	75.56	11.07				
		D12	Hit	KO	94.44	2.42		-0.419	0.730	
				WT	88.89	7.72				
		D13	Hit	KO	90.00	3.73		-0.904	0.387	
				WT	82.22	6.62				
		D14	Hit	KO	92.22	2.22		-0.637	0.546	
				WT	87.78	6.41				
		D15	Hit	KO	94.44	2.42		-0.233	0.863	
				WT	88.89	6.96				
		D16	Hit	KO	93.33	3.33		-0.472	0.666	
				WT	94.44	3.77				
Table S2	Figure 1F	D0	Correctr ejecton	KO	88.89	3.09	Mann-Whitney U Test	-1.156	0.436	
				WT	95.56	2.42				
		D1	Correctr ejecton	KO	78.89	5.64		-2.535	0.050	
				WT	97.78	1.47				
		D2	Correctr ejecton	KO	73.33	6.45		-1.594	0.161	
				WT	82.22	4.01				
		D3	Correctr ejecton	KO	72.22	6.83		-0.140	0.931	
				WT	77.78	4.34				
		D4	Correctr ejecton	KO	57.78	6.83		-0.496	0.666	
				WT	65.56	3.77				
		D5	Correctr ejecton	KO	55.56	8.35		-0.331	0.796	
				WT	61.11	6.11				
		D6	Correctr ejecton	KO	47.78	7.41		-1.676	0.113	
				WT	65.56	8.84				
		D7	Correctr ejecton	KO	41.11	9.20		-1.606	0.161	
				WT	55.56	7.09				
		D8	Correctr ejecton	KO	37.78	7.78		-0.225	0.863	
				WT	51.11	6.33				
		D9	Correctr ejecton	KO	36.67	7.82		-2.553	0.011	*
				WT	51.11	5.12				
		D10	Correctr ejecton	KO	41.11	9.20		-1.147	0.297	
				WT	43.33	8.16				
		D11	Correctr ejecton	KO	42.22	9.09		-0.451	0.666	
				WT	46.67	7.07				
		D12	Correctr ejecton	KO	36.67	10.14		-0.814	0.436	
				WT	48.89	4.55				
		D13	Correctr ejecton	KO	37.78	7.60		-1.034	0.340	
				WT	51.11	3.09				
		D14	Correctr ejecton	KO	33.33	7.99		-1.793	0.077	
				WT	61.11	4.23				
		D15	Correctr ejecton	KO	43.33	10.93		-0.989	0.340	
				WT	55.56	6.48				
		D16	Correctr ejecton	KO	35.56	7.09		-0.637	0.546	
				WT	50.00	2.89				

2_2

Hit and correct rejection rates (CS + pre-US)

Table	Related Figure	Day	Condition	Group	Mean	SEM	Stastistical test	Z	P	*
Table S2	Figure S2B	D0	Hit	KO	27.78	8.78	Mann-Whitney U Test	-2.176	0.031	*
				WT	6.67	2.36				
		D1	Hit	KO	18.89	7.72		-0.273	0.796	
				WT	13.33	5.27				
		D2	Hit	KO	32.22	9.83		-0.762	0.489	
				WT	24.44	8.52				
		D3	Hit	KO	38.89	10.06		-0.584	0.605	
				WT	24.44	7.09				
		D4	Hit	KO	56.67	12.02		-0.179	0.863	
				WT	56.67	10.27				
		D5	Hit	KO	50.00	9.13		-0.090	0.931	
				WT	54.44	11.32				
		D6	Hit	KO	75.56	7.66		-0.860	0.436	
				WT	62.22	9.09				
		D7	Hit	KO	68.89	11.48		-0.091	0.931	
				WT	71.11	6.33				
		D8	Hit	KO	87.78	7.95		-1.532	0.161	
				WT	81.11	4.84				
		D9	Hit	KO	87.78	8.62		-1.656	0.136	
				WT	78.89	6.33				
		D10	Hit	KO	80.00	10.41		-1.220	0.297	
				WT	83.33	5.77				
		D11	Hit	KO	70.00	11.55		-1.139	0.297	
				WT	84.44	5.56				
		D12	Hit	KO	83.33	9.13		-0.152	0.931	
				WT	86.67	4.71				
		D13	Hit	KO	77.78	7.03		-0.781	0.489	
				WT	86.67	4.71				
		D14	Hit	KO	83.33	7.64		-0.048	1.000	
				WT	84.44	3.77				
		D15	Hit	KO	82.22	9.25		-0.101	0.931	
				WT	87.78	4.01				
		D16	Hit	KO	87.78	4.94		-0.686	0.605	
				WT	92.22	3.24				
Table S2	Figure S2B	D0	Correctr ejecton	KO	95.56	2.42	Mann-Whitney U Test	-1.719	0.113	
				WT	98.89	1.11				
		D1	Correctr ejecton	KO	93.33	2.36		-2.616	0.014	*
				WT	100.00	0.00				
		D2	Correctr ejecton	KO	88.89	2.61		-1.134	0.297	
				WT	94.44	1.76				
		D3	Correctr ejecton	KO	88.89	3.51		-0.762	0.489	
				WT	88.89	2.61				
		D4	Correctr ejecton	KO	78.89	6.11		-1.269	0.222	
				WT	77.78	4.34				
		D5	Correctr ejecton	KO	84.44	3.38		-0.670	0.546	
				WT	83.33	2.36				
		D6	Correctr ejecton	KO	81.11	4.23		-1.604	0.113	
				WT	90.00	4.08				
		D7	Correctr ejecton	KO	70.00	7.07		-0.936	0.387	
				WT	86.67	1.67				
		D8	Correctr ejecton	KO	77.78	5.47		-1.561	0.136	
				WT	78.89	4.55				
		D9	Correctr ejecton	KO	71.11	4.23		-1.914	0.063	
				WT	86.67	2.89				
		D10	Correctr ejecton	KO	73.33	5.00		-0.536	0.605	
				WT	82.22	4.94				
		D11	Correctr ejecton	KO	76.67	7.64		-0.534	0.605	
				WT	77.78	3.64				
		D12	Correctr ejecton	KO	71.11	6.55		-1.123	0.297	
				WT	78.89	4.84				
		D13	Correctr ejecton	KO	73.33	8.16		-1.350	0.190	
				WT	85.56	2.94				
		D14	Correctr ejecton	KO	63.33	7.99		-2.377	0.019	*
				WT	81.11	4.55				
		D15	Correctr ejecton	KO	67.78	8.46		-1.249	0.222	
				WT	81.11	4.84				
		D16	Correctr ejecton	KO	70.00	7.26		-2.163	0.031	*
				WT	77.78	3.64				

2_3

Hi ratet vs. Correct rejection rate (CS)

Table	Related Figure	Day	Group	Condition	Mean	SEM	Stastistical test	Z	P	*
Table S3	Figure S2C	D0	KO	Long	6.67	2.36	Wilcoxon Signed Ranks Test	-2.682	0.007	**
				Short	98.89	1.11				
		D1	KO	Long	13.33	5.27		-2.673	0.008	**
				Short	100.00	0.00				
		D2	KO	Long	24.44	8.52		-2.673	0.008	**
				Short	94.44	1.76				
		D3	KO	Long	24.44	7.09		-2.492	0.013	*
				Short	88.89	2.61				
		D4	KO	Long	56.67	10.27		-1.266	0.205	
				Short	77.78	4.34				
		D5	KO	Long	54.44	11.32		-2.320	0.020	*
				Short	83.33	2.36				
		D6	KO	Long	62.22	9.09		-0.060	0.952	
				Short	90.00	4.08				
		D7	KO	Long	71.11	6.33		-0.281	0.779	
				Short	86.67	1.67				
		D8	KO	Long	81.11	4.84		-1.194	0.233	
				Short	78.89	4.55				
		D9	KO	Long	78.89	6.33		-1.608	0.108	
				Long	86.67	2.89				
		D10	KO	Short	83.33	5.77		-0.718	0.473	
				Long	82.22	4.94				
		D11	KO	Short	84.44	5.56		-0.280	0.779	
				Long	77.78	3.64				
		D12	KO	Short	86.67	4.71		-1.130	0.258	
				Long	78.89	4.84				
		D13	KO	Short	86.67	4.71		-0.417	0.677	
				Long	85.56	2.94				
		D14	KO	Short	84.44	3.77		-1.196	0.232	
				Long	81.11	4.55				
		D15	KO	Short	87.78	4.01		-0.893	0.372	
				Long	81.11	4.84				
		D16	KO	Short	92.22	3.24		-1.411	0.158	
				Long	77.78	3.64				
Table S3	Figure S2C	D0	WT	Long	27.78	8.78	Wilcoxon Signed Ranks Test	-2.701	0.007	**
				Short	95.56	2.42				
		D1	WT	Long	18.89	7.72		-2.701	0.007	**
				Short	93.33	2.36				
		D2	WT	Long	32.22	9.83		-2.694	0.007	**
				Short	88.89	2.61				
		D3	WT	Long	38.89	10.06		-2.670	0.008	**
				Short	88.89	3.51				
		D4	WT	Long	56.67	12.02		-1.436	0.151	
				Short	78.89	6.11				
		D5	WT	Long	50.00	9.13		-1.796	0.072	
				Short	84.44	3.38				
		D6	WT	Long	75.56	7.66		-2.066	0.039	*
				Short	81.11	4.23				
		D7	WT	Long	68.89	11.48		-1.904	0.057	
				Short	70.00	7.07				
		D8	WT	Long	87.78	7.95		-0.541	0.589	
				Short	77.78	5.47				
		D9	WT	Long	87.78	8.62		-0.954	0.340	
				Long	71.11	4.23				
		D10	WT	Short	80.00	10.41		-0.179	0.858	
				Long	73.33	5.00				
		D11	WT	Short	70.00	11.55		-1.029	0.303	
				Long	76.67	7.64				
		D12	WT	Short	83.33	9.13		-0.846	0.397	
				Long	71.11	6.55				
		D13	WT	Short	77.78	7.03		-0.085	0.932	
				Long	73.33	8.16				
		D14	WT	Short	83.33	7.64		-0.426	0.670	
				Long	63.33	7.99				
		D15	WT	Short	82.22	9.25		-0.877	0.380	
				Long	67.78	8.46				
		D16	WT	Short	87.78	4.94		-2.200	0.028	*
				Long	70.00	7.26				

Supplementary Table 2. HE accuracy. Differences between groups and conditions in hit and correct rejection rates, as well as between hit and correct rejection rates, were analyzed separately using the Mann–Whitney U test and Wilcoxon signed-rank test. *p < 0.05, **p < 0.01 (N = 9). Abbreviations: D, training day; KO, CRBN KO mice; WT, wild-type mice.

3_1

Table	Related Figure	Day	Condition	Group	Mean	SEM	Stastistical test	Z	P	*
HE peak frequency										
Table S3	Figure 1G	D0	Long	KO	1.220	0.220	Mann-Whitney U Test	-1.748	0.113	
				WT	0.780	0.430				
		D1	Long	KO	0.780	0.220		-0.458	0.730	
				WT	1.000	0.290				
		D2	Long	KO	1.220	0.280		-0.549	0.605	
				WT	1.670	0.470				
		D3	Long	KO	2.220	0.490		-0.276	0.796	
				WT	2.110	0.350				
		D4	Long	KO	2.780	0.620		-0.545	0.605	
				WT	2.440	0.290				
		D5	Long	KO	2.890	0.420		-0.182	0.863	
				WT	3.000	0.530				
		D6	Long	KO	4.110	0.420		-1.878	0.077	
				WT	3.220	0.280				
		D7	Long	KO	3.890	0.680		-0.586	0.605	
				WT	3.330	0.410				
		D8	Long	KO	5.220	0.680		-2.312	0.024	*
				WT	3.560	0.340				
		D9	Long	KO	5.220	0.600		-1.162	0.258	
				WT	4.220	0.660				
		D10	Long	KO	5.110	0.610		-1.691	0.113	
				WT	3.890	0.260				
		D11	Long	KO	5.110	0.960		-0.179	0.863	
				WT	4.440	0.560				
		D12	Long	KO	4.440	0.650		-0.091	0.931	
				WT	4.670	0.580				
		D13	Long	KO	5.330	0.670		-1.305	0.222	
				WT	4.330	0.370				
		D14	Long	KO	4.220	0.400		-0.091	0.931	
				WT	4.330	0.410				
		D15	Long	KO	4.670	0.470		-0.273	0.796	
				WT	4.440	0.380				
		D16	Long	KO	4.670	0.470		-0.872	0.436	
				WT	5.000	0.410				
Table S3	Figure 1G	D0	Short	KO	1.110	0.200	Mann-Whitney U Test	-0.191	0.863	
				WT	1.110	0.350				
		D1	Short	KO	1.890	0.590		-1.189	0.297	
				WT	1.000	0.240				
		D2	Short	KO	2.110	0.310		-2.100	0.050	
				WT	1.220	0.220				
		D3	Short	KO	2.560	0.440		-0.047	1.000	
				WT	2.670	0.500				
		D4	Short	KO	2.890	0.590		-0.182	0.863	
				WT	2.890	0.560				
		D5	Short	KO	2.670	0.240		-0.599	0.605	
				WT	3.220	0.550				
		D6	Short	KO	3.440	0.530		-1.582	0.136	
				WT	2.330	0.370				
		D7	Short	KO	3.560	0.440		-0.731	0.489	
				WT	3.110	0.350				
		D8	Short	KO	4.670	0.620		-1.906	0.063	
				WT	3.000	0.290				
		D9	Short	KO	3.670	0.620		-0.275	0.796	
				WT	3.440	0.500				
		D10	Short	KO	3.220	0.280		-0.095	0.931	
				WT	3.330	0.290				
		D11	Short	KO	3.780	0.360		-0.911	0.387	
				WT	3.330	0.470				
		D12	Short	KO	3.780	0.550		-0.136	0.931	
				WT	3.780	0.360				
		D13	Short	KO	3.560	0.470		-1.780	0.094	
				WT	2.440	0.180				
		D14	Short	KO	4.220	0.550		-1.499	0.161	
				WT	3.110	0.260				
		D15	Short	KO	3.440	0.650		-0.592	0.605	
				WT	3.440	0.440				
		D16	Short	KO	3.440	0.560		-0.651	0.546	
				WT	2.890	0.200				

Table	Related Figure	Day	Group	Condition	Mean	SEM	Stastistical test	Z	P	*
HE peak frequency										
Table S3	Figure 1G	D0	KO	Long	1.220	0.220	Wilcoxon Signed Ranks Test	-0.447	0.655	
				Short	1.110	0.200				
		D1	KO	Long	0.780	0.220		-1.633	0.102	
				Short	1.890	0.590				
		D2	KO	Long	1.220	0.280		-2.271	0.023	*
				Short	2.110	0.310				
		D3	KO	Long	2.220	0.490		-0.707	0.480	
				Short	2.560	0.440				
		D4	KO	Long	2.780	0.620		-0.256	0.798	
				Short	2.890	0.590				
		D5	KO	Long	2.890	0.420		-0.649	0.516	
				Short	2.670	0.240				
		D6	KO	Long	4.110	0.420		-1.268	0.205	
				Short	3.440	0.530				
		D7	KO	Long	3.890	0.680		-1.000	0.317	
				Short	3.560	0.440				
		D8	KO	Long	5.220	0.680		-1.098	0.272	
				Short	4.670	0.620				
		D9	KO	Long	5.220	0.600		-2.354	0.019	*
				Long	3.670	0.620				
		D10	KO	Short	5.110	0.610		-2.379	0.017	*
				Long	3.220	0.280				
		D11	KO	Short	5.110	0.960		-1.538	0.124	
				Long	3.780	0.360				
		D12	KO	Short	4.440	0.650		-1.137	0.256	
				Long	3.780	0.550				
		D13	KO	Short	5.330	0.670		-2.129	0.033	*
				Long	3.560	0.470				
		D14	KO	Short	4.220	0.400		-0.333	0.739	
				Long	4.220	0.550				
		D15	KO	Short	4.670	0.470		-1.430	0.153	
				Long	3.440	0.650				
		D16	KO	Short	4.670	0.470		-1.612	0.107	
				Long	3.440	0.560				
Table S3	Figure 1G	D0	WT	Long	0.780	0.430	Wilcoxon Signed Ranks Test	-0.750	0.453	
				Short	1.110	0.350				
		D1	WT	Long	1.000	0.290		0.000	1.000	
				Short	1.000	0.240				
		D2	WT	Long	1.670	0.470		-1.300	0.194	
				Short	1.220	0.220				
		D3	WT	Long	2.110	0.350		-1.414	0.157	
				Short	2.670	0.500				
		D4	WT	Long	2.440	0.290		-0.707	0.480	
				Short	2.890	0.560				
		D5	WT	Long	3.000	0.530		-0.531	0.595	
				Short	3.220	0.550				
		D6	WT	Long	3.220	0.280		-1.480	0.139	
				Short	2.330	0.370				
		D7	WT	Long	3.330	0.410		-0.702	0.483	
				Short	3.110	0.350				
		D8	WT	Long	3.560	0.340		-1.406	0.160	
				Short	3.000	0.290				
		D9	WT	Long	4.220	0.660		-1.311	0.190	
				Long	3.440	0.500				
		D10	WT	Short	3.890	0.260		-1.155	0.248	
				Long	3.330	0.290				
		D11	WT	Short	4.440	0.560		-1.638	0.101	
				Long	3.330	0.470				
		D12	WT	Short	4.670	0.580		-1.121	0.262	
				Long	3.780	0.360				
		D13	WT	Short	4.330	0.370		-2.555	0.011	*
				Long	2.440	0.180				
		D14	WT	Short	4.330	0.410		-2.050	0.040	*
				Long	3.110	0.260				
		D15	WT	Short	4.440	0.380		-2.124	0.034	*
				Long	3.440	0.440				
		D16	WT	Short	5.000	0.410		-2.539	0.011	*
				Long	2.890	0.200				

3_2

Table	Related Figure	Day	Condition	Group	Mean	SEM	Stastistical test	Z	P	*
HE peak latency										
Table S3	Figure 1G	D0	Long	KO	4.000	1.093	Mann-Whitney U Test	-2.037	0.050	
				WT	1.778	1.222				
		D1	Long	KO	1.000	0.408		-0.252	0.863	
				WT	2.000	1.443				
		D2	Long	KO	4.222	1.310		-0.911	0.387	
				WT	3.111	1.438				
		D3	Long	KO	3.667	1.291		-0.542	0.605	
				WT	5.111	1.728				
		D4	Long	KO	2.889	1.296		-0.740	0.489	
				WT	2.111	1.111				
		D5	Long	KO	4.667	1.202		-0.267	0.796	
				WT	5.444	1.608				
		D6	Long	KO	4.222	1.164		-0.311	0.796	
				WT	5.111	1.476				
		D7	Long	KO	3.556	1.215		-1.211	0.258	
				WT	4.222	0.572				
		D8	Long	KO	3.222	1.382		-0.046	1.000	
				WT	3.222	1.278				
		D9	Long	KO	5.000	1.333		-0.489	0.666	
				WT	4.000	1.179				
		D10	Long	KO	5.111	1.172		-1.203	0.258	
				WT	3.111	1.060				
		D11	Long	KO	5.556	1.600		-0.045	1.000	
				WT	5.556	1.365				
		D12	Long	KO	3.111	1.252		-0.271	0.796	
				WT	3.778	1.245				
		D13	Long	KO	5.111	1.348		-1.069	0.297	
				WT	2.889	0.633				
		D14	Long	KO	3.889	0.889		-0.446	0.666	
				WT	4.333	0.833				
		D15	Long	KO	5.222	1.579		-0.403	0.730	
				WT	4.333	0.667				
		D16	Long	KO	5.000	1.414		-0.488	0.666	
				WT	5.778	1.103				
Table S3	Figure 1G	D0	Short	KO	3.667	1.312	Mann-Whitney U Test	0.000	1.000	
				WT	4.000	1.616				
		D1	Short	KO	3.333	1.312		-0.456	0.666	
				WT	4.333	1.258				
		D2	Short	KO	6.222	1.289		-2.051	0.050	
				WT	1.889	1.020				
		D3	Short	KO	5.667	1.607		-1.081	0.297	
				WT	3.556	1.692				
		D4	Short	KO	3.444	1.271		-0.498	0.666	
				WT	2.333	1.202				
		D5	Short	KO	2.667	0.645		-0.045	1.000	
				WT	2.667	0.816				
		D6	Short	KO	2.778	0.741		-1.161	0.297	
				WT	1.778	0.969				
		D7	Short	KO	2.778	1.392		-0.273	0.796	
				WT	4.111	1.567				
		D8	Short	KO	3.333	1.067		-0.357	0.730	
				WT	4.444	1.271				
		D9	Short	KO	2.556	0.801		-1.832	0.077	
				WT	3.889	0.484				
		D10	Short	KO	2.222	0.683		-0.555	0.605	
				WT	4.000	1.404				
		D11	Short	KO	4.000	1.190		0.000	1.000	
				WT	3.889	1.047				
		D12	Short	KO	2.111	0.772		-1.124	0.297	
				WT	3.222	0.862				
		D13	Short	KO	2.556	0.899		-0.270	0.796	
				WT	2.222	0.521				
		D14	Short	KO	1.667	0.408		-1.399	0.190	
				WT	3.778	1.064				
		D15	Short	KO	1.333	0.577		-2.152	0.031	*
				WT	3.333	0.726				
		D16	Short	KO	1.444	0.377		-0.907	0.387	
				WT	2.000	0.471				

Table	Related Figure	Day	Group	Condition	Mean	SEM	Stastistical test	Z	P	*
HE peak latency										
Table S3	Figure 1G	D0	KO	Long	4.000	1.093	Wilcoxon Signed Ranks Test	-0.140	0.889	
				Short	3.667	1.312				
		D1	KO	Long	1.000	0.408		-1.706	0.088	
				Short	3.333	1.312				
		D2	KO	Long	4.222	1.310		-1.067	0.286	
				Short	6.222	1.289				
		D3	KO	Long	3.667	1.291		-0.844	0.398	
				Short	5.667	1.607				
		D4	KO	Long	2.889	1.296		-0.170	0.865	
				Short	3.444	1.271				
		D5	KO	Long	4.667	1.202		-1.077	0.282	
				Short	2.667	0.645				
		D6	KO	Long	4.222	1.164		-0.845	0.398	
				Short	2.778	0.741				
		D7	KO	Long	3.556	1.215		-0.702	0.483	
				Short	2.778	1.392				
		D8	KO	Long	3.222	1.382		-0.354	0.723	
				Short	3.333	1.067				
		D9	KO	Long	5.000	1.333		-2.252	0.024	*
				Long	2.556	0.801				
		D10	KO	Short	5.111	1.172		-2.184	0.029	*
				Long	2.222	0.683				
		D11	KO	Short	5.556	1.600		-1.268	0.205	
				Long	4.000	1.190				
		D12	KO	Short	3.111	1.252		-1.051	0.293	
				Long	2.111	0.772				
		D13	KO	Short	5.111	1.348		-2.393	0.017	*
				Long	2.556	0.899				
		D14	KO	Short	3.889	0.889		-2.113	0.035	*
				Long	1.667	0.408				
		D15	KO	Short	5.222	1.579		-2.371	0.018	*
				Long	1.333	0.577				
		D16	KO	Short	5.000	1.414		-2.117	0.034	*
				Long	1.444	0.377				
Table S3	Figure 1G	D0	WT	Long	1.778	1.222	Wilcoxon Signed Ranks Test	-1.153	0.249	
				Short	4.000	1.616				
		D1	WT	Long	2.000	1.443		-1.781	0.075	
				Short	4.333	1.258				
		D2	WT	Long	3.111	1.438		-0.315	0.752	
				Short	1.889	1.020				
		D3	WT	Long	5.111	1.728		-0.845	0.398	
				Short	3.556	1.692				
		D4	WT	Long	2.111	1.111		0.000	1.000	
				Short	2.333	1.202				
		D5	WT	Long	5.444	1.608		-1.187	0.235	
				Short	2.667	0.816				
		D6	WT	Long	5.111	1.476		-1.886	0.059	
				Short	1.778	0.969				
		D7	WT	Long	4.222	0.572		-0.070	0.944	
				Short	4.111	1.567				
		D8	WT	Long	3.222	1.278		-0.943	0.345	
				Short	4.444	1.271				
		D9	WT	Long	4.000	1.179		-0.060	0.952	
				Long	3.889	0.484				
		D10	WT	Short	3.111	1.060		-0.211	0.833	
				Long	4.000	1.404				
		D11	WT	Short	5.556	1.365		-0.953	0.341	
				Long	3.889	1.047				
		D12	WT	Short	3.778	1.245		-0.512	0.609	
				Long	3.222	0.862				
		D13	WT	Short	2.889	0.633		-0.991	0.322	
				Long	2.222	0.521				
		D14	WT	Short	4.333	0.833		-0.418	0.676	
				Long	3.778	1.064				
		D15	WT	Short	4.333	0.667		-1.549	0.121	
				Long	3.333	0.726				
		D16	WT	Short	5.778	1.103		-2.524	0.012	*
				Long	2.000	0.471				

Supplementary Table 3. HE and FHE peaks. Differences between groups and conditions in HE and FHE peaks, as well as between HE and FHE peaks, were analyzed separately using the Mann–Whitney U test and Wilcoxon signed-rank test. *p < 0.05, **p < 0.01, ***p < 0.001 (N = 9). Abbreviations: HE, head entry; FHE, fastest HE; D, training day; KO, CRBN KO mice; WT, wild-type mice.

3_3

FHE peak frequency

Table	Related Figure	Day	Condition	Group	Mean	SEM	Stastistical test	Z	P	*
Table S3	Figure S2D	D0	Long	KO	1.444	0.338	Mann-Whitney U Test	-1.720	0.113	
				WT	0.667	0.236				
		D1	Long	KO	0.778	0.222		0.000	1.000	
				WT	0.778	0.222				
		D2	Long	KO	1.222	0.278		-0.046	1.000	
				WT	1.333	0.373				
		D3	Long	KO	1.778	0.434		-1.609	0.136	
				WT	0.889	0.261				
		D4	Long	KO	1.889	0.389		-0.047	1.000	
				WT	1.889	0.309				
		D5	Long	KO	1.778	0.324		-0.752	0.489	
				WT	2.111	0.351				
		D6	Long	KO	2.667	0.333		-0.537	0.666	
				WT	2.444	0.338				
		D7	Long	KO	2.667	0.333		-0.691	0.546	
				WT	2.333	0.333				
		D8	Long	KO	2.778	0.401		-1.178	0.297	
				WT	2.222	0.147				
		D9	Long	KO	3.111	0.389		-0.726	0.489	
				WT	2.889	0.564				
		D10	Long	KO	3.111	0.455		-0.971	0.387	
				WT	2.556	0.176				
		D11	Long	KO	2.556	0.580		-1.187	0.297	
				WT	3.000	0.167				
		D12	Long	KO	3.222	0.619		-0.548	0.605	
				WT	2.667	0.373				
		D13	Long	KO	2.889	0.423		-0.092	0.931	
				WT	2.889	0.261				
		D14	Long	KO	2.778	0.324		-0.144	0.931	
				WT	2.778	0.222				
		D15	Long	KO	3.333	0.441		-0.867	0.436	
				WT	2.889	0.309				
		D16	Long	KO	3.222	0.364		-0.188	0.863	
				WT	3.111	0.200				
Table S3	Figure S2D	D0	Short	KO	1.444	0.176	Mann-Whitney U Test	-2.585	0.024	*
				WT	0.667	0.167				
		D1	Short	KO	1.333	0.236		-1.222	0.297	
				WT	1.000	0.289				
		D2	Short	KO	1.556	0.242		-1.675	0.136	
				WT	1.000	0.236				
		D3	Short	KO	1.667	0.289		-0.372	0.730	
				WT	1.556	0.338				
		D4	Short	KO	2.444	0.444		-0.523	0.666	
				WT	2.000	0.236				
		D5	Short	KO	2.111	0.261		-0.595	0.605	
				WT	2.333	0.167				
		D6	Short	KO	2.667	0.289		-1.866	0.077	
				WT	1.889	0.261				
		D7	Short	KO	2.889	0.423		-1.509	0.161	
				WT	2.000	0.289				
		D8	Short	KO	3.111	0.484		-1.655	0.113	
				WT	2.111	0.261				
		D9	Short	KO	2.556	0.294		-0.241	0.863	
				WT	2.444	0.242				
		D10	Short	KO	2.778	0.222		-0.530	0.666	
				WT	2.556	0.294				
		D11	Short	KO	2.778	0.465		-0.046	1.000	
				WT	2.778	0.278				
		D12	Short	KO	3.000	0.601		-0.091	0.931	
				WT	2.778	0.222				
		D13	Short	KO	3.333	0.471		-2.062	0.063	
				WT	2.222	0.147				
		D14	Short	KO	3.444	0.475		-1.521	0.161	
				WT	2.444	0.294				
		D15	Short	KO	3.000	0.373		-1.011	0.340	
				WT	2.667	0.471				
		D16	Short	KO	3.111	0.423		-1.235	0.258	
				WT	2.444	0.294				

FHE peak frequency

Table	Related Figure	Day	Group	Condition	Mean	SEM	Stastistical test	Z	P	*
Table S3	Figure S2D	D0	KO	Long	1.444	0.338	Wilcoxon Signed Ranks Test	-0.108	0.914	
				Short	1.444	0.176				
		D1	KO	Long	0.778	0.222		-1.667	0.096	
				Short	1.333	0.236				
		D2	KO	Long	1.222	0.278		-1.732	0.083	
				Short	1.556	0.242				
		D3	KO	Long	1.778	0.434		-0.276	0.783	
				Short	1.667	0.289				
		D4	KO	Long	1.889	0.389		-1.406	0.160	
				Short	2.444	0.444				
		D5	KO	Long	1.778	0.324		-1.342	0.180	
				Short	2.111	0.261				
		D6	KO	Long	2.667	0.333		0.000	1.000	
				Short	2.667	0.289				
		D7	KO	Long	2.667	0.333		-0.632	0.527	
				Short	2.889	0.423				
		D8	KO	Long	2.778	0.401		-0.707	0.480	
				Short	3.111	0.484				
		D9	KO	Long	3.111	0.389		-1.633	0.102	
				Long	2.556	0.294				
		D10	KO	Short	3.111	0.455		-1.000	0.317	
				Long	2.778	0.222				
		D11	KO	Short	2.556	0.580		-0.520	0.603	
				Long	2.778	0.465				
		D12	KO	Short	3.222	0.619		-0.439	0.660	
				Long	3.000	0.601				
		D13	KO	Short	2.889	0.423		-1.027	0.305	
				Long	3.333	0.471				
		D14	KO	Short	2.778	0.324		-1.730	0.084	
				Long	3.444	0.475				
		D15	KO	Short	3.333	0.441		-0.780	0.435	
				Long	3.000	0.373				
		D16	KO	Short	3.222	0.364		-0.431	0.666	
				Long	3.111	0.423				
Table S3	Figure S2D	D0	WT	Long	0.667	0.236	Wilcoxon Signed Ranks Test	0.000	1.000	
				Short	0.667	0.167				
		D1	WT	Long	0.778	0.222		-1.000	0.317	
				Short	1.000	0.289				
		D2	WT	Long	1.333	0.373		-1.134	0.257	
				Short	1.000	0.236				
		D3	WT	Long	0.889	0.261		-1.897	0.058	
				Short	1.556	0.338				
		D4	WT	Long	1.889	0.309		-0.378	0.705	
				Short	2.000	0.236				
		D5	WT	Long	2.111	0.351		-0.632	0.527	
				Short	2.333	0.167				
		D6	WT	Long	2.444	0.338		-1.406	0.160	
				Short	1.889	0.261				
		D7	WT	Long	2.333	0.333		-0.749	0.454	
				Short	2.000	0.289				
		D8	WT	Long	2.222	0.147		-0.378	0.705	
				Short	2.111	0.261				
		D9	WT	Long	2.889	0.564		-0.597	0.551	
				Long	2.444	0.242				
		D10	WT	Short	2.556	0.176		0.000	1.000	
				Long	2.556	0.294				
		D11	WT	Short	3.000	0.167		-1.000	0.317	
				Long	2.778	0.278				
		D12	WT	Short	2.667	0.373		-0.378	0.705	
				Long	2.778	0.222				
		D13	WT	Short	2.889	0.261		-1.730	0.084	
				Long	2.222	0.147				
		D14	WT	Short	2.778	0.222		-0.828	0.408	
				Long	2.444	0.294				
		D15	WT	Short	2.889	0.309		-0.702	0.483	
				Long	2.667	0.471				
		D16	WT	Short	3.111	0.200		-1.667	0.096	
				Long	2.444	0.294				

3_4

FHE peak latency

Table	Related Figure	Day	Condition	Group	Mean	SEM	Stastistical test	Z	P	*
Table S3	Figure S2D	D0	Long	KO	3.556	1.029	Mann-Whitney U Test	-1.993	0.063	
				WT	1.444	0.959				
		D1	Long	KO	1.000	0.408		-0.389	0.730	
				WT	1.667	0.764				
		D2	Long	KO	2.667	1.027		-0.181	0.863	
				WT	3.111	1.274				
		D3	Long	KO	2.556	0.835		-0.975	0.387	
				WT	1.556	0.747				
		D4	Long	KO	2.444	0.930		-0.869	0.436	
				WT	1.222	0.434				
		D5	Long	KO	2.778	1.051		-0.677	0.546	
				WT	3.000	0.782				
		D6	Long	KO	3.111	1.006		-0.045	1.000	
				WT	3.222	0.969				
		D7	Long	KO	2.000	0.667		-1.083	0.297	
				WT	3.667	0.972				
		D8	Long	KO	1.000	0.373		-1.814	0.077	
				WT	2.444	0.626				
		D9	Long	KO	1.889	0.423		-0.547	0.605	
				WT	1.556	0.412				
		D10	Long	KO	2.444	0.412		-1.959	0.063	
				WT	1.222	0.364				
		D11	Long	KO	2.444	0.709		-0.723	0.489	
				WT	3.556	0.915				
		D12	Long	KO	1.333	0.167		-0.153	0.931	
				WT	1.444	0.377				
		D13	Long	KO	2.000	0.782		-0.790	0.489	
				WT	2.333	0.577				
		D14	Long	KO	2.556	0.801		-0.136	0.931	
				WT	2.333	0.799				
		D15	Long	KO	2.000	0.527		-0.895	0.387	
				WT	3.333	0.957				
		D16	Long	KO	2.444	0.689		-1.490	0.161	
				WT	3.778	0.760				
Table S3	Figure S2D	D0	Short	KO	4.778	0.862	Mann-Whitney U Test	-0.808	0.436	
				WT	3.556	1.226				
		D1	Short	KO	2.333	0.913		-1.473	0.161	
				WT	3.889	0.807				
		D2	Short	KO	2.667	0.913		-0.495	0.666	
				WT	1.889	0.676				
		D3	Short	KO	3.222	1.038		-0.269	0.796	
				WT	2.556	0.766				
		D4	Short	KO	3.444	1.056		-2.139	0.040	*
				WT	0.667	0.236				
		D5	Short	KO	2.333	0.707		-1.072	0.297	
				WT	3.667	0.882				
		D6	Short	KO	2.889	0.696		-0.090	0.931	
				WT	2.889	0.904				
		D7	Short	KO	1.889	0.512		-0.725	0.489	
				WT	1.333	0.441				
		D8	Short	KO	1.889	0.389		-0.507	0.666	
				WT	2.222	0.894				
		D9	Short	KO	1.111	0.309		-2.653	0.008	**
				WT	3.000	0.471				
		D10	Short	KO	2.000	0.553		-0.095	0.931	
				WT	2.000	0.601				
		D11	Short	KO	1.556	0.503		-0.633	0.546	
				WT	2.333	0.764				
		D12	Short	KO	1.111	0.261		-1.338	0.222	
				WT	2.111	0.588				
		D13	Short	KO	2.111	0.423		-0.596	0.605	
				WT	2.333	0.441				
		D14	Short	KO	1.222	0.364		-0.654	0.546	
				WT	1.778	0.547				
		D15	Short	KO	1.444	0.556		-0.683	0.546	
				WT	2.222	0.795				
		D16	Short	KO	1.556	0.294		-0.320	0.796	
				WT	1.778	0.465				

FHE peak latency

Table	Related Figure	Day	Group	Condition	Mean	SEM	Stastistical test	Z	P	*
Table S3	Figure S2D	D0	KO	Long	3.556	1.029	Wilcoxon Signed Ranks Test	-0.704	0.481	
				Short	4.778	0.862				
		D1	KO	Long	1.000	0.408		-1.420	0.156	
				Short	2.333	0.913				
		D2	KO	Long	2.667	1.027		0.000	1.000	
				Short	2.667	0.913				
		D3	KO	Long	2.556	0.835		-0.282	0.778	
				Short	3.222	1.038				
		D4	KO	Long	2.444	0.930		-0.422	0.673	
				Short	3.444	1.056				
		D5	KO	Long	2.778	1.051		-0.750	0.453	
				Short	2.333	0.707				
		D6	KO	Long	3.111	1.006		-0.170	0.865	
				Short	2.889	0.696				
		D7	KO	Long	2.000	0.667		-0.355	0.723	
				Short	1.889	0.512				
		D8	KO	Long	1.000	0.373		-1.730	0.084	
				Short	1.889	0.389				
		D9	KO	Long	1.889	0.423		-1.633	0.102	
				Long	1.111	0.309				
		D10	KO	Short	2.444	0.412		-0.660	0.509	
				Long	2.000	0.553				
		D11	KO	Short	2.444	0.709		-0.940	0.347	
				Long	1.556	0.503				
		D12	KO	Short	1.333	0.167		-0.707	0.480	
				Long	1.111	0.261				
		D13	KO	Short	2.000	0.782		-0.271	0.786	
				Long	2.111	0.423				
		D14	KO	Short	2.556	0.801		-1.450	0.147	
				Long	1.222	0.364				
		D15	KO	Short	2.000	0.527		-0.736	0.461	
				Long	1.444	0.556				
		D16	KO	Short	2.444	0.689		-0.780	0.436	
				Long	1.556	0.294				
Table S3	Figure S2D	D0	WT	Long	1.444	0.959	Wilcoxon Signed Ranks Test	-1.153	0.249	
				Short	3.556	1.226				
		D1	WT	Long	1.667	0.764		-1.781	0.075	
				Short	3.889	0.807				
		D2	WT	Long	3.111	1.274		-0.933	0.351	
				Short	1.889	0.676				
		D3	WT	Long	1.556	0.747		-0.705	0.481	
				Short	2.556	0.766				
		D4	WT	Long	1.222	0.434		-1.222	0.222	
				Short	0.667	0.236				
		D5	WT	Long	3.000	0.782		-0.563	0.574	
				Short	3.667	0.882				
		D6	WT	Long	3.222	0.969		-0.426	0.670	
				Short	2.889	0.904				
		D7	WT	Long	3.667	0.972		-1.689	0.091	
				Short	1.333	0.441				
		D8	WT	Long	2.444	0.626		-0.181	0.856	
				Short	2.222	0.894				
		D9	WT	Long	1.556	0.412		-1.975	0.048	
				Long	3.000	0.471				
		D10	WT	Short	1.222	0.364		-0.962	0.336	
				Long	2.000	0.601				
		D11	WT	Short	3.556	0.915		-1.273	0.203	
				Long	2.333	0.764				
		D12	WT	Short	1.444	0.377		-0.948	0.343	
				Long	2.111	0.588				
		D13	WT	Short	2.333	0.577		-0.171	0.864	
				Long	2.333	0.441				
		D14	WT	Short	2.333	0.799		-0.877	0.380	
				Long	1.778	0.547				
		D15	WT	Short	3.333	0.957		-0.431	0.667	
				Long	2.222	0.795				
		D16	WT	Short	3.778	0.760		-2.401	0.016	*
				Long	1.778	0.465				

3_5

HE vs. FHE peak frequencies (KO)

Table	Related Figure	Day	Condition	Pair	Mean	SEM	Stastistical test	Z	P	*
Table S3	Figure S3A	D0	Long	HE	1.222	0.222	Wilcoxon Signed Ranks Test	-1.414	0.315	
				FHE	1.440	1.030				
		D1	Long	HE	0.778	0.222		0.000	1.000	
				FHE	0.778	0.222				
		D2	Long	HE	1.222	0.278		0.000	1.000	
				FHE	1.222	0.278				
		D3	Long	HE	2.222	0.494		-1.633	0.205	
				FHE	1.778	0.434				
		D4	Long	HE	2.778	0.619		-1.841	0.131	
				FHE	1.889	0.389				
		D5	Long	HE	2.889	0.423		-2.456	0.028	*
				FHE	1.778	0.324				
		D6	Long	HE	4.111	0.423		-2.232	0.051	
				FHE	2.667	0.333				
		D7	Long	HE	3.889	0.676		-1.826	0.136	
				FHE	2.667	0.333				
		D8	Long	HE	5.222	0.683		-2.558	0.021	*
				FHE	2.778	0.401				
		D9	Long	HE	5.222	0.596		-2.555	0.021	*
				FHE	3.111	0.389				
		D10	Long	HE	5.111	0.611		-2.388	0.034	*
				FHE	3.111	0.455				
		D11	Long	HE	5.111	0.964		-2.692	0.014	**
				FHE	2.556	0.580				
		D12	Long	HE	4.444	0.648		-2.060	0.079	
				FHE	3.222	0.619				
		D13	Long	HE	5.333	0.667		-2.539	0.022	*
				FHE	2.889	0.423				
		D14	Long	HE	4.222	0.401		-2.565	0.021	*
				FHE	2.778	0.324				
		D15	Long	HE	4.667	0.471		-2.401	0.033	*
				FHE	3.333	0.441				
		D16	Long	HE	4.667	0.471		-2.060	0.079	
				FHE	3.222	0.364				
Table S3	Figure S3A	D0	Srhot	HE	1.111	0.200	Wilcoxon Signed Ranks Test	-1.732	0.167	
				FHE	1.444	0.862				
		D1	Srhot	HE	1.889	0.588		-0.816	0.828	
				FHE	1.333	0.236				
		D2	Srhot	HE	2.111	0.309		-1.890	0.118	
				FHE	1.556	0.242				
		D3	Srhot	HE	2.556	0.444		-1.841	0.131	
				FHE	1.667	0.289				
		D4	Srhot	HE	2.889	0.588		-2.000	0.091	
				FHE	2.444	0.444				
		D5	Srhot	HE	2.667	0.236		-1.633	0.205	
				FHE	2.111	0.261				
		D6	Srhot	HE	3.444	0.530		-1.841	0.131	
				FHE	2.667	0.289				
		D7	Srhot	HE	3.556	0.444		-2.121	0.068	
				FHE	2.889	0.423				
		D8	Srhot	HE	4.667	0.624		-2.410	0.032	*
				FHE	3.111	0.484				
		D9	Srhot	HE	3.667	0.624		-1.841	0.131	
				FHE	2.556	0.294				
		D10	Srhot	HE	3.222	0.278		-2.000	0.091	
				FHE	2.778	0.222				
		D11	Srhot	HE	3.778	0.364		-2.264	0.047	*
				FHE	2.778	0.465				
		D12	Srhot	HE	3.778	0.547		-1.890	0.118	
				FHE	3.000	0.601				
		D13	Srhot	HE	3.556	0.475		-1.414	0.315	
				FHE	3.333	0.471				
		D14	Srhot	HE	4.222	0.547		-1.841	0.131	
				FHE	3.444	0.475				
		D15	Srhot	HE	3.444	0.648		-1.342	0.359	
				FHE	3.000	0.373				
		D16	Srhot	HE	3.444	0.556		-1.732	0.167	
				FHE	3.111	0.423				

HE vs. FHE peak frequencies (WT)

Table	Related Figure	Day	Condition	Pair	Mean	SEM	Stastistical test	Z	P	*
Table S3	Figure S3A	D0	Long	HE	0.778	0.434	Wilcoxon Signed Ranks Test	-0.447	1.000	
				FHE	0.670	0.960				
		D1	Long	HE	1.000	0.289		-0.707	0.959	
				FHE	0.778	0.222				
		D2	Long	HE	1.667	0.471		-0.966	0.668	
				FHE	1.333	0.373				
		D3	Long	HE	2.111	0.351		-2.428	0.030	*
				FHE	0.889	0.261				
		D4	Long	HE	2.444	0.294		-1.890	0.118	
				FHE	1.889	0.309				
		D5	Long	HE	3.000	0.527		-1.841	0.131	
				FHE	2.111	0.351				
		D6	Long	HE	3.222	0.278		-2.070	0.077	
				FHE	2.444	0.338				
		D7	Long	HE	3.333	0.408		-2.264	0.047	*
				FHE	2.333	0.333				
		D8	Long	HE	3.556	0.338		-2.414	0.032	*
				FHE	2.222	0.147				
		D9	Long	HE	4.222	0.662		-2.264	0.047	*
				FHE	2.889	0.564				
		D10	Long	HE	3.889	0.261		-2.585	0.019	*
				FHE	2.556	0.176				
		D11	Long	HE	4.444	0.556		-2.226	0.052	
				FHE	3.000	0.167				
		D12	Long	HE	4.667	0.577		-2.410	0.032	*
				FHE	2.667	0.373				
		D13	Long	HE	4.333	0.373		-2.414	0.032	*
				FHE	2.889	0.261				
		D14	Long	HE	4.333	0.408		-2.401	0.033	*
				FHE	2.778	0.222				
		D15	Long	HE	4.444	0.377		-2.585	0.019	*
				FHE	2.889	0.309				
		D16	Long	HE	5.000	0.408		-2.555	0.021	*
				FHE	3.111	0.200				
Table S3	Figure S3A	D0	Srhot	HE	1.111	0.351	Wilcoxon Signed Ranks Test	-1.414	0.315	
				FHE	0.667	1.226				
		D1	Srhot	HE	1.000	0.236		0.000	1.000	
				FHE	1.000	0.289				
		D2	Srhot	HE	1.222	0.222		-1.000	0.635	
				FHE	1.000	0.236				
		D3	Srhot	HE	2.667	0.500		-2.456	0.028	*
				FHE	1.556	0.338				
		D4	Srhot	HE	2.889	0.564		-1.604	0.218	
				FHE	2.000	0.236				
		D5	Srhot	HE	3.222	0.547		-1.633	0.205	
				FHE	2.333	0.167				
		D6	Srhot	HE	2.333	0.373		-2.000	0.091	
				FHE	1.889	0.261				
		D7	Srhot	HE	3.111	0.351		-2.640	0.017	**
				FHE	2.000	0.289				
		D8	Srhot	HE	3.000	0.289		-2.271	0.046	*
				FHE	2.111	0.261				
		D9	Srhot	HE	3.444	0.503		-2.121	0.068	
				FHE	2.444	0.242				
		D10	Srhot	HE	3.333	0.289		-1.890	0.118	
				FHE	2.556	0.294				
		D11	Srhot	HE	3.333	0.471		-1.633	0.205	
				FHE	2.778	0.278				
		D12	Srhot	HE	3.778	0.364		-1.841	0.131	
				FHE	2.778	0.222				
		D13	Srhot	HE	2.444	0.176		-1.414	0.315	
				FHE	2.222	0.147				
		D14	Srhot	HE	3.111	0.261		-2.121	0.068	
				FHE	2.444	0.294				
		D15	Srhot	HE	3.444	0.444		-1.890	0.118	
				FHE	2.667	0.471				
		D16	Srhot	HE	2.889	0.200		-1.633	0.205	
				FHE	2.444	0.294				

3_6

HE vs. FHE peak latencies (KO)

Table	Related Figure	Day	Condition	Pair	Mean	SEM	Stastistical test	Z	P	*
Table S3	Figure S3A	D0	Long	HE	4.000	1.093	Wilcoxon Signed Ranks Test	-1.342	0.359	
				FHE	3.556	1.029				
		D1	Long	HE	1.000	0.408		0.000	1.000	
				FHE	1.000	0.408				
		D2	Long	HE	4.222	1.310		-1.604	0.218	
				FHE	2.667	1.027				
		D3	Long	HE	3.667	1.291		-0.447	1.000	
				FHE	2.556	0.835				
		D4	Long	HE	2.889	1.296		-0.447	1.000	
				FHE	2.444	0.930				
		D5	Long	HE	4.667	1.202		-0.813	0.833	
				FHE	2.778	1.051				
		D6	Long	HE	4.222	1.164		-0.730	0.930	
				FHE	3.111	1.006				
		D7	Long	HE	3.556	1.215		-1.342	0.359	
				FHE	2.000	0.667				
		D8	Long	HE	3.222	1.382		-1.826	0.136	
				FHE	1.000	0.373				
		D9	Long	HE	5.000	1.333		-1.841	0.131	
				FHE	1.889	0.423				
		D10	Long	HE	5.111	1.172		-1.753	0.159	
				FHE	2.444	0.412				
		D11	Long	HE	5.556	1.600		-1.378	0.336	
				FHE	2.444	0.709				
		D12	Long	HE	3.111	1.252		-1.095	0.547	
				FHE	1.333	0.167				
		D13	Long	HE	5.111	1.348		-2.252	0.049	*
				FHE	2.000	0.782				
		D14	Long	HE	3.889	0.889		-1.841	0.131	
				FHE	2.556	0.801				
		D15	Long	HE	5.222	1.579		-1.826	0.136	
				FHE	2.000	0.527				
		D16	Long	HE	5.000	1.414		-1.992	0.093	
				FHE	2.444	0.689				
Table S3	Figure S3A	D0	Srhot	HE	3.667	1.312	Wilcoxon Signed Ranks Test	-0.813	0.833	
				FHE	4.778	0.862				
		D1	Srhot	HE	3.333	1.312		-0.730	0.930	
				FHE	2.333	0.913				
		D2	Srhot	HE	6.222	1.289		-1.992	0.093	
				FHE	2.667	0.913				
		D3	Srhot	HE	5.667	1.607		-1.342	0.359	
				FHE	3.222	1.038				
		D4	Srhot	HE	3.444	1.271		0.000	1.000	
				FHE	3.444	1.056				
		D5	Srhot	HE	2.667	0.645		-1.000	0.635	
				FHE	2.333	0.707				
		D6	Srhot	HE	2.778	0.741		-1.000	0.635	
				FHE	2.889	0.696				
		D7	Srhot	HE	2.778	1.392		-0.136	1.000	
				FHE	1.889	0.512				
		D8	Srhot	HE	3.333	1.067		-1.841	0.131	
				FHE	1.889	0.389				
		D9	Srhot	HE	2.556	0.801		-1.633	0.205	
				FHE	1.111	0.309				
		D10	Srhot	HE	2.222	0.683		-0.447	1.000	
				FHE	2.000	0.553				
		D11	Srhot	HE	4.000	1.190		-1.625	0.208	
				FHE	1.556	0.503				
		D12	Srhot	HE	2.111	0.772		-1.069	0.570	
				FHE	1.111	0.261				
		D13	Srhot	HE	2.556	0.899		-0.447	1.000	
				FHE	2.111	0.423				
		D14	Srhot	HE	1.667	0.408		-1.069	0.570	
				FHE	1.222	0.364				
		D15	Srhot	HE	1.333	0.577		-1.000	0.635	
				FHE	1.444	0.556				
		D16	Srhot	HE	1.444	0.377		-0.368	1.000	
				FHE	1.556	0.294				

HE vs. FHE peak latencies (WT)

Table	Related Figure	Day	Condition	Pair	Mean	SEM	Stastistical test	Z	P	*
Table S3	Figure S3A	D0	Long	HE	1.778	1.222	Wilcoxon Signed Ranks Test	-1.000	0.635	
				FHE	1.444	0.959				
		D1	Long	HE	2.000	1.443		0.000	1.000	
				FHE	1.667	0.764				
		D2	Long	HE	3.111	1.438		0.000	1.000	
				FHE	3.111	1.274				
		D3	Long	HE	5.111	1.728		-1.753	0.159	
				FHE	1.556	0.747				
		D4	Long	HE	2.111	1.111		-0.730	0.930	
				FHE	1.222	0.434				
		D5	Long	HE	5.444	1.608		-1.342	0.359	
				FHE	3.000	0.782				
		D6	Long	HE	5.111	1.476		-1.604	0.218	
				FHE	3.222	0.969				
		D7	Long	HE	4.222	0.572		-1.089	0.553	
				FHE	3.667	0.972				
		D8	Long	HE	3.222	1.278		-0.593	1.000	
				FHE	2.444	0.626				
		D9	Long	HE	4.000	1.179		-2.023	0.086	
				FHE	1.556	0.412				
		D10	Long	HE	3.111	1.060		-1.581	0.228	
				FHE	1.222	0.364				
		D11	Long	HE	5.556	1.365		-0.931	0.703	
				FHE	3.556	0.915				
		D12	Long	HE	3.778	1.245		-1.841	0.131	
				FHE	1.444	0.377				
		D13	Long	HE	2.889	0.633		-1.289	0.395	
				FHE	2.333	0.577				
		D14	Long	HE	4.333	0.833		-2.032	0.084	
				FHE	2.333	0.799				
		D15	Long	HE	4.333	0.667		-1.633	0.205	
				FHE	3.333	0.957				
		D16	Long	HE	5.778	1.103		-1.980	0.096	
				FHE	3.778	0.760				
Table S3	Figure S3A	D0	Srhot	HE	4.000	1.616	Wilcoxon Signed Ranks Test	-0.405	1.000	
				FHE	3.556	1.226				
		D1	Srhot	HE	4.333	1.258		-0.535	1.000	
				FHE	3.889	0.807				
		D2	Srhot	HE	1.889	1.020		-0.368	1.000	
				FHE	1.889	0.676				
		D3	Srhot	HE	3.556	1.692		-0.736	0.923	
				FHE	2.556	0.766				
		D4	Srhot	HE	2.333	1.202		-1.069	0.570	
				FHE	0.667	0.236				
		D5	Srhot	HE	2.667	0.816		-1.342	0.359	
				FHE	3.667	0.882				
		D6	Srhot	HE	1.778	0.969		-1.604	0.218	
				FHE	2.889	0.904				
		D7	Srhot	HE	4.111	1.567		-1.753	0.159	
				FHE	1.333	0.441				
		D8	Srhot	HE	4.444	1.271		-1.476	0.280	
				FHE	2.222	0.894				
		D9	Srhot	HE	3.889	0.484		-1.342	0.359	
				FHE	3.000	0.471				
		D10	Srhot	HE	4.000	1.404		-1.214	0.450	
				FHE	2.000	0.601				
		D11	Srhot	HE	3.889	1.047		-1.604	0.218	
				FHE	2.333	0.764				
		D12	Srhot	HE	3.222	0.862		-1.461	0.288	
				FHE	2.111	0.588				
		D13	Srhot	HE	2.222	0.521		-0.447	1.000	
				FHE	2.333	0.441				
		D14	Srhot	HE	3.778	1.064		-1.633	0.205	
				FHE	1.778	0.547				
		D15	Srhot	HE	3.333	0.726		-1.841	0.131	
				FHE	2.222	0.795				
		D16	Srhot	HE	2.000	0.471		-0.447	1.000	
				FHE	1.778	0.465				

3_7

Correlation between HE and FHE peak frequencies (KO)

Table	Related Figure	Day	Condition	Pair	Mean	SEM	Stastistical test	Spearman's ρ	P	*
Table S3	Figure S3B	D0	Long	HE	1.222	0.222	Spearman's rank correlation coefficient	0.266	0.976	
				FHE	1.440	1.030				
		D1	Long	HE	0.778	0.222		0.813	0.016	*
				FHE	0.778	0.222				
		D2	Long	HE	1.222	0.278		0.767	0.032	*
				FHE	1.222	0.278				
		D3	Long	HE	2.222	0.494		0.815	0.015	*
				FHE	1.778	0.434				
		D4	Long	HE	2.778	0.619		0.700	0.071	
				FHE	1.889	0.389				
		D5	Long	HE	2.889	0.423		0.523	0.298	
				FHE	1.778	0.324				
		D6	Long	HE	4.111	0.423		0.454	0.440	
				FHE	2.667	0.333				
		D7	Long	HE	3.889	0.676		0.467	0.409	
				FHE	2.667	0.333				
		D8	Long	HE	5.222	0.683		0.860	0.006	**
				FHE	2.778	0.401				
		D9	Long	HE	5.222	0.596		0.624	0.145	
				FHE	3.111	0.389				
		D10	Long	HE	5.111	0.611		0.557	0.238	
				FHE	3.111	0.455				
		D11	Long	HE	5.111	0.964		0.749	0.040	*
				FHE	2.556	0.580				
		D12	Long	HE	4.444	0.648		0.306	0.847	
				FHE	3.222	0.619				
		D13	Long	HE	5.333	0.667		0.681	0.087	
				FHE	2.889	0.423				
		D14	Long	HE	4.222	0.401		0.600	0.175	
				FHE	2.778	0.324				
		D15	Long	HE	4.667	0.471		0.754	0.038	*
				FHE	3.333	0.441				
		D16	Long	HE	4.667	0.471		0.258	1.000	
				FHE	3.222	0.364				
Table S3	Figure S3B	D0	Srhot	HE	1.111	0.200	Spearman's rank correlation coefficient	-0.132	1.000	
				FHE	1.444	0.862				
		D1	Srhot	HE	1.889	0.588		0.536	0.273	
				FHE	1.333	0.236				
		D2	Srhot	HE	2.111	0.309		0.729	0.052	
				FHE	1.556	0.242				
		D3	Srhot	HE	2.556	0.444		0.102	1.000	
				FHE	1.667	0.289				
		D4	Srhot	HE	2.889	0.588		0.974	0.000	***
				FHE	2.444	0.444				
		D5	Srhot	HE	2.667	0.236		0.220	1.000	
				FHE	2.111	0.261				
		D6	Srhot	HE	3.444	0.530		0.759	0.035	*
				FHE	2.667	0.289				
		D7	Srhot	HE	3.556	0.444		0.862	0.006	**
				FHE	2.889	0.423				
		D8	Srhot	HE	4.667	0.624		0.522	0.299	
				FHE	3.111	0.484				
		D9	Srhot	HE	3.667	0.624		0.466	0.411	
				FHE	2.556	0.294				
		D10	Srhot	HE	3.222	0.278		0.787	0.024	*
				FHE	2.778	0.222				
		D11	Srhot	HE	3.778	0.364		0.696	0.074	
				FHE	2.778	0.465				
		D12	Srhot	HE	3.778	0.547		0.798	0.020	*
				FHE	3.000	0.601				
		D13	Srhot	HE	3.556	0.475		0.964	0.000	***
				FHE	3.333	0.471				
		D14	Srhot	HE	4.222	0.547		0.781	0.026	*
				FHE	3.444	0.475				
		D15	Srhot	HE	3.444	0.648		0.929	0.001	**
				FHE	3.000	0.373				
		D16	Srhot	HE	3.444	0.556		0.987	0.000	***
				FHE	3.111	0.423				

Correlation between HE and FHE peak frequencies (WT)

Table	Related Figure	Day	Condition	Pair	Mean	SEM	Stastistical test	Spearman's ρ	P	*
Table S3	Figure S3B	D0	Long	HE	0.778	0.434	Spearman's rank correlation coefficient	0.714	0.061	
				FHE	0.670	0.960				
		D1	Long	HE	1.000	0.289		0.256	1.000	
				FHE	0.778	0.222				
		D2	Long	HE	1.667	0.471		0.694	0.076	
				FHE	1.333	0.373				
		D3	Long	HE	2.111	0.351		0.682	0.086	
				FHE	0.889	0.261				
		D4	Long	HE	2.444	0.294		0.768	0.031	*
				FHE	1.889	0.309				
		D5	Long	HE	3.000	0.527		0.498	0.345	
				FHE	2.111	0.351				
		D6	Long	HE	3.222	0.278		0.588	0.191	
				FHE	2.444	0.338				
		D7	Long	HE	3.333	0.408		0.643	0.124	
				FHE	2.333	0.333				
		D8	Long	HE	3.556	0.338		-0.371	0.651	
				FHE	2.222	0.147				
		D9	Long	HE	4.222	0.662		0.666	0.101	
				FHE	2.889	0.564				
		D10	Long	HE	3.889	0.261		0.463	0.419	
				FHE	2.556	0.176				
		D11	Long	HE	4.444	0.556		0.283	0.920	
				FHE	3.000	0.167				
		D12	Long	HE	4.667	0.577		0.461	0.424	
				FHE	2.667	0.373				
		D13	Long	HE	4.333	0.373		0.469	0.405	
				FHE	2.889	0.261				
		D14	Long	HE	4.333	0.408		0.240	1.000	
				FHE	2.778	0.222				
		D15	Long	HE	4.444	0.377		0.340	0.742	
				FHE	2.889	0.309				
		D16	Long	HE	5.000	0.408		-0.179	1.000	
				FHE	3.111	0.200				
Table S3	Figure S3B	D0	Short	HE	1.111	0.351	Spearman's rank correlation coefficient	0.297	0.877	
				FHE	0.667	1.226				
		D1	Short	HE	1.000	0.236		0.887	0.003	**
				FHE	1.000	0.289				
		D2	Short	HE	1.222	0.222		0.505	0.331	
				FHE	1.000	0.236				
		D3	Short	HE	2.667	0.500		0.838	0.010	*
				FHE	1.556	0.338				
		D4	Short	HE	2.889	0.564		0.378	0.632	
				FHE	2.000	0.236				
		D5	Short	HE	3.222	0.547		0.513	0.316	
				FHE	2.333	0.167				
		D6	Short	HE	2.333	0.373		0.910	0.001	**
				FHE	1.889	0.261				
		D7	Short	HE	3.111	0.351		0.849	0.008	**
				FHE	2.000	0.289				
		D8	Short	HE	3.000	0.289		0.535	0.275	
				FHE	2.111	0.261				
		D9	Short	HE	3.444	0.503		0.412	0.540	
				FHE	2.444	0.242				
		D10	Short	HE	3.333	0.289		0.400	0.572	
				FHE	2.556	0.294				
		D11	Short	HE	3.333	0.471		0.811	0.016	*
				FHE	2.778	0.278				
		D12	Short	HE	3.778	0.364		-0.318	0.809	
				FHE	2.778	0.222				
		D13	Short	HE	2.444	0.176		0.598	0.178	
				FHE	2.222	0.147				
		D14	Short	HE	3.111	0.261		0.611	0.160	
				FHE	2.444	0.294				
		D15	Short	HE	3.444	0.444		0.734	0.049	*
				FHE	2.667	0.471				
		D16	Short	HE	2.889	0.200		0.551	0.248	
				FHE	2.444	0.294				

3_8

Correlation between HE and FHE peak latencies (KO)

Table	Related Figure	Day	Condition	Paiir	Mean	SEM	Stastistical test	Spearman's ρ	P	*
Table S3	Figure S3B	D0	Long	HE	4.000	1.093	Spearman's rank correlation coefficient	0.953	0.000	***
				FHE	3.556	1.029				
		D1	Long	HE	1.000	0.408		1.000	0.000	***
				FHE	1.000	0.408				
		D2	Long	HE	4.222	1.310		0.843	0.009	**
				FHE	2.667	1.027				
		D3	Long	HE	3.667	1.291		0.734	0.049	*
				FHE	2.556	0.835				
		D4	Long	HE	2.889	1.296		0.148	1.000	
				FHE	2.444	0.930				
		D5	Long	HE	4.667	1.202		-0.090	1.000	
				FHE	2.778	1.051				
		D6	Long	HE	4.222	1.164		0.147	1.000	
				FHE	3.111	1.006				
		D7	Long	HE	3.556	1.215		0.056	1.000	
				FHE	2.000	0.667				
		D8	Long	HE	3.222	1.382		0.798	0.020	*
				FHE	1.000	0.373				
		D9	Long	HE	5.000	1.333		-0.087	1.000	
				FHE	1.889	0.423				
		D10	Long	HE	5.111	1.172		-0.364	0.671	
				FHE	2.444	0.412				
		D11	Long	HE	5.556	1.600		0.194	1.000	
				FHE	2.444	0.709				
		D12	Long	HE	3.111	1.252		-0.189	1.000	
				FHE	1.333	0.167				
		D13	Long	HE	5.111	1.348		0.252	1.000	
				FHE	2.000	0.782				
		D14	Long	HE	3.889	0.889		0.537	0.273	
				FHE	2.556	0.801				
		D15	Long	HE	5.222	1.579		0.186	1.000	
				FHE	2.000	0.527				
		D16	Long	HE	5.000	1.414		0.698	0.073	
				FHE	2.444	0.689				
Table S3	Figure S3B	D0	Srhot	HE	3.667	1.312	Spearman's rank correlation coefficient	0.200	1.000	
				FHE	4.778	0.862				
		D1	Srhot	HE	3.333	1.312		0.485	0.372	
				FHE	2.333	0.913				
		D2	Srhot	HE	6.222	1.289		-0.127	1.000	
				FHE	2.667	0.913				
		D3	Srhot	HE	5.667	1.607		0.357	0.690	
				FHE	3.222	1.038				
		D4	Srhot	HE	3.444	1.271		0.569	0.220	
				FHE	3.444	1.056				
		D5	Srhot	HE	2.667	0.645		0.870	0.005	**
				FHE	2.333	0.707				
		D6	Srhot	HE	2.778	0.741		0.996	0.000	***
				FHE	2.889	0.696				
		D7	Srhot	HE	2.778	1.392		0.089	1.000	
				FHE	1.889	0.512				
		D8	Srhot	HE	3.333	1.067		0.764	0.033	*
				FHE	1.889	0.389				
		D9	Srhot	HE	2.556	0.801		0.244	1.000	
				FHE	1.111	0.309				
		D10	Srhot	HE	2.222	0.683		0.450	0.448	
				FHE	2.000	0.553				
		D11	Srhot	HE	4.000	1.190		-0.181	1.000	
				FHE	1.556	0.503				
		D12	Srhot	HE	2.111	0.772		0.418	0.527	
				FHE	1.111	0.261				
		D13	Srhot	HE	2.556	0.899		0.969	0.000	***
				FHE	2.111	0.423				
		D14	Srhot	HE	1.667	0.408		0.474	0.395	
				FHE	1.222	0.364				
		D15	Srhot	HE	1.333	0.577		0.932	0.000	***
				FHE	1.444	0.556				
		D16	Srhot	HE	1.444	0.377		0.160	1.000	
				FHE	1.556	0.294				

Correlation between HE and FHE peak latencies (WT)

Table	Related Figure	Day	Condition	Pair	Mean	SEM	Stastistical test	Spearman's ρ	P	*
Table S3	Figure S3B	D0	Long	HE	1.778	1.222	Spearman's rank correlation coefficient	1.000	0.000	***
				FHE	1.444	0.959				
		D1	Long	HE	2.000	1.443		0.033	1.000	
				FHE	1.667	0.764				
		D2	Long	HE	3.111	1.438		0.948	0.000	***
				FHE	3.111	1.274				
		D3	Long	HE	5.111	1.728		0.037	1.000	
				FHE	1.556	0.747				
		D4	Long	HE	2.111	1.111		0.520	0.303	
				FHE	1.222	0.434				
		D5	Long	HE	5.444	1.608		0.457	0.433	
				FHE	3.000	0.782				
		D6	Long	HE	5.111	1.476		0.822	0.013	*
				FHE	3.222	0.969				
		D7	Long	HE	4.222	0.572		0.759	0.036	*
				FHE	3.667	0.972				
		D8	Long	HE	3.222	1.278		-0.270	0.964	
				FHE	2.444	0.626				
		D9	Long	HE	4.000	1.179		0.518	0.306	
				FHE	1.556	0.412				
		D10	Long	HE	3.111	1.060		-0.137	1.000	
				FHE	1.222	0.364				
		D11	Long	HE	5.556	1.365		-0.154	1.000	
				FHE	3.556	0.915				
		D12	Long	HE	3.778	1.245		0.476	0.391	
				FHE	1.444	0.377				
		D13	Long	HE	2.889	0.633		0.803	0.018	*
				FHE	2.333	0.577				
		D14	Long	HE	4.333	0.833		0.339	0.744	
				FHE	2.333	0.799				
		D15	Long	HE	4.333	0.667		0.673	0.094	
				FHE	3.333	0.957				
		D16	Long	HE	5.778	1.103		0.456	0.435	
				FHE	3.778	0.760				
Table S3	Figure S3B	D0	Short	HE	4.000	1.616	Spearman's rank correlation coefficient	0.045	1.000	
				FHE	3.556	1.226				
		D1	Short	HE	4.333	1.258		0.271	0.962	
				FHE	3.889	0.807				
		D2	Short	HE	1.889	1.020		0.354	0.699	
				FHE	1.889	0.676				
		D3	Short	HE	3.556	1.692		0.101	1.000	
				FHE	2.556	0.766				
		D4	Short	HE	2.333	1.202		0.019	1.000	
				FHE	0.667	0.236				
		D5	Short	HE	2.667	0.816		0.521	0.300	
				FHE	3.667	0.882				
		D6	Short	HE	1.778	0.969		0.692	0.078	
				FHE	2.889	0.904				
		D7	Short	HE	4.111	1.567		0.591	0.188	
				FHE	1.333	0.441				
		D8	Short	HE	4.444	1.271		-0.090	1.000	
				FHE	2.222	0.894				
		D9	Short	HE	3.889	0.484		0.372	0.649	
				FHE	3.000	0.471				
		D10	Short	HE	4.000	1.404		-0.064	1.000	
				FHE	2.000	0.601				
		D11	Short	HE	3.889	1.047		0.508	0.326	
				FHE	2.333	0.764				
		D12	Short	HE	3.222	0.862		0.487	0.367	
				FHE	2.111	0.588				
		D13	Short	HE	2.222	0.521		0.849	0.008	**
				FHE	2.333	0.441				
		D14	Short	HE	3.778	1.064		0.228	1.000	
				FHE	1.778	0.547				
		D15	Short	HE	3.333	0.726		0.461	0.423	
				FHE	2.222	0.795				
		D16	Short	HE	2.000	0.471		0.678	0.089	*
				FHE	1.778	0.465				

3_9

ㅍ										
Table	Related Figure	Day	Group	Condition	Mean	SEM	Stastistical test	Spearman's ρ	P	*
Table S3	Figure S3C	D0	KO	Long	4.000	1.093	Spearman's rank correlation coefficient	-0.434	0.244	
				Short	3.667	1.312				
		D1	KO	Long	1.000	0.408		-0.378	0.316	
				Short	3.333	1.312				
		D2	KO	Long	4.222	1.310		-0.352	0.353	
				Short	6.222	1.289				
		D3	KO	Long	3.667	1.291		-0.325	0.394	
				Short	5.667	1.607				
		D4	KO	Long	2.889	1.296		0.116	0.767	
				Short	3.444	1.271				
		D5	KO	Long	4.667	1.202		0.269	0.484	
				Short	2.667	0.645				
		D6	KO	Long	4.222	1.164		0.622	0.074	
				Short	2.778	0.741				
		D7	KO	Long	3.556	1.215		0.375	0.320	
				Short	2.778	1.392				
		D8	KO	Long	3.222	1.382		0.391	0.298	
				Short	3.333	1.067				
		D9	KO	Long	5.000	1.333		0.293	0.444	
				Long	2.556	0.801				
		D10	KO	Short	5.111	1.172		-0.004	0.991	
				Long	2.222	0.683				
		D11	KO	Short	5.556	1.600		-0.791	0.011	*
				Long	4.000	1.190				
		D12	KO	Short	3.111	1.252		-0.049	0.900	
				Long	2.111	0.772				
		D13	KO	Short	5.111	1.348		0.617	0.076	
				Long	2.556	0.899				
		D14	KO	Short	3.889	0.889		0.347	0.360	
				Long	1.667	0.408				
		D15	KO	Short	5.222	1.579		0.829	0.006	**
				Long	1.333	0.577				
		D16	KO	Short	5.000	1.414		0.471	0.201	
				Long	1.444	0.377				
Table S3	Figure S3C	D0	WT	Long	1.778	1.222	Spearman's rank correlation coefficient	-0.060	0.878	
				Short	4.000	1.616				
		D1	WT	Long	2.000	1.443		-0.060	0.878	
				Short	4.333	1.258				
		D2	WT	Long	3.111	1.438		-0.510	0.160	
				Short	1.889	1.020				
		D3	WT	Long	5.111	1.728		0.432	0.246	
				Short	3.556	1.692				
		D4	WT	Long	2.111	1.111		0.303	0.428	
				Short	2.333	1.202				
		D5	WT	Long	5.444	1.608		0.253	0.511	
				Short	2.667	0.816				
		D6	WT	Long	5.111	1.476		0.147	0.707	
				Short	1.778	0.969				
		D7	WT	Long	4.222	0.572		0.025	0.948	
				Short	4.111	1.567				
		D8	WT	Long	3.222	1.278		0.385	0.306	
				Short	4.444	1.271				
		D9	WT	Long	4.000	1.179		0.278	0.469	
				Long	3.889	0.484				
		D10	WT	Short	3.111	1.060		0.356	0.348	
				Long	4.000	1.404				
		D11	WT	Short	5.556	1.365		0.026	0.947	
				Long	3.889	1.047				
		D12	WT	Short	3.778	1.245		-0.469	0.203	
				Long	3.222	0.862				
		D13	WT	Short	2.889	0.633		0.444	0.232	
				Long	2.222	0.521				
		D14	WT	Short	4.333	0.833		0.562	0.115	
				Long	3.778	1.064				
		D15	WT	Short	4.333	0.667		0.376	0.319	
				Long	3.333	0.726				
		D16	WT	Short	5.778	1.103		0.602	0.086	
				Long	2.000	0.471				

4_1

HE frequency in 0–2 s window

Table	Related Figure	Day	Condition	Group	Mean	SEM	Stastistical test	Z	P	*
Table S4	Figure S4	D0	Long	KO	0.472	0.329	Mann-Whitney U Test	-0.369	0.796	
				WT	0.139	0.073				
		D1	Long	KO	0.278	0.153		-0.158	0.931	
				WT	0.222	0.121				
		D2	Long	KO	0.417	0.144		-0.093	0.931	
				WT	0.722	0.385				
		D3	Long	KO	0.889	0.470		-0.094	0.931	
				WT	0.778	0.426				
		D4	Long	KO	1.222	0.480		-0.585	0.605	
				WT	1.500	0.514				
		D5	Long	KO	1.333	0.520		-0.045	1.000	
				WT	1.556	0.586				
		D6	Long	KO	1.889	0.455		-0.982	0.340	
				WT	1.389	0.582				
		D7	Long	KO	2.389	0.881		-0.358	0.730	
				WT	1.500	0.433				
		D8	Long	KO	2.944	0.843		-0.133	0.931	
				WT	2.333	0.520				
		D9	Long	KO	3.111	0.666		-1.113	0.297	
				WT	2.056	0.530				
		D10	Long	KO	2.222	0.683		-0.224	0.863	
				WT	2.056	0.523				
		D11	Long	KO	2.611	0.865		-0.045	1.000	
				WT	2.111	0.594				
		D12	Long	KO	2.667	0.791		-0.312	0.796	
				WT	2.167	0.672				
		D13	Long	KO	3.222	1.087		-0.761	0.489	
				WT	1.833	0.577				
		D14	Long	KO	2.944	0.684		-0.844	0.436	
				WT	2.167	0.707				
		D15	Long	KO	2.833	0.874		-0.671	0.546	
				WT	2.111	0.790				
		D16	Long	KO	2.444	0.621		-0.935	0.387	
				WT	1.722	0.619				
Table S4	Figure S4	D0	Short	KO	0.222	0.114	Mann-Whitney U Test	-1.455	0.258	
				WT	0.056	0.056				
		D1	Short	KO	0.528	0.193		-2.842	0.014	*
				WT	0.000	0.000				
		D2	Short	KO	0.667	0.161		-1.230	0.258	
				WT	0.389	0.132				
		D3	Short	KO	0.944	0.269		-0.182	0.863	
				WT	0.889	0.247				
		D4	Short	KO	1.667	0.486		-0.359	0.730	
				WT	1.278	0.222				
		D5	Short	KO	1.611	0.545		-0.274	0.796	
				WT	1.111	0.274				
		D6	Short	KO	1.778	0.596		-0.787	0.489	
				WT	1.222	0.528				
		D7	Short	KO	2.111	0.588		-0.871	0.436	
				WT	1.333	0.354				
		D8	Short	KO	2.500	0.565		-1.250	0.222	
				WT	1.500	0.289				
		D9	Short	KO	2.444	0.444		-1.958	0.050	
				WT	1.222	0.364				
		D10	Short	KO	2.167	0.456		-0.802	0.436	
				WT	1.722	0.560				
		D11	Short	KO	2.333	0.514		-0.445	0.666	
				WT	2.111	0.633				
		D12	Short	KO	2.278	0.547		-0.934	0.387	
				WT	1.500	0.354				
		D13	Short	KO	2.167	0.777		-0.356	0.730	
				WT	1.333	0.363				
		D14	Short	KO	2.778	0.678		-1.332	0.190	
				WT	1.667	0.408				
		D15	Short	KO	2.556	0.679		-1.341	0.190	
				WT	1.111	0.351				
		D16	Short	KO	2.500	0.651		-0.980	0.340	
				WT	1.611	0.341				

HE frequency in 0–2 s window

Table	Related Figure	Day	Group	Condition	Mean	SEM	Stastistical test	Z	P	*
Table S4	Figure S4	D0	KO	Long	0.472	0.329	Wilcoxon Signed Ranks Test	-0.184	0.854	
				Short	0.222	0.114				
		D1	KO	Long	0.278	0.153		-1.510	0.131	
				Short	0.528	0.193				
		D2	KO	Long	0.417	0.144		-1.579	0.114	
				Short	0.667	0.161				
		D3	KO	Long	0.889	0.470		-0.170	0.865	
				Short	0.944	0.269				
		D4	KO	Long	1.222	0.480		-0.985	0.325	
				Short	1.667	0.486				
		D5	KO	Long	1.333	0.520		-0.632	0.527	
				Short	1.611	0.545				
		D6	KO	Long	1.889	0.455		-0.060	0.952	
				Short	1.778	0.596				
		D7	KO	Long	2.389	0.881		-0.680	0.497	
				Short	2.111	0.588				
		D8	KO	Long	2.944	0.843		-0.517	0.605	
				Short	2.500	0.565				
		D9	KO	Long	3.111	0.666		-1.131	0.258	
				Long	2.444	0.444				
		D10	KO	Short	2.222	0.683		-0.071	0.943	
				Long	2.167	0.456				
		D11	KO	Short	2.611	0.865		-0.141	0.888	
				Long	2.333	0.514				
		D12	KO	Short	2.667	0.791		-0.938	0.348	
				Long	2.278	0.547				
		D13	KO	Short	3.222	1.087		-2.120	0.034	*
				Long	2.167	0.777				
		D14	KO	Short	2.944	0.684		-0.531	0.595	
				Long	2.778	0.678				
		D15	KO	Short	2.833	0.874		-0.085	0.932	
				Long	2.556	0.679				
		D16	KO	Short	2.444	0.621		-0.106	0.915	
				Long	2.500	0.651				
Table S4	Figure S4	D0	WT	Long	0.139	0.073	Wilcoxon Signed Ranks Test	-1.342	0.180	
				Short	0.056	0.056				
		D1	WT	Long	0.222	0.121		-1.633	0.102	
				Short	0.000	0.000				
		D2	WT	Long	0.722	0.385		-0.341	0.733	
				Short	0.389	0.132				
		D3	WT	Long	0.778	0.426		-0.970	0.332	
				Short	0.889	0.247				
		D4	WT	Long	1.500	0.514		-0.512	0.609	
				Short	1.278	0.222				
		D5	WT	Long	1.556	0.586		-0.923	0.356	
				Short	1.111	0.274				
		D6	WT	Long	1.389	0.582		-0.351	0.725	
				Short	1.222	0.528				
		D7	WT	Long	1.500	0.433		-0.849	0.396	
				Short	1.333	0.354				
		D8	WT	Long	2.333	0.520		-1.620	0.105	
				Short	1.500	0.289				
		D9	WT	Long	2.056	0.530		-2.132	0.033	*
				Long	1.222	0.364				
		D10	WT	Short	2.056	0.523		-1.289	0.197	
				Long	1.722	0.560				
		D11	WT	Short	2.111	0.594		0.000	1.000	
				Long	2.111	0.633				
		D12	WT	Short	2.167	0.672		-1.527	0.127	
				Long	1.500	0.354				
		D13	WT	Short	1.833	0.577		-1.279	0.201	
				Long	1.333	0.363				
		D14	WT	Short	2.167	0.707		-0.319	0.750	
				Long	1.667	0.408				
		D15	WT	Short	2.111	0.790		-1.614	0.106	
				Long	1.111	0.351				
		D16	WT	Short	1.722	0.619		-0.085	0.932	
				Long	1.611	0.341				

4_2

HE frequency in 0–10 s window

Table	Related Figure	Day	Condition	Group	Mean	SEM	Stastistical test	Z	P	*
Table S4	Figure S4	D0	Long	KO	0.228	0.090	Mann-Whitney U Test	-1.683	0.113	
				WT	0.072	0.032				
		D1	Long	KO	0.194	0.063		-0.135	0.931	
				WT	0.189	0.077				
		D2	Long	KO	0.328	0.066		-0.535	0.605	
				WT	0.300	0.110				
		D3	Long	KO	0.544	0.161		-0.134	0.931	
				WT	0.578	0.212				
		D4	Long	KO	0.822	0.145		-0.975	0.340	
				WT	1.133	0.271				
		D5	Long	KO	1.033	0.190		-0.177	0.863	
				WT	1.144	0.351				
		D6	Long	KO	1.333	0.157		-2.083	0.040	*
				WT	0.811	0.174				
		D7	Long	KO	1.356	0.263		-0.843	0.436	
				WT	1.089	0.199				
		D8	Long	KO	2.100	0.299		-1.864	0.063	
				WT	1.333	0.237				
		D9	Long	KO	2.378	0.308		-2.035	0.040	*
				WT	1.444	0.343				
		D10	Long	KO	1.933	0.317		-0.666	0.546	
				WT	1.467	0.315				
		D11	Long	KO	1.833	0.326		-0.796	0.436	
				WT	1.522	0.386				
		D12	Long	KO	1.856	0.185		-0.798	0.436	
				WT	1.611	0.294				
		D13	Long	KO	1.878	0.258		-1.194	0.258	
				WT	1.411	0.249				
		D14	Long	KO	1.811	0.201		-0.619	0.546	
				WT	1.600	0.292				
		D15	Long	KO	1.944	0.313		-1.694	0.094	
				WT	1.378	0.202				
		D16	Long	KO	1.556	0.146		-0.089	0.931	
				WT	1.433	0.244				
Table S4	Figure S4	D0	Short	KO	0.072	0.033	Mann-Whitney U Test	-0.957	0.436	
				WT	0.067	0.044				
		D1	Short	KO	0.117	0.053		-1.610	0.161	
				WT	0.044	0.034				
		D2	Short	KO	0.122	0.043		-0.725	0.489	
				WT	0.094	0.044				
		D3	Short	KO	0.211	0.081		-0.091	0.931	
				WT	0.333	0.157				
		D4	Short	KO	0.289	0.073		-0.404	0.730	
				WT	0.289	0.065				
		D5	Short	KO	0.267	0.053		-1.066	0.340	
				WT	0.456	0.236				
		D6	Short	KO	0.378	0.144		-1.033	0.340	
				WT	0.256	0.106				
		D7	Short	KO	0.444	0.145		-0.925	0.387	
				WT	0.444	0.211				
		D8	Short	KO	0.500	0.267		-0.448	0.666	
				WT	0.444	0.147				
		D9	Short	KO	0.611	0.291		-1.539	0.136	
				WT	0.367	0.163				
		D10	Short	KO	0.478	0.149		-0.850	0.436	
				WT	0.422	0.153				
		D11	Short	KO	0.589	0.323		-0.401	0.730	
				WT	0.344	0.073				
		D12	Short	KO	0.456	0.082		-0.762	0.489	
				WT	0.389	0.118				
		D13	Short	KO	0.467	0.113		-1.335	0.190	
				WT	0.333	0.120				
		D14	Short	KO	0.567	0.118		-1.377	0.190	
				WT	0.389	0.149				
		D15	Short	KO	0.511	0.098		-1.066	0.297	
				WT	0.378	0.136				
		D16	Short	KO	0.522	0.120		-1.065	0.297	
				WT	0.344	0.091				

HE frequency in 0–10 s window

Table	Related Figure	Day	Group	Condition	Mean	SEM	Stastistical test	Z	P	*
Table S4	Figure S4	D0	KO	Long	0.228	0.090	Wilcoxon Signed Ranks Test	-1.616	0.106	
				Short	0.072	0.033				
		D1	KO	Long	0.194	0.063		-0.931	0.352	
				Short	0.117	0.053				
		D2	KO	Long	0.328	0.066		-2.079	0.038	*
				Short	0.122	0.043				
		D3	KO	Long	0.544	0.161		-1.825	0.068	
				Short	0.211	0.081				
		D4	KO	Long	0.822	0.145		-2.547	0.011	*
				Short	0.289	0.073				
		D5	KO	Long	1.033	0.190		-2.680	0.007	**
				Short	0.267	0.053				
		D6	KO	Long	1.333	0.157		-2.547	0.011	*
				Short	0.378	0.144				
		D7	KO	Long	1.356	0.263		-2.521	0.012	*
				Short	0.444	0.145				
		D8	KO	Long	2.100	0.299		-2.666	0.008	**
				Short	0.500	0.267				
		D9	KO	Long	2.378	0.308		-2.668	0.008	**
				Long	0.611	0.291				
		D10	KO	Short	1.933	0.317		-2.666	0.008	**
				Long	0.478	0.149				
		D11	KO	Short	1.833	0.326		-2.194	0.028	*
				Long	0.589	0.323				
		D12	KO	Short	1.856	0.185		-2.668	0.008	**
				Long	0.456	0.082				
		D13	KO	Short	1.878	0.258		-2.668	0.008	**
				Long	0.467	0.113				
		D14	KO	Short	1.811	0.201		-2.670	0.008	**
				Long	0.567	0.118				
		D15	KO	Short	1.944	0.313		-2.675	0.007	**
				Long	0.511	0.098				
		D16	KO	Short	1.556	0.146		-2.670	0.008	**
				Long	0.522	0.120				
Table S4	Figure S4	D0	WT	Long	0.072	0.032	Wilcoxon Signed Ranks Test	-0.368	0.713	
				Short	0.067	0.044				
		D1	WT	Long	0.189	0.077		-2.379	0.017	*
				Short	0.044	0.034				
		D2	WT	Long	0.300	0.110		-1.753	0.080	
				Short	0.094	0.044				
		D3	WT	Long	0.578	0.212		-1.529	0.126	
				Short	0.333	0.157				
		D4	WT	Long	1.133	0.271		-2.670	0.008	**
				Short	0.289	0.065				
		D5	WT	Long	1.144	0.351		-2.371	0.018	*
				Short	0.456	0.236				
		D6	WT	Long	0.811	0.174		-2.203	0.028	*
				Short	0.256	0.106				
		D7	WT	Long	1.089	0.199		-1.666	0.096	
				Short	0.444	0.211				
		D8	WT	Long	1.333	0.237		-2.561	0.010	*
				Short	0.444	0.147				
		D9	WT	Long	1.444	0.343		-2.439	0.015	*
				Long	0.367	0.163				
		D10	WT	Short	1.467	0.315		-1.904	0.057	
				Long	0.422	0.153				
		D11	WT	Short	1.522	0.386		-2.194	0.028	*
				Long	0.344	0.073				
		D12	WT	Short	1.611	0.294		-2.547	0.011	*
				Long	0.389	0.118				
		D13	WT	Short	1.411	0.249		-2.439	0.015	*
				Long	0.333	0.120				
		D14	WT	Short	1.600	0.292		-2.547	0.011	*
				Long	0.389	0.149				
		D15	WT	Short	1.378	0.202		-2.201	0.028	*
				Long	0.378	0.136				
		D16	WT	Short	1.433	0.244		-2.558	0.011	*
				Long	0.344	0.091				

Supplementary Table 4. HE frequency for 2-s and 10-s latency windows. Differences between groups and conditions were analyzed separately using the Mann–Whitney U test and Wilcoxon signed-rank test. *p < 0.05, **p < 0.01 (N = 9). Abbreviations: HE, head entry; D, training day; KO, CRBN KO mice; WT, wild-type mice.

5_1

HE frequency in 0–1 s window

Table	Related Figure	Day	Condition	Group	Mean	SEM	Stastistical test	Z	P	*
Table S5	Figure 1H,I / Figure S7	D0	Long	KO	0.056	0.056	Mann-Whitney U Test	-1.215	0.387	
				WT	0.278	0.147				
		D1	Long	KO	0.111	0.073		-0.243	0.863	
				WT	0.222	0.147				
		D2	Long	KO	0.167	0.118		-0.784	0.546	
				WT	0.778	0.434				
		D3	Long	KO	0.556	0.338		-0.160	0.931	
				WT	0.333	0.167				
		D4	Long	KO	0.556	0.444		-1.927	0.077	
				WT	1.111	0.309				
		D5	Long	KO	0.333	0.167		-0.338	0.796	
				WT	0.333	0.236				
		D6	Long	KO	1.222	0.434		-1.385	0.222	
				WT	0.556	0.338				
		D7	Long	KO	0.556	0.294		-0.318	0.796	
				WT	0.333	0.167				
		D8	Long	KO	1.778	0.619		-0.318	0.796	
				WT	1.333	0.408				
		D9	Long	KO	1.222	0.521		-0.140	0.931	
				WT	1.000	0.289				
		D10	Long	KO	0.556	0.176		-1.175	0.297	
				WT	1.222	0.401				
		D11	Long	KO	1.000	0.289		-0.992	0.387	
				WT	0.778	0.434				
		D12	Long	KO	0.333	0.236		-0.449	0.730	
				WT	0.444	0.242				
		D13	Long	KO	1.000	0.553		-0.239	0.863	
				WT	0.778	0.278				
		D14	Long	KO	1.333	0.408		-1.666	0.136	
				WT	0.444	0.242				
		D15	Long	KO	1.000	0.333		-1.467	0.190	
				WT	0.444	0.294				
		D16	Long	KO	1.444	0.580		-1.046	0.340	
				WT	0.556	0.242				
Table S5A	Figure 1H,I / Figure S7	D0	Short	KO	0.111	0.111	Mann-Whitney U Test	-1.000	0.730	
				WT	0.000	0.000				
		D1	Short	KO	0.167	0.118		-1.455	0.436	
				WT	0.000	0.000				
		D2	Short	KO	0.444	0.227		-1.626	0.222	
				WT	0.056	0.056				
		D3	Short	KO	0.556	0.242		0.000	1.000	
				WT	0.556	0.242				
		D4	Short	KO	1.000	0.408		-0.193	0.863	
				WT	0.889	0.261				
		D5	Short	KO	0.778	0.278		-0.579	0.605	
				WT	0.556	0.242				
		D6	Short	KO	0.667	0.373		-0.587	0.605	
				WT	0.778	0.278				
		D7	Short	KO	0.889	0.423		-0.743	0.546	
				WT	0.889	0.200				
		D8	Short	KO	1.111	0.261		-0.191	0.863	
				WT	1.111	0.309				
		D9	Short	KO	1.111	0.484		-2.013	0.094	
				WT	0.111	0.111				
		D10	Short	KO	0.778	0.278		-1.190	0.297	
				WT	0.333	0.167				
		D11	Short	KO	1.111	0.351		-1.414	0.222	
				WT	0.556	0.377				
		D12	Short	KO	1.111	0.351		-0.894	0.436	
				WT	0.667	0.236				
		D13	Short	KO	0.444	0.176		-0.201	0.863	
				WT	0.667	0.333				
		D14	Short	KO	1.111	0.351		-0.940	0.387	
				WT	0.667	0.289				
		D15	Short	KO	1.444	0.580		-0.943	0.387	
				WT	0.556	0.176				
		D16	Short	KO	0.889	0.309		-1.080	0.340	
				WT	0.444	0.242				

HE frequency in 0–1 s window

Table	Related Figure	Day	Group	Condition	Mean	SEM	Stastistical test	Z	P	*
Table S5	Figure 1H,I / Figure S7	D0	KO	Long	0.056	0.056	Wilcoxon Signed Ranks Test	-1.000	0.317	
				Short	0.111	0.111				
		D1	KO	Long	0.111	0.073		-0.577	0.564	
				Short	0.167	0.118				
		D2	KO	Long	0.167	0.118		-1.633	0.102	
				Short	0.444	0.227				
		D3	KO	Long	0.556	0.338		-0.378	0.705	
				Short	0.556	0.242				
		D4	KO	Long	0.556	0.444		-1.081	0.279	
				Short	1.000	0.408				
		D5	KO	Long	0.333	0.167		-1.633	0.102	
				Short	0.778	0.278				
		D6	KO	Long	1.222	0.434		-0.843	0.399	
				Short	0.667	0.373				
		D7	KO	Long	0.556	0.294		-0.647	0.518	
				Short	0.889	0.423				
		D8	KO	Long	1.778	0.619		-1.186	0.236	
				Short	1.111	0.261				
		D9	KO	Long	1.222	0.521		-0.175	0.861	
				Long	1.111	0.484				
		D10	KO	Short	0.556	0.176		-1.000	0.317	
				Long	0.778	0.278				
		D11	KO	Short	1.000	0.289		-0.302	0.763	
				Long	1.111	0.351				
		D12	KO	Short	0.333	0.236		-1.841	0.066	
				Long	1.111	0.351				
		D13	KO	Short	1.000	0.553		-0.647	0.518	
				Long	0.444	0.176				
		D14	KO	Short	1.333	0.408		-0.412	0.680	
				Long	1.111	0.351				
		D15	KO	Short	1.000	0.333		-0.632	0.527	
				Long	1.444	0.580				
		D16	KO	Short	1.444	0.580		-1.289	0.197	
				Long	0.889	0.309				
Table S5	Figure 1H,I / Figure S7	D0	WT	Long	0.278	0.147	Wilcoxon Signed Ranks Test	-1.633	0.102	
				Short	0.000	0.000				
		D1	WT	Long	0.222	0.147		-1.414	0.157	
				Short	0.000	0.000				
		D2	WT	Long	0.778	0.434		-1.473	0.141	
				Short	0.056	0.056				
		D3	WT	Long	0.333	0.167		-1.414	0.157	
				Short	0.556	0.242				
		D4	WT	Long	1.111	0.309		-0.632	0.527	
				Short	0.889	0.261				
		D5	WT	Long	0.333	0.236		-0.541	0.589	
				Short	0.556	0.242				
		D6	WT	Long	0.556	0.338		-0.541	0.589	
				Short	0.778	0.278				
		D7	WT	Long	0.333	0.167		-1.667	0.096	
				Short	0.889	0.200				
		D8	WT	Long	1.333	0.408		-0.574	0.566	
				Short	1.111	0.309				
		D9	WT	Long	1.000	0.289		-2.271	0.023	*
				Long	0.111	0.111				
		D10	WT	Short	1.222	0.401		-1.807	0.071	
				Long	0.333	0.167				
		D11	WT	Short	0.778	0.434		-0.707	0.480	
				Long	0.556	0.377				
		D12	WT	Short	0.444	0.242		-1.000	0.317	
				Long	0.667	0.236				
		D13	WT	Short	0.778	0.278		-0.276	0.783	
				Long	0.667	0.333				
		D14	WT	Short	0.444	0.242		-0.743	0.458	
				Long	0.667	0.289				
		D15	WT	Short	0.444	0.294		-0.176	0.860	
				Long	0.556	0.176				
		D16	WT	Short	0.556	0.242		-0.333	0.739	
				Long	0.444	0.242				

5_2

HE frequency in 1–2 s window

Table	Related Figure	Day	Condition	Group	Mean	SEM	Stastistical test	Z	P	*
Table S5	Figure 1H,I / Figure S7	D0	Long	KO	0.278	0.222	Mann-Whitney U Test	-1.455	0.436	
				WT	0.000	0.000				
		D1	Long	KO	0.056	0.056		-0.081	1.000	
				WT	0.111	0.111				
		D2	Long	KO	0.278	0.147		-0.910	0.546	
				WT	0.444	0.444				
		D3	Long	KO	0.333	0.167		-0.338	0.796	
				WT	0.444	0.338				
		D4	Long	KO	0.889	0.423		-0.143	0.931	
				WT	0.778	0.278				
		D5	Long	KO	1.222	0.324		-0.368	0.730	
				WT	1.222	0.494				
		D6	Long	KO	0.889	0.200		-0.335	0.796	
				WT	1.111	0.351				
		D7	Long	KO	1.667	0.373		-0.809	0.489	
				WT	1.222	0.222				
		D8	Long	KO	1.889	0.539		-0.775	0.489	
				WT	1.333	0.333				
		D9	Long	KO	2.222	0.465		-1.590	0.136	
				WT	1.222	0.324				
		D10	Long	KO	1.556	0.377		-0.183	0.863	
				WT	1.444	0.412				
		D11	Long	KO	2.000	0.986		-0.549	0.605	
				WT	1.556	0.294				
		D12	Long	KO	2.889	0.807		-0.454	0.666	
				WT	2.222	0.572				
		D13	Long	KO	2.667	0.601		-1.769	0.094	
				WT	1.222	0.465				
		D14	Long	KO	1.889	0.512		-0.090	0.931	
				WT	2.000	0.577				
		D15	Long	KO	2.444	0.729		-1.009	0.340	
				WT	1.444	0.530				
		D16	Long	KO	2.111	0.696		-1.262	0.258	
				WT	0.889	0.455				
Table S5A	Figure 1H,I / Figure S7	D0	Short	KO	0.167	0.083	Mann-Whitney U Test	-0.913	0.546	
				WT	0.111	0.111				
		D1	Short	KO	0.444	0.242		-1.837	0.258	
				WT	0.000	0.000				
		D2	Short	KO	0.444	0.155		-0.528	0.666	
				WT	0.333	0.144				
		D3	Short	KO	0.556	0.176		-0.248	0.863	
				WT	0.667	0.236				
		D4	Short	KO	1.222	0.619		-0.876	0.436	
				WT	1.333	0.373				
		D5	Short	KO	0.889	0.200		0.000	1.000	
				WT	1.000	0.333				
		D6	Short	KO	1.222	0.222		-2.466	0.019	*
				WT	0.333	0.236				
		D7	Short	KO	2.111	0.588		-2.233	0.031	*
				WT	0.444	0.242				
		D8	Short	KO	1.778	0.741		-0.369	0.730	
				WT	1.111	0.309				
		D9	Short	KO	2.444	0.475		-2.022	0.050	
				WT	1.222	0.222				
		D10	Short	KO	2.111	0.423		-0.859	0.436	
				WT	1.556	0.503				
		D11	Short	KO	1.444	0.648		-0.773	0.489	
				WT	1.667	0.408				
		D12	Short	KO	1.778	0.572		-0.181	0.863	
				WT	1.444	0.412				
		D13	Short	KO	2.333	0.764		-1.284	0.222	
				WT	0.778	0.222				
		D14	Short	KO	2.667	0.764		-1.219	0.258	
				WT	1.333	0.333				
		D15	Short	KO	2.222	0.830		-1.216	0.258	
				WT	1.111	0.564				
		D16	Short	KO	2.444	0.689		-0.816	0.436	
				WT	1.667	0.373				

HE frequency in 1–2 s window

Table	Related Figure	Day	Group	Condition	Mean	SEM	Stastistical test	Z	P	*
Table S5	Figure 1H,I / Figure S7	D0	KO	Long	0.278	0.222	Wilcoxon Signed Ranks Test	0.000	1.000	
				Short	0.167	0.083				
		D1	KO	Long	0.056	0.056		-1.473	0.141	
				Short	0.444	0.242				
		D2	KO	Long	0.278	0.147		-0.780	0.435	
				Short	0.444	0.155				
		D3	KO	Long	0.333	0.167		-1.000	0.317	
				Short	0.556	0.176				
		D4	KO	Long	0.889	0.423		-0.319	0.750	
				Short	1.222	0.619				
		D5	KO	Long	1.222	0.324		-1.000	0.317	
				Short	0.889	0.200				
		D6	KO	Long	0.889	0.200		-0.966	0.334	
				Short	1.222	0.222				
		D7	KO	Long	1.667	0.373		-0.557	0.577	
				Short	2.111	0.588				
		D8	KO	Long	1.889	0.539		-0.259	0.796	
				Short	1.778	0.741				
		D9	KO	Long	2.222	0.465		-0.541	0.589	
				Long	2.444	0.475				
		D10	KO	Short	1.556	0.377		-1.186	0.236	
				Long	2.111	0.423				
		D11	KO	Short	2.000	0.986		-1.342	0.180	
				Long	1.444	0.648				
		D12	KO	Short	2.889	0.807		-1.558	0.119	
				Long	1.778	0.572				
		D13	KO	Short	2.667	0.601		-0.638	0.524	
				Long	2.333	0.764				
		D14	KO	Short	1.889	0.512		-0.938	0.348	
				Long	2.667	0.764				
		D15	KO	Short	2.444	0.729		-0.406	0.684	
				Long	2.222	0.830				
		D16	KO	Short	2.111	0.696		-0.722	0.470	
				Long	2.444	0.689				
Table S5	Figure 1H,I / Figure S7	D0	WT	Long	0.000	0.000	Wilcoxon Signed Ranks Test	-1.000	0.317	
				Short	0.111	0.111				
		D1	WT	Long	0.111	0.111		-1.000	0.317	
				Short	0.000	0.000				
		D2	WT	Long	0.444	0.444		-0.680	0.496	
				Short	0.333	0.144				
		D3	WT	Long	0.444	0.338		-1.000	0.317	
				Short	0.667	0.236				
		D4	WT	Long	0.778	0.278		-1.667	0.096	
				Short	1.333	0.373				
		D5	WT	Long	1.222	0.494		-0.552	0.581	
				Short	1.000	0.333				
		D6	WT	Long	1.111	0.351		-1.933	0.053	
				Short	0.333	0.236				
		D7	WT	Long	1.222	0.222		-1.823	0.068	
				Short	0.444	0.242				
		D8	WT	Long	1.333	0.333		-0.632	0.527	
				Short	1.111	0.309				
		D9	WT	Long	1.222	0.324		0.000	1.000	
				Long	1.222	0.222				
		D10	WT	Short	1.444	0.412		-0.447	0.655	
				Long	1.556	0.503				
		D11	WT	Short	1.556	0.294		-0.431	0.666	
				Long	1.667	0.408				
		D12	WT	Short	2.222	0.572		-1.552	0.121	
				Long	1.444	0.412				
		D13	WT	Short	1.222	0.465		-1.134	0.257	
				Long	0.778	0.222				
		D14	WT	Short	2.000	0.577		-1.382	0.167	
				Long	1.333	0.333				
		D15	WT	Short	1.444	0.530		-0.414	0.679	
				Long	1.111	0.564				
		D16	WT	Short	0.889	0.455		-1.890	0.059	
				Long	1.667	0.373				

5_3

HE frequency in 2–3 s window

Table	Related Figure	Day	Condition	Group	Mean	SEM	Stastistical test	Z	P	*
Table S5	Figure 1H,I / Figure S7	D0	Long	KO	0.333	0.220	Mann-Whitney U Test	-1.837	0.258	
				WT	0.000	0.000				
		D1	Long	KO	0.500	0.236		-1.514	0.258	
				WT	0.111	0.111				
		D2	Long	KO	0.278	0.147		-1.035	0.489	
				WT	0.444	0.444				
		D3	Long	KO	0.778	0.434		-0.458	0.730	
				WT	0.444	0.338				
		D4	Long	KO	0.444	0.242		-0.843	0.489	
				WT	0.778	0.278				
		D5	Long	KO	0.778	0.434		-0.530	0.666	
				WT	1.222	0.494				
		D6	Long	KO	1.333	0.408		-0.279	0.796	
				WT	1.111	0.351				
		D7	Long	KO	2.222	0.662		-1.274	0.222	
				WT	1.222	0.222				
		D8	Long	KO	1.889	0.735		-0.273	0.796	
				WT	1.333	0.333				
		D9	Long	KO	2.667	0.553		-0.365	0.730	
				WT	1.222	0.324				
		D10	Long	KO	2.333	0.833		-0.045	1.000	
				WT	1.444	0.412				
		D11	Long	KO	1.778	0.760		-0.635	0.546	
				WT	1.556	0.294				
		D12	Long	KO	2.333	0.624		-0.855	0.436	
				WT	2.222	0.572				
		D13	Long	KO	2.667	0.782		-0.637	0.546	
				WT	1.222	0.465				
		D14	Long	KO	2.556	0.503		-0.093	0.931	
				WT	2.000	0.577				
		D15	Long	KO	2.778	0.760		-0.454	0.666	
				WT	1.444	0.530				
		D16	Long	KO	2.667	0.833		-0.408	0.730	
				WT	0.889	0.455				
Table S5A	Figure 1H,I / Figure S7	D0	Short	KO	0.263	0.088	Mann-Whitney U Test	0.000	1.000	
				WT	0.000	0.000				
		D1	Short	KO	1.120	0.456		-2.557	0.050	
				WT	0.000	0.000				
		D2	Short	KO	0.371	0.103		-1.389	0.222	
				WT	0.222	0.121				
		D3	Short	KO	0.751	0.149		-0.491	0.666	
				WT	0.667	0.373				
		D4	Short	KO	0.420	0.061		-0.356	0.796	
				WT	0.556	0.338				
		D5	Short	KO	0.961	0.136		-0.681	0.546	
				WT	1.000	0.601				
		D6	Short	KO	0.641	0.154		-1.459	0.222	
				WT	0.444	0.294				
		D7	Short	KO	1.179	0.313		-0.793	0.489	
				WT	1.444	0.338				
		D8	Short	KO	1.258	0.305		-1.173	0.258	
				WT	1.667	0.441				
		D9	Short	KO	1.528	0.452		-1.199	0.258	
				WT	1.444	0.338				
		D10	Short	KO	1.422	0.344		-0.045	1.000	
				WT	1.444	0.294				
		D11	Short	KO	1.330	0.335		-0.135	0.931	
				WT	2.111	0.539				
		D12	Short	KO	1.406	0.322		-0.413	0.730	
				WT	1.556	0.444				
		D13	Short	KO	1.542	0.400		-0.273	0.796	
				WT	1.889	0.261				
		D14	Short	KO	1.345	0.475		-1.944	0.063	
				WT	0.778	0.324				
		D15	Short	KO	1.507	0.422		-0.972	0.387	
				WT	1.111	0.351				
		D16	Short	KO	1.499	0.399		-0.772	0.489	
				WT	1.556	0.338				

HE frequency in 2–3 s window

Table	Related Figure	Day	Group	Condition	Mean	SEM	Stastistical test	Z	P	*
Table S5	Figure 1H,I / Figure S7	D0	KO	Long	0.333	0.220	Wilcoxon Signed Ranks Test	-1.633	0.102	
				Short	0.263	0.088				
		D1	KO	Long	0.500	0.236		-0.216	0.829	
				Short	1.120	0.456				
		D2	KO	Long	0.278	0.147		-1.633	0.102	
				Short	0.371	0.103				
		D3	KO	Long	0.778	0.434		0.000	1.000	
				Short	0.751	0.149				
		D4	KO	Long	0.444	0.242		-0.333	0.739	
				Short	0.420	0.061				
		D5	KO	Long	0.778	0.434		-0.962	0.336	
				Short	0.961	0.136				
		D6	KO	Long	1.333	0.408		-0.213	0.832	
				Short	0.641	0.154				
		D7	KO	Long	2.222	0.662		-1.586	0.113	
				Short	1.179	0.313				
		D8	KO	Long	1.889	0.735		-0.954	0.340	
				Short	1.258	0.305				
		D9	KO	Long	2.667	0.553		-1.127	0.260	
				Long	1.528	0.452				
		D10	KO	Short	2.333	0.833		-0.862	0.389	
				Long	1.422	0.344				
		D11	KO	Short	1.778	0.760		-0.816	0.414	
				Long	1.330	0.335				
		D12	KO	Short	2.333	0.624		-0.531	0.595	
				Long	1.406	0.322				
		D13	KO	Short	2.667	0.782		-1.194	0.233	
				Long	1.542	0.400				
		D14	KO	Short	2.556	0.503		-1.294	0.196	
				Long	1.345	0.475				
		D15	KO	Short	2.778	0.760		-1.802	0.072	
				Long	1.507	0.422				
		D16	KO	Short	2.667	0.833		-1.023	0.306	
				Long	1.499	0.399				
Table S5	Figure 1H,I / Figure S7	D0	WT	Long	0.000	0.000	Wilcoxon Signed Ranks Test	0.000	1.000	
				Short	0.000	0.000				
		D1	WT	Long	0.111	0.111		-1.000	0.317	
				Short	0.000	0.000				
		D2	WT	Long	0.444	0.444		-1.414	0.157	
				Short	0.222	0.121				
		D3	WT	Long	0.444	0.338		-0.552	0.581	
				Short	0.667	0.373				
		D4	WT	Long	0.778	0.278		-0.541	0.589	
				Short	0.556	0.338				
		D5	WT	Long	1.222	0.494		-0.085	0.932	
				Short	1.000	0.601				
		D6	WT	Long	1.111	0.351		-1.633	0.102	
				Short	0.444	0.294				
		D7	WT	Long	1.222	0.222		-0.965	0.335	
				Short	1.444	0.338				
		D8	WT	Long	1.333	0.333		-0.072	0.943	
				Short	1.667	0.441				
		D9	WT	Long	1.222	0.324		-1.310	0.190	
				Long	1.444	0.338				
		D10	WT	Short	1.444	0.412		-0.791	0.429	
				Long	1.444	0.294				
		D11	WT	Short	1.556	0.294		-0.568	0.570	
				Long	2.111	0.539				
		D12	WT	Short	2.222	0.572		-0.359	0.719	
				Long	1.556	0.444				
		D13	WT	Short	1.222	0.465		0.000	1.000	
				Long	1.889	0.261				
		D14	WT	Short	2.000	0.577		-2.036	0.042	*
				Long	0.778	0.324				
		D15	WT	Short	1.444	0.530		-1.549	0.121	
				Long	1.111	0.351				
		D16	WT	Short	0.889	0.455		-1.134	0.257	
				Long	1.556	0.338				

Supplementary Table 5. HE frequency for 1-s latency windows. Differences between groups and conditions for individual windows were analyzed separately using the Mann–Whitney U test and Wilcoxon signed-rank test. *p < 0.05, **p < 0.01 (N = 9). Abbreviations: HE, head entry; D, training day; KO, CRBN KO mice; WT, wild-type mice.

5_4

Table	Related Figure	Day	Condition	Group	Mean	SEM	Stastistical test	Z	P	*
HE frequency in 3–4 s window										
Table S5	Figure 1H,I / Figure S7	D0	Long	KO	0.444	0.212	Mann-Whitney U Test	-2.187	0.113	
				WT	0.000	0.000				
		D1	Long	KO	0.167	0.118		0.000	1.000	
				WT	0.167	0.118				
		D2	Long	KO	0.278	0.147		-1.215	0.387	
				WT	0.056	0.056				
		D3	Long	KO	0.556	0.242		-1.578	0.222	
				WT	0.111	0.111				
		D4	Long	KO	1.000	0.373		-0.372	0.730	
				WT	1.111	0.309				
		D5	Long	KO	0.667	0.289		-0.293	0.796	
				WT	1.000	0.471				
		D6	Long	KO	1.556	0.503		-1.269	0.258	
				WT	0.778	0.222				
		D7	Long	KO	1.333	0.645		-0.047	1.000	
				WT	0.889	0.351				
		D8	Long	KO	2.222	0.741		-0.454	0.666	
				WT	1.667	0.333				
		D9	Long	KO	3.111	0.935		-0.761	0.489	
				WT	2.000	0.577				
		D10	Long	KO	2.889	0.992		-0.227	0.863	
				WT	1.778	0.401				
		D11	Long	KO	3.000	1.067		-0.588	0.605	
				WT	2.111	0.633				
		D12	Long	KO	1.667	0.408		-0.281	0.796	
				WT	1.889	0.200				
		D13	Long	KO	2.444	0.626		-0.270	0.796	
				WT	2.222	0.703				
		D14	Long	KO	2.444	0.503		-0.988	0.340	
				WT	1.889	0.655				
		D15	Long	KO	2.556	0.530		-1.078	0.297	
				WT	1.889	0.676				
		D16	Long	KO	1.111	0.309		-1.365	0.190	
				WT	2.222	0.619				
Table S5A	Figure 1H,I / Figure S7	D0	Short	KO	0.167	0.083	Mann-Whitney U Test	-1.844	0.258	
				WT	0.000	0.000				
		D1	Short	KO	0.556	0.227		-1.847	0.136	
				WT	0.111	0.111				
		D2	Short	KO	0.444	0.242		-0.403	0.730	
				WT	0.556	0.256				
		D3	Short	KO	0.556	0.242		-0.826	0.489	
				WT	0.889	0.309				
		D4	Short	KO	1.222	0.364		-1.363	0.222	
				WT	0.556	0.242				
		D5	Short	KO	1.444	0.444		-0.046	1.000	
				WT	1.556	0.530				
		D6	Short	KO	1.000	0.236		-0.328	0.796	
				WT	0.889	0.309				
		D7	Short	KO	2.000	0.500		-0.870	0.436	
				WT	1.444	0.444				
		D8	Short	KO	2.111	0.455		-2.332	0.031	*
				WT	0.778	0.324				
		D9	Short	KO	2.222	0.521		-0.499	0.666	
				WT	1.889	0.655				
		D10	Short	KO	1.889	0.455		-0.368	0.730	
				WT	1.667	0.373				
		D11	Short	KO	1.556	0.530		-0.092	0.931	
				WT	1.333	0.236				
		D12	Short	KO	1.556	0.444		-0.046	1.000	
				WT	1.556	0.475				
		D13	Short	KO	1.333	0.333		-0.458	0.666	
				WT	1.556	0.377				
		D14	Short	KO	1.222	0.434		-0.787	0.489	
				WT	1.556	0.377				
		D15	Short	KO	1.111	0.351		-0.731	0.489	
				WT	1.778	0.572				
		D16	Short	KO	1.556	0.530		0.000	1.000	
				WT	1.444	0.412				

Table	Related Figure	Day	Group	Condition	Mean	SEM	Stastistical test	Z	P	*
HE frequency in 3–4 s window										
Table S5	Figure 1H,I / Figure S7	D0	KO	Long	0.444	0.212	Wilcoxon Signed Ranks Test	-1.186	0.236	
				Short	0.167	0.083				
		D1	KO	Long	0.167	0.118		-1.604	0.109	
				Short	0.556	0.227				
		D2	KO	Long	0.278	0.147		-1.089	0.276	
				Short	0.444	0.242				
		D3	KO	Long	0.556	0.242		0.000	1.000	
				Short	0.556	0.242				
		D4	KO	Long	1.000	0.373		-0.333	0.739	
				Short	1.222	0.364				
		D5	KO	Long	0.667	0.289		-1.403	0.161	
				Short	1.444	0.444				
		D6	KO	Long	1.556	0.503		-1.265	0.206	
				Short	1.000	0.236				
		D7	KO	Long	1.333	0.645		-1.150	0.250	
				Short	2.000	0.500				
		D8	KO	Long	2.222	0.741		-0.284	0.776	
				Short	2.111	0.455				
		D9	KO	Long	3.111	0.935		-1.200	0.230	
				Long	2.222	0.521				
		D10	KO	Short	2.889	0.992		-1.109	0.268	
				Long	1.889	0.455				
		D11	KO	Short	3.000	1.067		-1.378	0.168	
				Long	1.556	0.530				
		D12	KO	Short	1.667	0.408		-0.447	0.655	
				Long	1.556	0.444				
		D13	KO	Short	2.444	0.626		-1.556	0.120	
				Long	1.333	0.333				
		D14	KO	Short	2.444	0.503		-1.620	0.105	
				Long	1.222	0.434				
		D15	KO	Short	2.556	0.530		-1.982	0.047	*
				Long	1.111	0.351				
		D16	KO	Short	1.111	0.309		-0.744	0.457	
				Long	1.556	0.530				
Table S5	Figure 1H,I / Figure S7	D0	WT	Long	0.000	0.000	Wilcoxon Signed Ranks Test	0.000	1.000	
				Short	0.000	0.000				
		D1	WT	Long	0.167	0.118		-0.272	0.785	
				Short	0.111	0.111				
		D2	WT	Long	0.056	0.056		-1.841	0.066	
				Short	0.556	0.256				
		D3	WT	Long	0.111	0.111		-2.333	0.020	*
				Short	0.889	0.309				
		D4	WT	Long	1.111	0.309		-2.236	0.025	*
				Short	0.556	0.242				
		D5	WT	Long	1.000	0.471		-0.712	0.476	
				Short	1.556	0.530				
		D6	WT	Long	0.778	0.222		-0.333	0.739	
				Short	0.889	0.309				
		D7	WT	Long	0.889	0.351		-1.035	0.301	
				Short	1.444	0.444				
		D8	WT	Long	1.667	0.333		-1.282	0.200	
				Short	0.778	0.324				
		D9	WT	Long	2.000	0.577		-0.264	0.792	
				Long	1.889	0.655				
		D10	WT	Short	1.778	0.401		-0.289	0.773	
				Long	1.667	0.373				
		D11	WT	Short	2.111	0.633		-1.380	0.168	
				Long	1.333	0.236				
		D12	WT	Short	1.889	0.200		-0.548	0.584	
				Long	1.556	0.475				
		D13	WT	Short	2.222	0.703		-0.736	0.462	
				Long	1.556	0.377				
		D14	WT	Short	1.889	0.655		-0.283	0.777	
				Long	1.556	0.377				
		D15	WT	Short	1.889	0.676		-0.512	0.609	
				Long	1.778	0.572				
		D16	WT	Short	2.222	0.619		-0.718	0.473	
				Long	1.444	0.412				

5_5

Table	Related Figure	Day	Condition	Group	Mean	SEM	Stastistical test	Z	P	*
HE frequency in 4–5 s window										
Table S5	Figure 1H,I / Figure S7	D0	Long	KO	0.111	0.073	Mann-Whitney U Test	-1.458	0.436	
				WT	0.000	0.000				
		D1	Long	KO	0.167	0.118		0.000	1.000	
				WT	0.167	0.118				
		D2	Long	KO	0.389	0.232		-0.616	0.666	
				WT	0.167	0.118				
		D3	Long	KO	0.778	0.364		-0.147	0.931	
				WT	0.667	0.333				
		D4	Long	KO	1.000	0.441		-0.648	0.546	
				WT	1.222	0.364				
		D5	Long	KO	0.444	0.242		-1.719	0.113	
				WT	1.222	0.401				
		D6	Long	KO	1.333	0.441		-0.839	0.436	
				WT	0.889	0.423				
		D7	Long	KO	1.444	0.377		-0.415	0.730	
				WT	1.222	0.324				
		D8	Long	KO	2.111	0.539		-1.300	0.222	
				WT	1.222	0.364				
		D9	Long	KO	2.889	0.633		-1.388	0.190	
				WT	1.667	0.624				
		D10	Long	KO	2.444	0.818		-0.274	0.796	
				WT	1.667	0.471				
		D11	Long	KO	2.667	0.667		-0.813	0.436	
				WT	2.000	0.333				
		D12	Long	KO	1.556	0.412		-0.913	0.387	
				WT	2.556	0.766				
		D13	Long	KO	2.000	0.850		-0.953	0.387	
				WT	2.444	0.444				
		D14	Long	KO	1.111	0.309		-2.137	0.040	*
				WT	2.778	0.662				
		D15	Long	KO	1.333	0.289		-2.320	0.024	*
				WT	2.778	0.521				
		D16	Long	KO	1.111	0.309		-1.799	0.094	
				WT	2.333	0.527				
Table S5A	Figure 1H,I / Figure S7	D0	Short	KO	0.222	0.121	Mann-Whitney U Test	-0.318	0.796	
				WT	0.333	0.167				
		D1	Short	KO	0.111	0.111		0.000	1.000	
				WT	0.111	0.111				
		D2	Short	KO	0.111	0.111		-1.035	0.489	
				WT	0.278	0.147				
		D3	Short	KO	0.778	0.324		-1.060	0.387	
				WT	0.333	0.236				
		D4	Short	KO	1.111	0.309		-1.585	0.161	
				WT	0.444	0.242				
		D5	Short	KO	1.222	0.324		-0.515	0.666	
				WT	1.111	0.423				
		D6	Short	KO	1.556	0.377		-0.229	0.863	
				WT	1.444	0.412				
		D7	Short	KO	0.889	0.455		-0.382	0.730	
				WT	0.889	0.309				
		D8	Short	KO	1.111	0.389		0.000	1.000	
				WT	1.111	0.389				
		D9	Short	KO	0.667	0.289		-1.617	0.136	
				WT	1.556	0.412				
		D10	Short	KO	1.222	0.364		-0.686	0.546	
				WT	1.778	0.521				
		D11	Short	KO	1.222	0.547		-0.689	0.546	
				WT	1.333	0.333				
		D12	Short	KO	0.333	0.167		-1.738	0.113	
				WT	1.000	0.289				
		D13	Short	KO	1.556	0.412		-1.781	0.094	
				WT	0.556	0.242				
		D14	Short	KO	1.111	0.633		-0.639	0.605	
				WT	0.556	0.294				
		D15	Short	KO	0.556	0.242		-0.407	0.730	
				WT	0.444	0.242				
		D16	Short	KO	0.556	0.176		-0.653	0.605	
				WT	0.444	0.242				

Table	Related Figure	Day	Group	Condition	Mean	SEM	Stastistical test	Z	P	*
HE frequency in 4–5 s window										
Table S5	Figure 1H,I / Figure S7	D0	KO	Long	0.111	0.073	Wilcoxon Signed Ranks Test	-0.816	0.414	
				Short	0.222	0.121				
		D1	KO	Long	0.167	0.118		-1.000	0.317	
				Short	0.111	0.111				
		D2	KO	Long	0.389	0.232		-0.921	0.357	
				Short	0.111	0.111				
		D3	KO	Long	0.778	0.364		0.000	1.000	
				Short	0.778	0.324				
		D4	KO	Long	1.000	0.441		-0.276	0.783	
				Short	1.111	0.309				
		D5	KO	Long	0.444	0.242		-1.588	0.112	
				Short	1.222	0.324				
		D6	KO	Long	1.333	0.441		-0.439	0.660	
				Short	1.556	0.377				
		D7	KO	Long	1.444	0.377		-1.179	0.238	
				Short	0.889	0.455				
		D8	KO	Long	2.111	0.539		-1.562	0.118	
				Short	1.111	0.389				
		D9	KO	Long	2.889	0.633		-2.207	0.027	*
				Long	0.667	0.289				
		D10	KO	Short	2.444	0.818		-1.380	0.168	
				Long	1.222	0.364				
		D11	KO	Short	2.667	0.667		-1.370	0.171	
				Long	1.222	0.547				
		D12	KO	Short	1.556	0.412		-1.994	0.046	*
				Long	0.333	0.167				
		D13	KO	Short	2.000	0.850		-0.426	0.670	
				Long	1.556	0.412				
		D14	KO	Short	1.111	0.309		-0.531	0.595	
				Long	1.111	0.633				
		D15	KO	Short	1.333	0.289		-1.552	0.121	
				Long	0.556	0.242				
		D16	KO	Short	1.111	0.309		-1.406	0.160	
				Long	0.556	0.176				
Table S5	Figure 1H,I / Figure S7	D0	WT	Long	0.000	0.000	Wilcoxon Signed Ranks Test	-1.732	0.083	
				Short	0.333	0.167				
		D1	WT	Long	0.167	0.118		-0.272	0.785	
				Short	0.111	0.111				
		D2	WT	Long	0.167	0.118		-0.552	0.581	
				Short	0.278	0.147				
		D3	WT	Long	0.667	0.333		-0.707	0.480	
				Short	0.333	0.236				
		D4	WT	Long	1.222	0.364		-1.552	0.121	
				Short	0.444	0.242				
		D5	WT	Long	1.222	0.401		-0.108	0.914	
				Short	1.111	0.423				
		D6	WT	Long	0.889	0.423		-1.179	0.238	
				Short	1.444	0.412				
		D7	WT	Long	1.222	0.324		-0.680	0.496	
				Short	0.889	0.309				
		D8	WT	Long	1.222	0.364		-0.264	0.792	
				Short	1.111	0.389				
		D9	WT	Long	1.667	0.624		-0.141	0.888	
				Long	1.556	0.412				
		D10	WT	Short	1.667	0.471		-0.086	0.931	
				Long	1.778	0.521				
		D11	WT	Short	2.000	0.333		-1.121	0.262	
				Long	1.333	0.333				
		D12	WT	Short	2.556	0.766		-1.693	0.090	
				Long	1.000	0.289				
		D13	WT	Short	2.444	0.444		-2.530	0.011	*
				Long	0.556	0.242				
		D14	WT	Short	2.778	0.662		-2.536	0.011	*
				Long	0.556	0.294				
		D15	WT	Short	2.778	0.521		-2.694	0.007	**
				Long	0.444	0.242				
		D16	WT	Short	2.333	0.527		-2.116	0.034	*
				Long	0.444	0.242				

5_6

Table	Related Figure	Day	Condition	Group	Mean	SEM	Stastistical test	Z	P	*
HE frequency in 5–6 s window										
Table S5	Figure 1H,I / Figure S7	D0	Long	KO	0.333	0.144	Mann-Whitney U Test	-2.187	0.113	
				WT	0.000	0.000				
		D1	Long	KO	0.167	0.118		-0.449	0.730	
				WT	0.222	0.121				
		D2	Long	KO	0.111	0.073		-0.243	0.863	
				WT	0.278	0.188				
		D3	Long	KO	0.556	0.294		-0.687	0.546	
				WT	0.778	0.278				
		D4	Long	KO	1.556	0.475		-1.405	0.190	
				WT	0.667	0.289				
		D5	Long	KO	0.889	0.423		-0.430	0.730	
				WT	1.111	0.423				
		D6	Long	KO	1.222	0.364		-0.417	0.730	
				WT	1.000	0.289				
		D7	Long	KO	0.667	0.333		-1.535	0.161	
				WT	2.111	0.696				
		D8	Long	KO	2.222	0.572		-0.882	0.436	
				WT	1.556	0.412				
		D9	Long	KO	2.778	0.778		-0.769	0.489	
				WT	1.889	0.455				
		D10	Long	KO	2.222	0.572		-0.180	0.863	
				WT	2.111	0.564				
		D11	Long	KO	2.333	0.471		-0.682	0.546	
				WT	1.889	0.484				
		D12	Long	KO	1.667	0.408		-0.092	0.931	
				WT	1.667	0.471				
		D13	Long	KO	2.000	0.441		-0.662	0.546	
				WT	1.778	0.641				
		D14	Long	KO	1.333	0.471		-1.000	0.340	
				WT	1.889	0.423				
		D15	Long	KO	2.000	0.667		-0.639	0.546	
				WT	1.333	0.408				
		D16	Long	KO	1.333	0.441		-0.184	0.863	
				WT	1.444	0.626				
Table S5A	Figure 1H,I / Figure S7	D0	Short	KO	0.056	0.056	Mann-Whitney U Test	-1.627	0.222	
				WT	0.333	0.144				
		D1	Short	KO	0.278	0.147		0.000	1.000	
				WT	0.278	0.147				
		D2	Short	KO	0.500	0.204		-0.911	0.436	
				WT	0.222	0.121				
		D3	Short	KO	0.556	0.338		-0.264	0.863	
				WT	0.889	0.455				
		D4	Short	KO	0.444	0.176		-1.380	0.222	
				WT	1.444	0.475				
		D5	Short	KO	0.778	0.278		-1.101	0.340	
				WT	1.222	0.278				
		D6	Short	KO	1.111	0.423		-1.669	0.136	
				WT	0.444	0.338				
		D7	Short	KO	1.333	0.236		-0.047	1.000	
				WT	1.333	0.333				
		D8	Short	KO	1.667	0.707		-0.232	0.863	
				WT	0.889	0.261				
		D9	Short	KO	0.778	0.278		0.000	1.000	
				WT	0.778	0.278				
		D10	Short	KO	1.444	0.475		-0.552	0.605	
				WT	1.000	0.289				
		D11	Short	KO	0.889	0.389		-0.949	0.436	
				WT	0.444	0.338				
		D12	Short	KO	1.222	0.364		-0.233	0.863	
				WT	1.333	0.373				
		D13	Short	KO	0.778	0.278		-0.687	0.546	
				WT	0.556	0.294				
		D14	Short	KO	0.778	0.222		-2.004	0.063	
				WT	1.889	0.423				
		D15	Short	KO	0.889	0.351		-0.191	0.863	
				WT	0.778	0.324				
		D16	Short	KO	0.556	0.176		-0.653	0.605	
				WT	0.444	0.242				

Table	Related Figure	Day	Group	Condition	Mean	SEM	Stastistical test	Z	P	*
HE frequency in 5–6 s window										
Table S5	Figure 1H,I / Figure S7	D0	KO	Long	0.333	0.144	Wilcoxon Signed Ranks Test	-1.890	0.059	
				Short	0.056	0.056				
		D1	KO	Long	0.167	0.118		-0.577	0.564	
				Short	0.278	0.147				
		D2	KO	Long	0.111	0.073		-1.841	0.066	
				Short	0.500	0.204				
		D3	KO	Long	0.556	0.294		0.000	1.000	
				Short	0.556	0.338				
		D4	KO	Long	1.556	0.475		-1.802	0.072	
				Short	0.444	0.176				
		D5	KO	Long	0.889	0.423		-0.378	0.705	
				Short	0.778	0.278				
		D6	KO	Long	1.222	0.364		-0.312	0.755	
				Short	1.111	0.423				
		D7	KO	Long	0.667	0.333		-1.200	0.230	
				Short	1.333	0.236				
		D8	KO	Long	2.222	0.572		-1.667	0.096	
				Short	1.667	0.707				
		D9	KO	Long	2.778	0.778		-2.388	0.017	*
				Long	0.778	0.278				
		D10	KO	Short	2.222	0.572		-1.282	0.200	
				Long	1.444	0.475				
		D11	KO	Short	2.333	0.471		-1.975	0.048	*
				Long	0.889	0.389				
		D12	KO	Short	1.667	0.408		-0.933	0.351	
				Long	1.222	0.364				
		D13	KO	Short	2.000	0.441		-1.980	0.048	*
				Long	0.778	0.278				
		D14	KO	Short	1.333	0.471		-1.089	0.276	
				Long	0.778	0.222				
		D15	KO	Short	2.000	0.667		-1.581	0.114	
				Long	0.889	0.351				
		D16	KO	Short	1.333	0.441		-1.725	0.084	
				Long	0.556	0.176				
Table S5	Figure 1H,I / Figure S7	D0	WT	Long	0.000	0.000	Wilcoxon Signed Ranks Test	-1.857	0.063	
				Short	0.333	0.144				
		D1	WT	Long	0.222	0.121		-0.378	0.705	
				Short	0.278	0.147				
		D2	WT	Long	0.278	0.188		-0.378	0.705	
				Short	0.222	0.121				
		D3	WT	Long	0.778	0.278		0.000	1.000	
				Short	0.889	0.455				
		D4	WT	Long	0.667	0.289		-1.633	0.102	
				Short	1.444	0.475				
		D5	WT	Long	1.111	0.423		-0.086	0.931	
				Short	1.222	0.278				
		D6	WT	Long	1.000	0.289		-1.179	0.238	
				Short	0.444	0.338				
		D7	WT	Long	2.111	0.696		-1.274	0.203	
				Short	1.333	0.333				
		D8	WT	Long	1.556	0.412		-1.300	0.194	
				Short	0.889	0.261				
		D9	WT	Long	1.889	0.455		-2.157	0.031	*
				Long	0.778	0.278				
		D10	WT	Short	2.111	0.564		-1.590	0.112	
				Long	1.000	0.289				
		D11	WT	Short	1.889	0.484		-2.392	0.017	*
				Long	0.444	0.338				
		D12	WT	Short	1.667	0.471		-0.426	0.670	
				Long	1.333	0.373				
		D13	WT	Short	1.778	0.641		-1.208	0.227	
				Long	0.556	0.294				
		D14	WT	Short	1.889	0.423		-0.137	0.891	
				Long	1.889	0.423				
		D15	WT	Short	1.333	0.408		-0.940	0.347	
				Long	0.778	0.324				
		D16	WT	Short	1.444	0.626		-1.930	0.054	
				Long	0.444	0.242				

5_7

HE frequency in 6–7 s window

Table	Related Figure	Day	Condition	Group	Mean	SEM	Stastistical test	Z	P	*
Table S5	Figure 1H,I / Figure S7	D0	Long	KO	0.333	0.144	Mann-Whitney U Test	-1.403	0.297	
				WT	0.111	0.111				
		D1	Long	KO	0.056	0.056		-0.081	1.000	
				WT	0.111	0.111				
		D2	Long	KO	0.444	0.227		-1.626	0.222	
				WT	0.056	0.056				
		D3	Long	KO	1.111	0.309		-1.973	0.094	
				WT	0.333	0.333				
		D4	Long	KO	0.667	0.289		-0.664	0.546	
				WT	0.889	0.261				
		D5	Long	KO	0.667	0.236		-0.646	0.605	
				WT	0.444	0.176				
		D6	Long	KO	2.111	0.611		-1.414	0.190	
				WT	1.000	0.333				
		D7	Long	KO	0.778	0.324		-1.894	0.077	
				WT	1.667	0.333				
		D8	Long	KO	2.556	0.530		-1.227	0.258	
				WT	2.000	0.289				
		D9	Long	KO	2.111	0.633		-1.796	0.094	
				WT	1.111	0.696				
		D10	Long	KO	1.444	0.338		-1.633	0.136	
				WT	0.667	0.289				
		D11	Long	KO	2.111	0.564		-0.225	0.863	
				WT	2.222	0.778				
		D12	Long	KO	1.556	0.444		-0.727	0.489	
				WT	2.333	0.553				
		D13	Long	KO	1.000	0.373		-1.324	0.222	
				WT	1.889	0.512				
		D14	Long	KO	1.667	0.687		-0.861	0.436	
				WT	2.000	0.500				
		D15	Long	KO	1.444	0.475		-0.691	0.546	
				WT	2.333	0.745				
		D16	Long	KO	1.667	0.500		-0.456	0.666	
				WT	1.333	0.408				
Table S5A	Figure 1H,I / Figure S7	D0	Short	KO	0.444	0.194	Mann-Whitney U Test	-1.514	0.258	
				WT	0.111	0.111				
		D1	Short	KO	0.056	0.056		-1.214	0.387	
				WT	0.333	0.186				
		D2	Short	KO	0.611	0.182		-2.152	0.050	
				WT	0.111	0.073				
		D3	Short	KO	0.889	0.351		-0.287	0.796	
				WT	0.889	0.455				
		D4	Short	KO	0.667	0.289		-0.336	0.796	
				WT	0.778	0.278				
		D5	Short	KO	0.667	0.289		-0.145	0.931	
				WT	0.667	0.236				
		D6	Short	KO	1.444	0.626		-0.139	0.931	
				WT	0.889	0.261				
		D7	Short	KO	0.667	0.289		-0.098	0.931	
				WT	0.889	0.455				
		D8	Short	KO	1.333	0.373		-1.181	0.297	
				WT	0.778	0.324				
		D9	Short	KO	1.778	0.465		-0.732	0.489	
				WT	1.222	0.364				
		D10	Short	KO	0.889	0.351		-0.837	0.436	
				WT	1.222	0.324				
		D11	Short	KO	1.111	0.455		-1.464	0.222	
				WT	0.333	0.236				
		D12	Short	KO	0.778	0.364		-0.202	0.863	
				WT	1.000	0.601				
		D13	Short	KO	0.556	0.242		-0.960	0.387	
				WT	1.000	0.333				
		D14	Short	KO	0.778	0.222		-0.777	0.489	
				WT	0.556	0.242				
		D15	Short	KO	0.444	0.242		-0.607	0.605	
				WT	0.889	0.455				
		D16	Short	KO	0.333	0.167		-0.160	0.931	
				WT	0.444	0.242				

HE frequency in 6–7 s window

Table	Related Figure	Day	Group	Condition	Mean	SEM	Stastistical test	Z	P	*
Table S5	Figure 1H,I / Figure S7	D0	KO	Long	0.333	0.144	Wilcoxon Signed Ranks Test	-0.649	0.516	
				Short	0.444	0.194				
		D1	KO	Long	0.056	0.056		0.000	1.000	
				Short	0.056	0.056				
		D2	KO	Long	0.444	0.227		-0.756	0.450	
				Short	0.611	0.182				
		D3	KO	Long	1.111	0.309		-0.531	0.595	
				Short	0.889	0.351				
		D4	KO	Long	0.667	0.289		-0.108	0.914	
				Short	0.667	0.289				
		D5	KO	Long	0.667	0.236		0.000	1.000	
				Short	0.667	0.289				
		D6	KO	Long	2.111	0.611		-0.850	0.395	
				Short	1.444	0.626				
		D7	KO	Long	0.778	0.324		-0.108	0.914	
				Short	0.667	0.289				
		D8	KO	Long	2.556	0.530		-1.980	0.048	
				Short	1.333	0.373				
		D9	KO	Long	2.111	0.633		-0.345	0.730	
				Long	1.778	0.465				
		D10	KO	Short	1.444	0.338		-1.222	0.222	
				Long	0.889	0.351				
		D11	KO	Short	2.111	0.564		-1.807	0.071	
				Long	1.111	0.455				
		D12	KO	Short	1.556	0.444		-1.282	0.200	
				Long	0.778	0.364				
		D13	KO	Short	1.000	0.373		-0.973	0.330	
				Long	0.556	0.242				
		D14	KO	Short	1.667	0.687		-1.186	0.236	
				Long	0.778	0.222				
		D15	KO	Short	1.444	0.475		-1.807	0.071	
				Long	0.444	0.242				
		D16	KO	Short	1.667	0.500		-2.060	0.039	*
				Long	0.333	0.167				
Table S5	Figure 1H,I / Figure S7	D0	WT	Long	0.111	0.111	Wilcoxon Signed Ranks Test	0.000	1.000	
				Short	0.111	0.111				
		D1	WT	Long	0.111	0.111		-0.921	0.357	
				Short	0.333	0.186				
		D2	WT	Long	0.056	0.056		-0.577	0.564	
				Short	0.111	0.073				
		D3	WT	Long	0.333	0.333		-0.921	0.357	
				Short	0.889	0.455				
		D4	WT	Long	0.889	0.261		-0.447	0.655	
				Short	0.778	0.278				
		D5	WT	Long	0.444	0.176		-0.707	0.480	
				Short	0.667	0.236				
		D6	WT	Long	1.000	0.333		-0.108	0.914	
				Short	0.889	0.261				
		D7	WT	Long	1.667	0.333		-1.329	0.184	
				Short	0.889	0.455				
		D8	WT	Long	2.000	0.289		-2.414	0.016	*
				Short	0.778	0.324				
		D9	WT	Long	1.111	0.696		-0.271	0.786	
				Long	1.222	0.364				
		D10	WT	Short	0.667	0.289		-1.311	0.190	
				Long	1.222	0.324				
		D11	WT	Short	2.222	0.778		-2.214	0.027	*
				Long	0.333	0.236				
		D12	WT	Short	2.333	0.553		-1.257	0.209	
				Long	1.000	0.601				
		D13	WT	Short	1.889	0.512		-1.098	0.272	
				Long	1.000	0.333				
		D14	WT	Short	2.000	0.500		-2.136	0.033	*
				Long	0.556	0.242				
		D15	WT	Short	2.333	0.745		-1.715	0.086	
				Long	0.889	0.455				
		D16	WT	Short	1.333	0.408		-1.552	0.121	
				Long	0.444	0.242				

5_8

HE frequency in 7–8 s window

Table	Related Figure	Day	Condition	Group	Mean	SEM	Stastistical test	Z	P	*
Table S5	Figure 1H,I / Figure S7	D0	Long	KO	0.167	0.167	Mann-Whitney U Test	0.000	1.000	
				WT	0.222	0.222				
		D1	Long	KO	0.222	0.147		-1.000	0.730	
				WT	0.000	0.000				
		D2	Long	KO	0.389	0.232		-1.837	0.258	
				WT	0.000	0.000				
		D3	Long	KO	0.222	0.147		-1.055	0.387	
				WT	0.333	0.167				
		D4	Long	KO	0.889	0.200		-0.520	0.666	
				WT	0.667	0.236				
		D5	Long	KO	0.889	0.423		-0.646	0.605	
				WT	0.444	0.176				
		D6	Long	KO	1.000	0.333		-0.972	0.436	
				WT	0.444	0.176				
		D7	Long	KO	1.444	0.603		-0.627	0.605	
				WT	0.889	0.351				
		D8	Long	KO	2.222	0.662		-0.745	0.489	
				WT	1.778	0.641				
		D9	Long	KO	1.667	0.373		-0.138	0.931	
				WT	1.333	0.471				
		D10	Long	KO	1.778	0.662		-0.738	0.489	
				WT	1.556	0.556				
		D11	Long	KO	0.889	0.423		-0.320	0.796	
				WT	1.778	0.683				
		D12	Long	KO	1.889	0.564		-1.226	0.258	
				WT	1.444	0.444				
		D13	Long	KO	1.667	0.500		-0.749	0.489	
				WT	1.333	0.471				
		D14	Long	KO	1.889	0.512		-2.237	0.031	*
				WT	2.333	0.373				
		D15	Long	KO	0.889	0.389		-0.949	0.387	
				WT	2.000	0.408				
		D16	Long	KO	1.667	0.471		-1.660	0.113	
				WT	2.111	0.633				
Table S5A	Figure 1H,I / Figure S7	D0	Short	KO	0.333	0.118	Mann-Whitney U Test	-1.642	0.190	
				WT	0.222	0.222				
		D1	Short	KO	0.389	0.200		-0.620	0.666	
				WT	0.056	0.056				
		D2	Short	KO	0.611	0.232		-0.879	0.436	
				WT	0.222	0.121				
		D3	Short	KO	0.444	0.242		-0.620	0.666	
				WT	0.778	0.324				
		D4	Short	KO	0.889	0.200		0.000	1.000	
				WT	1.000	0.373				
		D5	Short	KO	0.889	0.261		-0.525	0.666	
				WT	0.667	0.236				
		D6	Short	KO	0.778	0.278		-0.474	0.666	
				WT	0.222	0.147				
		D7	Short	KO	1.333	0.527		-0.092	0.931	
				WT	0.556	0.242				
		D8	Short	KO	1.222	0.641		-1.321	0.222	
				WT	1.333	0.687				
		D9	Short	KO	1.000	0.667		-1.684	0.113	
				WT	1.667	0.799				
		D10	Short	KO	0.556	0.294		-1.639	0.136	
				WT	0.889	0.261				
		D11	Short	KO	1.000	0.333		-0.567	0.605	
				WT	1.444	0.626				
		D12	Short	KO	1.333	0.577		-0.869	0.436	
				WT	0.778	0.324				
		D13	Short	KO	1.000	0.553		-1.388	0.190	
				WT	1.000	0.500				
		D14	Short	KO	1.111	0.455		-1.139	0.297	
				WT	1.111	0.423				
		D15	Short	KO	1.000	0.333		-0.422	0.730	
				WT	1.444	0.503				
		D16	Short	KO	0.889	0.389		-1.286	0.222	
				WT	0.889	0.455				

HE frequency in 7–8 s window

Table	Related Figure	Day	Group	Condition	Mean	SEM	Stastistical test	Z	P	*
Table S5	Figure 1H,I / Figure S7	D0	KO	Long	0.167	0.167	Wilcoxon Signed Ranks Test	-1.342	0.180	
				Short	0.333	0.118				
		D1	KO	Long	0.222	0.147		-0.756	0.450	
				Short	0.389	0.200				
		D2	KO	Long	0.389	0.232		-0.816	0.414	
				Short	0.611	0.232				
		D3	KO	Long	0.222	0.147		-1.414	0.157	
				Short	0.444	0.242				
		D4	KO	Long	0.889	0.200		0.000	1.000	
				Short	0.889	0.200				
		D5	KO	Long	0.889	0.423		0.000	1.000	
				Short	0.889	0.261				
		D6	KO	Long	1.000	0.333		-0.816	0.414	
				Short	0.778	0.278				
		D7	KO	Long	1.444	0.603		-0.333	0.739	
				Short	1.333	0.527				
		D8	KO	Long	2.222	0.662		-1.807	0.071	
				Short	1.222	0.641				
		D9	KO	Long	1.667	0.373		-0.828	0.408	
				Long	1.000	0.667				
		D10	KO	Short	1.778	0.662		-2.060	0.039	*
				Long	0.556	0.294				
		D11	KO	Short	0.889	0.423		-0.276	0.783	
				Long	1.000	0.333				
		D12	KO	Short	1.889	0.564		-1.289	0.197	
				Long	1.333	0.577				
		D13	KO	Short	1.667	0.500		-2.121	0.034	*
				Long	1.000	0.553				
		D14	KO	Short	1.889	0.512		-1.200	0.230	
				Long	1.111	0.455				
		D15	KO	Short	0.889	0.389		-0.577	0.564	
				Long	1.000	0.333				
		D16	KO	Short	1.667	0.471		-1.890	0.059	
				Long	0.889	0.389				
Table S5	Figure 1H,I / Figure S7	D0	WT	Long	0.222	0.222	Wilcoxon Signed Ranks Test	0.000	1.000	
				Short	0.222	0.222				
		D1	WT	Long	0.000	0.000		-1.000	0.317	
				Short	0.056	0.056				
		D2	WT	Long	0.000	0.000		-1.633	0.102	
				Short	0.222	0.121				
		D3	WT	Long	0.333	0.167		-1.633	0.102	
				Short	0.778	0.324				
		D4	WT	Long	0.667	0.236		-0.680	0.496	
				Short	1.000	0.373				
		D5	WT	Long	0.444	0.176		-0.707	0.480	
				Short	0.667	0.236				
		D6	WT	Long	0.444	0.176		-1.000	0.317	
				Short	0.222	0.147				
		D7	WT	Long	0.889	0.351		-0.750	0.453	
				Short	0.556	0.242				
		D8	WT	Long	1.778	0.641		-0.850	0.395	
				Short	1.333	0.687				
		D9	WT	Long	1.333	0.471		-0.378	0.705	
				Long	1.667	0.799				
		D10	WT	Short	1.556	0.556		-1.604	0.109	
				Long	0.889	0.261				
		D11	WT	Short	1.778	0.683		-0.816	0.414	
				Long	1.444	0.626				
		D12	WT	Short	1.444	0.444		-1.604	0.109	
				Long	0.778	0.324				
		D13	WT	Short	1.333	0.471		-1.134	0.257	
				Long	1.000	0.500				
		D14	WT	Short	2.333	0.373		-2.414	0.016	*
				Long	1.111	0.423				
		D15	WT	Short	2.000	0.408		-1.414	0.157	
				Long	1.444	0.503				
		D16	WT	Short	2.111	0.633		-1.807	0.071	
				Long	0.889	0.455				

5_9

HE frequency in 8–9 s window

Table	Related Figure	Day	Condition	Group	Mean	SEM	Stastistical test	Z	P	*
Table S5	Figure 1H,I / Figure S7	D0	Long	KO	0.000	0.000	Mann-Whitney U Test	-1.000	0.730	
				WT	0.111	0.111				
		D1	Long	KO	0.111	0.111		-1.000	0.730	
				WT	0.000	0.000				
		D2	Long	KO	0.167	0.118		-0.122	0.931	
				WT	0.222	0.147				
		D3	Long	KO	0.333	0.236		-1.455	0.436	
				WT	0.000	0.000				
		D4	Long	KO	0.667	0.236		-0.797	0.489	
				WT	1.222	0.434				
		D5	Long	KO	1.111	0.588		-0.846	0.436	
				WT	1.111	0.309				
		D6	Long	KO	1.889	0.539		-1.799	0.094	
				WT	0.667	0.333				
		D7	Long	KO	1.333	0.333		-1.101	0.340	
				WT	0.889	0.351				
		D8	Long	KO	2.111	0.423		-2.226	0.031	*
				WT	0.778	0.278				
		D9	Long	KO	2.444	0.556		-1.492	0.161	
				WT	1.222	0.434				
		D10	Long	KO	1.889	0.564		-0.187	0.863	
				WT	2.000	0.601				
		D11	Long	KO	1.778	0.954		-0.321	0.796	
				WT	1.444	0.475				
		D12	Long	KO	1.444	0.556		0.000	1.000	
				WT	1.444	0.556				
		D13	Long	KO	2.111	0.655		-0.677	0.546	
				WT	1.444	0.475				
		D14	Long	KO	1.889	0.512		-0.284	0.796	
				WT	1.778	0.521				
		D15	Long	KO	1.222	0.596		-0.736	0.489	
				WT	1.333	0.333				
		D16	Long	KO	1.111	0.455		-1.059	0.340	
				WT	2.000	0.707				
Table S5A	Figure 1H,I / Figure S7	D0	Short	KO	0.222	0.121	Mann-Whitney U Test	-0.620	0.666	
				WT	0.111	0.073				
		D1	Short	KO	0.167	0.118		-0.561	0.666	
				WT	0.278	0.147				
		D2	Short	KO	0.556	0.227		-1.464	0.222	
				WT	0.167	0.118				
		D3	Short	KO	0.222	0.147		-1.910	0.094	
				WT	0.778	0.222				
		D4	Short	KO	0.667	0.333		-0.903	0.436	
				WT	1.333	0.500				
		D5	Short	KO	0.444	0.176		-0.830	0.489	
				WT	0.778	0.278				
		D6	Short	KO	1.222	0.494		-0.472	0.666	
				WT	0.778	0.324				
		D7	Short	KO	0.333	0.236		-1.809	0.113	
				WT	1.111	0.351				
		D8	Short	KO	0.778	0.324		-1.937	0.063	
				WT	1.778	0.401				
		D9	Short	KO	1.778	0.521		-1.943	0.063	
				WT	0.556	0.242				
		D10	Short	KO	1.111	0.351		-0.186	0.863	
				WT	1.111	0.423				
		D11	Short	KO	0.778	0.324		-1.133	0.297	
				WT	1.222	0.324				
		D12	Short	KO	0.444	0.176		0.000	1.000	
				WT	0.444	0.176				
		D13	Short	KO	0.556	0.242		-0.199	0.863	
				WT	0.556	0.176				
		D14	Short	KO	0.667	0.236		-0.096	0.931	
				WT	0.778	0.364				
		D15	Short	KO	0.111	0.111		-1.102	0.436	
				WT	0.333	0.167				
		D16	Short	KO	0.333	0.236		-1.132	0.340	
				WT	0.556	0.176				

HE frequency in 8–9 s window

Table	Related Figure	Day	Group	Condition	Mean	SEM	Stastistical test	Z	P	*
Table S5	Figure 1H,I / Figure S7	D0	KO	Long	0.000	0.000	Wilcoxon Signed Ranks Test	-1.633	0.102	
				Short	0.222	0.121				
		D1	KO	Long	0.111	0.111		-0.447	0.655	
				Short	0.167	0.118				
		D2	KO	Long	0.167	0.118		-1.511	0.131	
				Short	0.556	0.227				
		D3	KO	Long	0.333	0.236		-0.447	0.655	
				Short	0.222	0.147				
		D4	KO	Long	0.667	0.236		0.000	1.000	
				Short	0.667	0.333				
		D5	KO	Long	1.111	0.588		-0.647	0.518	
				Short	0.444	0.176				
		D6	KO	Long	1.889	0.539		-1.730	0.084	
				Short	1.222	0.494				
		D7	KO	Long	1.333	0.333		-2.309	0.021	*
				Short	0.333	0.236				
		D8	KO	Long	2.111	0.423		-2.111	0.035	*
				Short	0.778	0.324				
		D9	KO	Long	2.444	0.556		-1.561	0.119	
				Long	1.778	0.521				
		D10	KO	Short	1.889	0.564		-0.781	0.435	
				Long	1.111	0.351				
		D11	KO	Short	1.778	0.954		-1.511	0.131	
				Long	0.778	0.324				
		D12	KO	Short	1.444	0.556		-1.807	0.071	
				Long	0.444	0.176				
		D13	KO	Short	2.111	0.655		-2.047	0.041	*
				Long	0.556	0.242				
		D14	KO	Short	1.889	0.512		-2.456	0.014	*
				Long	0.667	0.236				
		D15	KO	Short	1.222	0.596		-1.604	0.109	
				Long	0.111	0.111				
		D16	KO	Short	1.111	0.455		-1.841	0.066	
				Long	0.333	0.236				
Table S5	Figure 1H,I / Figure S7	D0	WT	Long	0.111	0.111	Wilcoxon Signed Ranks Test	0.000	1.000	
				Short	0.111	0.073				
		D1	WT	Long	0.000	0.000		-1.633	0.102	
				Short	0.278	0.147				
		D2	WT	Long	0.222	0.147		-0.447	0.655	
				Short	0.167	0.118				
		D3	WT	Long	0.000	0.000		-2.333	0.020	*
				Short	0.778	0.222				
		D4	WT	Long	1.222	0.434		-0.333	0.739	
				Short	1.333	0.500				
		D5	WT	Long	1.111	0.309		-0.707	0.480	
				Short	0.778	0.278				
		D6	WT	Long	0.667	0.333		-0.276	0.783	
				Short	0.778	0.324				
		D7	WT	Long	0.889	0.351		-0.513	0.608	
				Short	1.111	0.351				
		D8	WT	Long	0.778	0.278		-1.913	0.056	
				Short	1.778	0.401				
		D9	WT	Long	1.222	0.434		-0.997	0.319	
				Long	0.556	0.242				
		D10	WT	Short	2.000	0.601		-1.613	0.107	
				Long	1.111	0.423				
		D11	WT	Short	1.444	0.475		-0.707	0.480	
				Long	1.222	0.324				
		D12	WT	Short	1.444	0.556		-1.807	0.071	
				Long	0.444	0.176				
		D13	WT	Short	1.444	0.475		-1.492	0.136	
				Long	0.556	0.176				
		D14	WT	Short	1.778	0.521		-1.567	0.117	
				Long	0.778	0.364				
		D15	WT	Short	1.333	0.333		-2.251	0.024	*
				Long	0.333	0.167				
		D16	WT	Short	2.000	0.707		-1.807	0.071	
				Long	0.556	0.176				

5_10

HE frequency in 9–10 s window

Table	Related Figure	Day	Condition	Group	Mean	SEM	Statistical test	Z	P	*
Table S5	Figure 1H,I / Figure S7	D0	Long	KO	0.222	0.147	Mann-Whitney U Test	-1.458	0.436	
				WT	0.000	0.000				
		D1	Long	KO	0.056	0.056		-1.636	0.19	
				WT	0.000	0.000				
		D2	Long	KO	0.222	0.222		-0.910	0.546	
				WT	0.444	0.155				
		D3	Long	KO	0.222	0.147		-0.586	0.605	
				WT	0.556	0.242				
		D4	Long	KO	0.444	0.242		-1.628	0.136	
				WT	0.333	0.333				
		D5	Long	KO	0.556	0.242		-0.279	0.796	
				WT	0.667	0.236				
		D6	Long	KO	1.000	0.289		-0.185	0.863	
				WT	1.222	0.324				
		D7	Long	KO	0.889	0.309		-0.482	0.666	
				WT	1.111	0.351				
		D8	Long	KO	2.333	0.707		-0.049	1	
				WT	1.000	0.667				
		D9	Long	KO	1.444	0.444		-1.000	0.73	
				WT	1.778	0.760				
		D10	Long	KO	1.222	0.572		-1.068	0.387	
				WT	1.444	0.530				
		D11	Long	KO	1.000	0.441		-0.391	0.73	
				WT	1.444	0.626				
		D12	Long	KO	1.444	0.626		-0.419	0.73	
				WT	1.778	0.778				
		D13	Long	KO	0.556	0.294		-0.137	0.931	
				WT	1.222	0.434				
		D14	Long	KO	0.889	0.539		-0.327	0.796	
				WT	1.000	0.441				
		D15	Long	KO	1.444	0.475		-1.241	0.258	
				WT	1.667	0.667				
		D16	Long	KO	1.000	0.500		-0.046	1	
				WT	1.111	0.588				
Table S5A	Figure 1H,I / Figure S7	D0	Short	KO	0.278	0.147	Mann-Whitney U Test	-0.168	0.931	
				WT	0.444	0.306				
		D1	Short	KO	0.778	0.334		-0.910	0.436	
				WT	0.056	0.056				
		D2	Short	KO	0.167	0.083		-0.819	0.489	
				WT	0.556	0.256				
		D3	Short	KO	0.556	0.294		-0.691	0.546	
				WT	0.222	0.147				
		D4	Short	KO	0.778	0.465		-1.866	0.094	
				WT	0.778	0.222				
		D5	Short	KO	0.778	0.278		-1.338	0.222	
				WT	1.000	0.289				
		D6	Short	KO	1.333	0.373		-1.735	0.113	
				WT	1.000	0.373				
		D7	Short	KO	0.667	0.167		-0.607	0.605	
				WT	1.000	0.333				
		D8	Short	KO	1.333	0.441		-0.615	0.73	
				WT	0.333	0.167				
		D9	Short	KO	0.889	0.423		-2.107	0.077	
				WT	0.889	0.309				
		D10	Short	KO	0.556	0.338		-0.729	0.605	
				WT	1.111	0.351				
		D11	Short	KO	1.000	0.441		-0.563	0.605	
				WT	0.778	0.324				
		D12	Short	KO	1.111	0.351		-0.595	0.605	
				WT	0.333	0.167				
		D13	Short	KO	1.000	0.441		-0.478	0.666	
				WT	1.000	0.289				
		D14	Short	KO	0.444	0.242		-0.239	0.863	
				WT	0.889	0.455				
		D15	Short	KO	0.556	0.176		-0.420	0.73	
				WT	0.222	0.222				
		D16	Short	KO	0.222	0.147		-1.655	0.19	
				WT	0.111	0.111				

HE frequency in 9–10 s window

Table	Related Figure	Day	Group	Condition	Mean	SEM	Statistical test	Z	P	*
Table S5	Figure 1H,I / Figure S7	D0	KO	Long	0.222	0.147	Wilcoxon Signed Ranks Test	-0.141	0.888	
				Short	0.278	0.147				
		D1	KO	Long	0.056	0.056		0	1.000	
				Short	0.778	0.334				
		D2	KO	Long	0.222	0.222		-0.378	0.705	
				Short	0.167	0.083				
		D3	KO	Long	0.222	0.147		-0.722	0.470	
				Short	0.556	0.294				
		D4	KO	Long	0.444	0.242		-1.604	0.109	
				Short	0.778	0.465				
		D5	KO	Long	0.556	0.242		-1.166	0.244	
				Short	0.778	0.278				
		D6	KO	Long	1.000	0.289		-0.535	0.593	
				Short	1.333	0.373				
		D7	KO	Long	0.889	0.309		-1.134	0.257	
				Short	0.667	0.167				
		D8	KO	Long	2.333	0.707		-1.841	0.066	
				Short	1.333	0.441				
		D9	KO	Long	1.444	0.444		-1.841	0.066	
				Short	0.889	0.423				
		D10	KO	Short	1.222	0.572		-1.134	0.257	
				Long	0.556	0.338				
		D11	KO	Short	1.000	0.441		-0.649	0.516	
				Long	1.000	0.441				
		D12	KO	Short	1.444	0.626		-0.816	0.414	
				Long	1.111	0.351				
		D13	KO	Short	0.556	0.294		-0.957	0.339	
				Long	1.000	0.441				
		D14	KO	Short	0.889	0.539		0	1.000	
				Long	0.444	0.242				
		D15	KO	Short	1.444	0.475		-0.85	0.395	
				Long	0.556	0.176				
		D16	KO	Short	1.000	0.500		-1.947	0.052	
				Long	0.222	0.147				
Table S5	Figure 1H,I / Figure S7	D0	WT	Long	0.000	0.000	Wilcoxon Signed Ranks Test	-1.342	0.180	
				Short	0.444	0.306				
		D1	WT	Long	0.000	0.000		-0.345	0.730	
				Short	0.056	0.056				
		D2	WT	Long	0.444	0.155		-1.228	0.219	
				Short	0.556	0.256				
		D3	WT	Long	0.556	0.242		-0.425	0.671	
				Short	0.222	0.147				
		D4	WT	Long	0.333	0.333		-0.647	0.518	
				Short	0.778	0.222				
		D5	WT	Long	0.667	0.236		-0.638	0.524	
				Short	1.000	0.289				
		D6	WT	Long	1.222	0.324		-1.807	0.071	
				Short	1.000	0.373				
		D7	WT	Long	1.111	0.351		-0.264	0.792	
				Short	1.000	0.333				
		D8	WT	Long	1.000	0.667		-1.841	0.066	
				Short	0.333	0.167				
		D9	WT	Long	1.778	0.760		-1	0.317	
				Short	0.889	0.309				
		D10	WT	Short	1.444	0.530		-1.732	0.083	
				Long	1.111	0.351				
		D11	WT	Short	1.444	0.626		-0.905	0.366	
				Long	0.778	0.324				
		D12	WT	Short	1.778	0.778		-0.172	0.863	
				Long	0.333	0.167				
		D13	WT	Short	1.222	0.434		-0.954	0.340	
				Long	1.000	0.289				
		D14	WT	Short	1.000	0.441		-0.711	0.477	
				Long	0.889	0.455				
		D15	WT	Short	1.667	0.667		-0.412	0.680	
				Long	0.222	0.222				
		D16	WT	Short	1.111	0.588		-1.807	0.071	
				Long	0.111	0.111				

5_11

HE frequency in 10–11 s window

Table	Related Figure	Day	Condition	Group	Mean	SEM	Statistical test	Z	P	*
Table S5	Figure 1H,I / Figure S7	D0	Long	KO	0.111	0.073	Mann-Whitney U Test	-0.243	0.863	
				WT	0.556	0.444				
		D1	Long	KO	0.111	0.111		-1.000	0.730	
				WT	0.000	0.000				
		D2	Long	KO	0.222	0.121		-1.837	0.258	
				WT	0.222	0.147				
		D3	Long	KO	0.222	0.222		-1.092	0.436	
				WT	0.667	0.373				
		D4	Long	KO	1.000	0.471		-0.605	0.605	
				WT	0.667	0.441				
		D5	Long	KO	1.111	0.389		-1.196	0.297	
				WT	0.444	0.176				
		D6	Long	KO	2.000	0.726		-1.862	0.113	
				WT	0.222	0.147				
		D7	Long	KO	1.333	0.527		-0.558	0.605	
				WT	1.000	0.441				
		D8	Long	KO	1.667	0.408		-1.278	0.222	
				WT	1.000	0.441				
		D9	Long	KO	1.667	0.707		-0.413	0.730	
				WT	2.111	0.904				
		D10	Long	KO	1.000	0.471		-0.792	0.489	
				WT	1.444	0.475				
		D11	Long	KO	0.778	0.364		-1.392	0.190	
				WT	2.333	0.833				
		D12	Long	KO	0.778	0.364		-1.249	0.258	
				WT	1.333	0.333				
		D13	Long	KO	1.556	0.603		-0.230	0.863	
				WT	1.556	0.530				
		D14	Long	KO	0.778	0.434		-0.733	0.546	
				WT	1.111	0.423				
		D15	Long	KO	0.444	0.242		-1.006	0.387	
				WT	1.667	0.707				
		D16	Long	KO	1.333	0.471		-0.279	0.796	
				WT	1.889	0.772				
Table S5A	Figure 1H,I / Figure S7	D0	Short	KO	0.278	0.121	Mann-Whitney U Test	-1.068	0.387	
				WT	0.111	0.073				
		D1	Short	KO	0.389	0.200		-0.620	0.666	
				WT	0.222	0.147				
		D2	Short	KO	0.778	0.355		-1.896	0.113	
				WT	0.111	0.111				
		D3	Short	KO	0.778	0.364		-0.454	0.730	
				WT	0.556	0.294				
		D4	Short	KO	0.444	0.176		-0.646	0.605	
				WT	0.667	0.236				
		D5	Short	KO	0.333	0.236		-0.952	0.436	
				WT	0.667	0.289				
		D6	Short	KO	1.000	0.333		-0.756	0.489	
				WT	0.667	0.289				
		D7	Short	KO	1.111	0.423		-0.098	0.931	
				WT	1.111	0.389				
		D8	Short	KO	0.556	0.176		-0.049	1.000	
				WT	1.000	0.500				
		D9	Short	KO	0.778	0.364		-0.147	0.931	
				WT	0.667	0.333				
		D10	Short	KO	1.000	0.289		-1.196	0.297	
				WT	0.778	0.434				
		D11	Short	KO	0.778	0.324		-0.657	0.546	
				WT	1.000	0.236				
		D12	Short	KO	1.000	0.289		-1.738	0.113	
				WT	0.333	0.167				
		D13	Short	KO	0.333	0.236		-0.680	0.666	
				WT	0.111	0.111				
		D14	Short	KO	0.667	0.236		-0.096	0.931	
				WT	0.778	0.364				
		D15	Short	KO	0.333	0.167		-0.160	0.931	
				WT	0.444	0.242				
		D16	Short	KO	0.222	0.147		-1.982	0.077	
				WT	1.000	0.333				

HE frequency in 10–11 s window

Table	Related Figure	Day	Group	Condition	Mean	SEM	Statistical test	Z	P	*
Table S5	Figure 1H,I / Figure S7	D0	KO	Long	0.111	0.073	Wilcoxon Signed Ranks Test	-1.000	0.317	
				Short	0.278	0.121				
		D1	KO	Long	0.111	0.111		-1.633	0.102	
				Short	0.389	0.200				
		D2	KO	Long	0.222	0.121		-1.355	0.176	
				Short	0.778	0.355				
		D3	KO	Long	0.222	0.222		-1.089	0.276	
				Short	0.778	0.364				
		D4	KO	Long	1.000	0.471		-1.225	0.221	
				Short	0.444	0.176				
		D5	KO	Long	1.111	0.389		-1.594	0.111	
				Short	0.333	0.236				
		D6	KO	Long	2.000	0.726		-1.518	0.129	
				Short	1.000	0.333				
		D7	KO	Long	1.333	0.527		-0.816	0.414	
				Short	1.111	0.423				
		D8	KO	Long	1.667	0.408		-1.983	0.047	*
				Short	0.556	0.176				
		D9	KO	Long	1.667	0.707		-1.160	0.246	
				Short	0.778	0.364				
		D10	KO	Short	1.000	0.471		0.000	1.000	
				Long	1.000	0.289				
		D11	KO	Short	0.778	0.364		0.000	1.000	
				Long	0.778	0.324				
		D12	KO	Short	0.778	0.364		-0.649	0.516	
				Long	1.000	0.289				
		D13	KO	Short	1.556	0.603		-2.232	0.026	*
				Long	0.333	0.236				
		D14	KO	Short	0.778	0.434		-0.378	0.705	
				Long	0.667	0.236				
		D15	KO	Short	0.444	0.242		-0.577	0.564	
				Long	0.333	0.167				
		D16	KO	Short	1.333	0.471		-1.715	0.086	
				Long	0.222	0.147				
Table S5	Figure 1H,I / Figure S7	D0	WT	Long	0.556	0.444	Wilcoxon Signed Ranks Test	-0.736	0.461	
				Short	0.111	0.073				
		D1	WT	Long	0.000	0.000		-1.414	0.157	
				Short	0.222	0.147				
		D2	WT	Long	0.000	0.000		-1.000	0.317	
				Short	0.111	0.111				
		D3	WT	Long	0.667	0.373		-0.184	0.854	
				Short	0.556	0.294				
		D4	WT	Long	0.667	0.441		-0.378	0.705	
				Short	0.667	0.236				
		D5	WT	Long	0.444	0.176		-0.707	0.480	
				Short	0.667	0.289				
		D6	WT	Long	0.222	0.147		-1.300	0.194	
				Short	0.667	0.289				
		D7	WT	Long	1.000	0.441		-0.447	0.655	
				Short	1.111	0.389				
		D8	WT	Long	1.000	0.441		-0.333	0.739	
				Short	1.000	0.500				
		D9	WT	Long	2.111	0.904		-1.913	0.056	
				Short	0.667	0.333				
		D10	WT	Short	1.444	0.475		-1.089	0.276	
				Long	0.778	0.434				
		D11	WT	Short	2.333	0.833		-1.294	0.196	
				Long	1.000	0.236				
		D12	WT	Short	1.333	0.333		-2.251	0.024	*
				Long	0.333	0.167				
		D13	WT	Short	1.556	0.530		-2.226	0.026	*
				Long	0.111	0.111				
		D14	WT	Short	1.111	0.423		-1.342	0.180	
				Long	0.778	0.364				
		D15	WT	Short	1.667	0.707		-1.581	0.114	
				Long	0.444	0.242				
		D16	WT	Short	1.889	0.772		-0.679	0.497	
				Long	1.000	0.333				

5_12

HE frequency in 11–12 s window

Table	Related Figure	Day	Condition	Group	Mean	SEM	Statistical test	Z	P	*
Table S5	Figure 1H,I / Figure S7	D0	Long	KO	0.278	0.188	Mann-Whitney U Test	-0.476	0.796	
				WT	0.444	0.444				
		D1	Long	KO	0.111	0.073		-0.122	0.931	
				WT	0.167	0.118				
		D2	Long	KO	0.222	0.147		-0.281	0.863	
				WT	0.222	0.121				
		D3	Long	KO	0.222	0.147		0.000	1.000	
				WT	0.222	0.147				
		D4	Long	KO	0.778	0.434		-0.098	0.931	
				WT	0.667	0.289				
		D5	Long	KO	1.556	0.475		-1.483	0.190	
				WT	0.667	0.373				
		D6	Long	KO	0.667	0.441		-0.053	1.000	
				WT	0.444	0.242				
		D7	Long	KO	1.111	0.351		-0.279	0.796	
				WT	1.222	0.547				
		D8	Long	KO	2.111	0.633		-1.231	0.258	
				WT	1.111	0.423				
		D9	Long	KO	2.333	0.833		-0.225	0.863	
				WT	1.778	0.547				
		D10	Long	KO	1.000	0.500		-0.476	0.666	
				WT	1.333	0.471				
		D11	Long	KO	0.778	0.364		-0.098	0.931	
				WT	0.889	0.423				
		D12	Long	KO	1.000	0.667		-0.252	0.863	
				WT	0.667	0.289				
		D13	Long	KO	0.556	0.294		-0.355	0.796	
				WT	0.667	0.289				
		D14	Long	KO	0.889	0.455		-0.713	0.546	
				WT	1.111	0.351				
		D15	Long	KO	1.222	0.547		-0.047	1.000	
				WT	1.000	0.373				
		D16	Long	KO	1.667	0.726		-1.211	0.340	
				WT	0.444	0.338				
Table S5A	Figure 1H,I / Figure S7	D0	Short	KO	0.389	0.232	Mann-Whitney U Test	-1.835	0.258	
				WT	0.000	0.000				
		D1	Short	KO	0.167	0.118		-0.476	0.796	
				WT	0.222	0.222				
		D2	Short	KO	0.222	0.147		-0.122	0.931	
				WT	0.167	0.118				
		D3	Short	KO	0.333	0.167		-0.318	0.796	
				WT	0.556	0.294				
		D4	Short	KO	0.333	0.236		-1.568	0.190	
				WT	1.444	0.648				
		D5	Short	KO	0.889	0.200		-0.382	0.730	
				WT	1.222	0.401				
		D6	Short	KO	1.333	0.333		-3.181	0.004	**
				WT	0.000	0.000				
		D7	Short	KO	0.889	0.351		-0.684	0.546	
				WT	0.667	0.373				
		D8	Short	KO	0.889	0.309		-0.927	0.387	
				WT	1.333	0.333				
		D9	Short	KO	1.000	0.553		-0.287	0.796	
				WT	0.889	0.351				
		D10	Short	KO	0.444	0.242		-1.350	0.222	
				WT	1.000	0.333				
		D11	Short	KO	1.222	0.434		-0.465	0.666	
				WT	1.111	0.539				
		D12	Short	KO	0.667	0.333		-0.199	0.863	
				WT	0.556	0.176				
		D13	Short	KO	0.444	0.176		-0.397	0.730	
				WT	0.667	0.289				
		D14	Short	KO	0.222	0.147		0.000	1.000	
				WT	0.222	0.147				
		D15	Short	KO	0.222	0.147		-0.511	0.730	
				WT	0.333	0.167				
		D16	Short	KO	0.222	0.147		0.000	1.000	
				WT	0.222	0.147				

HE frequency in 11–12 s window

Table	Related Figure	Day	Group	Condition	Mean	SEM	Statistical test	Z	P	*
Table S5	Figure 1H,I / Figure S7	D0	KO	Long	0.278	0.188	Wilcoxon Signed Ranks Test	-0.271	0.786	
				Short	0.389	0.232				
		D1	KO	Long	0.111	0.073		-0.447	0.655	
				Short	0.167	0.118				
		D2	KO	Long	0.222	0.147		0.000	1.000	
				Short	0.222	0.147				
		D3	KO	Long	0.222	0.147		-0.577	0.564	
				Short	0.333	0.167				
		D4	KO	Long	0.778	0.434		-1.633	0.102	
				Short	0.333	0.236				
		D5	KO	Long	1.556	0.475		-1.294	0.196	
				Short	0.889	0.200				
		D6	KO	Long	0.667	0.441		-1.387	0.165	
				Short	1.333	0.333				
		D7	KO	Long	1.111	0.351		-0.816	0.414	
				Short	0.889	0.351				
		D8	KO	Long	2.111	0.633		-1.420	0.156	
				Short	0.889	0.309				
		D9	KO	Long	2.333	0.833		-1.841	0.066	
				Short	1.000	0.553				
		D10	KO	Short	1.000	0.500		-0.736	0.461	
				Long	0.444	0.242				
		D11	KO	Short	0.778	0.364		-1.265	0.206	
				Long	1.222	0.434				
		D12	KO	Short	1.000	0.667		-0.318	0.750	
				Long	0.667	0.333				
		D13	KO	Short	0.556	0.294		-0.333	0.739	
				Long	0.444	0.176				
		D14	KO	Short	0.889	0.455		-1.604	0.109	
				Long	0.222	0.147				
		D15	KO	Short	1.222	0.547		-1.382	0.167	
				Long	0.222	0.147				
		D16	KO	Short	1.667	0.726		-1.753	0.080	
				Long	0.222	0.147				
Table S5	Figure 1H,I / Figure S7	D0	WT	Long	0.444	0.444	Wilcoxon Signed Ranks Test	-1.000	0.317	
				Short	0.000	0.000				
		D1	WT	Long	0.167	0.118		0.000	1.000	
				Short	0.222	0.222				
		D2	WT	Long	0.222	0.121		-1.000	0.317	
				Short	0.167	0.118				
		D3	WT	Long	0.222	0.147		-1.089	0.276	
				Short	0.556	0.294				
		D4	WT	Long	0.667	0.289		-1.473	0.141	
				Short	1.444	0.648				
		D5	WT	Long	0.667	0.373		-0.940	0.347	
				Short	1.222	0.401				
		D6	WT	Long	0.444	0.242		-1.633	0.102	
				Short	0.000	0.000				
		D7	WT	Long	1.222	0.547		-0.962	0.336	
				Short	0.667	0.373				
		D8	WT	Long	1.111	0.423		-0.520	0.603	
				Short	1.333	0.333				
		D9	WT	Long	1.778	0.547		-1.511	0.131	
				Short	0.889	0.351				
		D10	WT	Short	1.333	0.471		-0.750	0.453	
				Long	1.000	0.333				
		D11	WT	Short	0.889	0.423		-0.707	0.480	
				Long	1.111	0.539				
		D12	WT	Short	0.667	0.289		-0.333	0.739	
				Long	0.556	0.176				
		D13	WT	Short	0.667	0.289		0.000	1.000	
				Long	0.667	0.289				
		D14	WT	Short	1.111	0.351		-2.070	0.038	*
				Long	0.222	0.147				
		D15	WT	Short	1.000	0.373		-1.656	0.098	
				Long	0.333	0.167				
		D16	WT	Short	0.444	0.338		-0.378	0.705	
				Long	0.222	0.147				

5_13

HE frequency in 12–13 s window										
Table	Related Figure	Day	Condition	Group	Mean	SEM	Stastistical test	Z	P	*
Table S5	Figure 1H,I / Figure S7	D0	Long	KO	0.278	0.147	Mann-Whitney U Test	-1.035	0.489	
				WT	0.111	0.111				
		D1	Long	KO	0.056	0.056		-0.615	0.730	
				WT	0.111	0.073				
		D2	Long	KO	0.333	0.236		-1.455	0.436	
				WT	0.000	0.000				
		D3	Long	KO	0.556	0.338		-1.253	0.258	
				WT	1.000	0.333				
		D4	Long	KO	0.889	0.423		-0.655	0.546	
				WT	1.111	0.309				
		D5	Long	KO	1.111	0.261		-0.185	0.863	
				WT	1.222	0.364				
		D6	Long	KO	1.556	0.580		-0.986	0.387	
				WT	0.667	0.236				
		D7	Long	KO	1.222	0.741		-1.055	0.387	
				WT	0.333	0.167				
		D8	Long	KO	1.000	0.289		-0.140	0.931	
				WT	1.111	0.389				
		D9	Long	KO	1.444	0.603		-0.418	0.730	
				WT	1.000	0.373				
		D10	Long	KO	0.444	0.444		-1.031	0.489	
				WT	0.889	0.564				
		D11	Long	KO	0.778	0.547		-1.122	0.340	
				WT	2.000	0.986				
		D12	Long	KO	1.556	0.689		-0.523	0.666	
				WT	1.000	0.500				
		D13	Long	KO	0.889	0.423		-0.098	0.931	
				WT	0.778	0.364				
		D14	Long	KO	0.333	0.236		-1.366	0.258	
				WT	0.889	0.351				
		D15	Long	KO	1.222	0.521		-0.535	0.666	
				WT	0.667	0.333				
		D16	Long	KO	0.667	0.333		-0.476	0.730	
				WT	0.333	0.167				
Table S5A	Figure 1H,I / Figure S7	D0	Short	KO	0.278	0.121	Mann-Whitney U Test	-1.578	0.222	
				WT	0.056	0.056				
		D1	Short	KO	0.389	0.200		-1.156	0.436	
				WT	0.111	0.111				
		D2	Short	KO	0.722	0.278		-1.031	0.387	
				WT	0.333	0.167				
		D3	Short	KO	1.000	0.527		-1.748	0.161	
				WT	0.222	0.222				
		D4	Short	KO	0.889	0.261		-1.612	0.161	
				WT	0.333	0.167				
		D5	Short	KO	0.444	0.176		-0.051	1.000	
				WT	0.556	0.294				
		D6	Short	KO	1.000	0.333		-1.719	0.136	
				WT	0.333	0.236				
		D7	Short	KO	0.667	0.236		-0.191	0.863	
				WT	1.222	0.596				
		D8	Short	KO	1.000	0.441		-0.095	0.931	
				WT	0.778	0.278				
		D9	Short	KO	1.444	0.556		-0.660	0.546	
				WT	0.778	0.222				
		D10	Short	KO	0.556	0.338		-0.653	0.605	
				WT	0.556	0.176				
		D11	Short	KO	1.000	0.441		-0.146	0.931	
				WT	1.000	0.553				
		D12	Short	KO	0.333	0.167		-0.160	0.931	
				WT	0.444	0.242				
		D13	Short	KO	0.444	0.338		-0.680	0.666	
				WT	0.111	0.111				
		D14	Short	KO	0.556	0.242		-1.068	0.387	
				WT	0.222	0.147				
		D15	Short	KO	0.000	0.000		-2.191	0.113	
				WT	0.667	0.333				
		D16	Short	KO	0.111	0.111		-1.102	0.436	
				WT	0.333	0.167				

HE frequency in 12–13 s window										
Table	Related Figure	Day	Group	Condition	Mean	SEM	Stastistical test	Z	P	*
Table S5	Figure 1H,I / Figure S7	D0	KO	Long	0.278	0.147	Wilcoxon Signed Ranks Test	0.000	1.000	
				Short	0.278	0.121				
		D1	KO	Long	0.056	0.056		-1.604	0.109	
				Short	0.389	0.200				
		D2	KO	Long	0.333	0.236		-1.236	0.216	
				Short	0.722	0.278				
		D3	KO	Long	0.556	0.338		-0.736	0.461	
				Short	1.000	0.527				
		D4	KO	Long	0.889	0.423		0.000	1.000	
				Short	0.889	0.261				
		D5	KO	Long	1.111	0.261		-1.897	0.058	
				Short	0.444	0.176				
		D6	KO	Long	1.556	0.580		-1.406	0.160	
				Short	1.000	0.333				
		D7	KO	Long	1.222	0.741		-0.535	0.593	
				Short	0.667	0.236				
		D8	KO	Long	1.000	0.289		0.000	1.000	
				Short	1.000	0.441				
		D9	KO	Long	1.444	0.603		0.000	1.000	
				Long	1.444	0.556				
		D10	KO	Short	0.444	0.444		-0.577	0.564	
				Long	0.556	0.338				
		D11	KO	Short	0.778	0.547		-0.368	0.713	
				Long	1.000	0.441				
		D12	KO	Short	1.556	0.689		-1.625	0.104	
				Long	0.333	0.167				
		D13	KO	Short	0.889	0.423		-0.816	0.414	
				Long	0.444	0.338				
		D14	KO	Short	0.333	0.236		-1.000	0.317	
				Long	0.556	0.242				
		D15	KO	Short	1.222	0.521		-1.841	0.066	
				Long	0.000	0.000				
		D16	KO	Short	0.667	0.333		-1.512	0.131	
				Long	0.111	0.111				
Table S5	Figure 1H,I / Figure S7	D0	WT	Long	0.111	0.111	Wilcoxon Signed Ranks Test	-0.447	0.655	
				Short	0.056	0.056				
		D1	WT	Long	0.111	0.073		0.000	1.000	
				Short	0.111	0.111				
		D2	WT	Long	0.000	0.000		-1.732	0.083	
				Short	0.333	0.167				
		D3	WT	Long	1.000	0.333		-1.725	0.084	
				Short	0.222	0.222				
		D4	WT	Long	1.111	0.309		-1.823	0.068	
				Short	0.333	0.167				
		D5	WT	Long	1.222	0.364		-1.730	0.084	
				Short	0.556	0.294				
		D6	WT	Long	0.667	0.236		-1.134	0.257	
				Short	0.333	0.236				
		D7	WT	Long	0.333	0.167		-1.633	0.102	
				Short	1.222	0.596				
		D8	WT	Long	1.111	0.389		-1.000	0.317	
				Short	0.778	0.278				
		D9	WT	Long	1.000	0.373		-0.632	0.527	
				Long	0.778	0.222				
		D10	WT	Short	0.889	0.564		-0.378	0.705	
				Long	0.556	0.176				
		D11	WT	Short	2.000	0.986		-1.461	0.144	
				Long	1.000	0.553				
		D12	WT	Short	1.000	0.500		-1.518	0.129	
				Long	0.444	0.242				
		D13	WT	Short	0.778	0.364		-1.857	0.063	
				Long	0.111	0.111				
		D14	WT	Short	0.889	0.351		-1.511	0.131	
				Long	0.222	0.147				
		D15	WT	Short	0.667	0.333		-0.137	0.891	
				Long	0.667	0.333				
		D16	WT	Short	0.333	0.167		0.000	1.000	
				Long	0.333	0.167				

5_14

HE frequency in 13–14 s window										
Table	Related Figure	Day	Condition	Group	Mean	SEM	Stastistical test	Z	P	*
Table S5	Figure 1H,I / Figure S7	D0	Long	KO	0.000	0.000	Mann-Whitney U Test	-1.000	0.730	
				WT	0.056	0.056				
		D1	Long	KO	0.167	0.118		-0.544	0.730	
				WT	0.111	0.111				
		D2	Long	KO	0.389	0.232		-0.503	0.666	
				WT	0.556	0.256				
		D3	Long	KO	0.333	0.236		0.000	1.000	
				WT	0.333	0.236				
		D4	Long	KO	1.000	0.333		-0.095	0.931	
				WT	0.889	0.261				
		D5	Long	KO	0.444	0.242		-1.219	0.297	
				WT	1.667	0.726				
		D6	Long	KO	1.111	0.351		-1.046	0.340	
				WT	0.778	0.465				
		D7	Long	KO	0.556	0.242		-1.068	0.387	
				WT	0.222	0.147				
		D8	Long	KO	1.222	0.662		-0.190	0.863	
				WT	1.000	0.408				
		D9	Long	KO	0.889	0.512		-0.158	0.931	
				WT	1.111	0.655				
		D10	Long	KO	1.222	0.662		-0.252	0.863	
				WT	0.889	0.455				
		D11	Long	KO	1.000	0.373		-0.512	0.666	
				WT	1.444	0.530				
		D12	Long	KO	0.556	0.556		-0.910	0.546	
				WT	0.556	0.338				
		D13	Long	KO	0.667	0.441		-0.527	0.666	
				WT	0.667	0.289				
		D14	Long	KO	0.444	0.338		-0.680	0.666	
				WT	0.111	0.111				
		D15	Long	KO	0.556	0.338		-0.620	0.666	
				WT	0.222	0.147				
		D16	Long	KO	0.778	0.324		-0.591	0.605	
				WT	0.444	0.176				
Table S5A	Figure 1H,I / Figure S7	D0	Short	KO	0.333	0.118	Mann-Whitney U Test	-1.222	0.297	
				WT	0.167	0.118				
		D1	Short	KO	0.389	0.232		-0.252	0.863	
				WT	0.333	0.144				
		D2	Short	KO	0.389	0.162		-0.710	0.546	
				WT	0.222	0.121				
		D3	Short	KO	1.111	0.484		-0.094	0.931	
				WT	0.889	0.261				
		D4	Short	KO	0.667	0.289		-0.145	0.931	
				WT	0.667	0.236				
		D5	Short	KO	0.667	0.236		-0.520	0.666	
				WT	1.111	0.455				
		D6	Short	KO	1.444	0.444		-1.051	0.340	
				WT	0.778	0.222				
		D7	Short	KO	1.333	0.624		-0.486	0.666	
				WT	0.556	0.176				
		D8	Short	KO	0.333	0.236		-1.318	0.258	
				WT	0.778	0.278				
		D9	Short	KO	0.889	0.309		-0.050	1.000	
				WT	0.778	0.222				
		D10	Short	KO	0.889	0.261		-0.622	0.605	
				WT	0.667	0.236				
		D11	Short	KO	1.111	0.351		-0.561	0.605	
				WT	0.889	0.389				
		D12	Short	KO	0.333	0.167		-2.153	0.050	
				WT	1.111	0.261				
		D13	Short	KO	0.778	0.547		-0.505	0.730	
				WT	0.333	0.236				
		D14	Short	KO	0.111	0.111		-2.366	0.040	*
				WT	0.778	0.222				
		D15	Short	KO	0.333	0.236		0.000	1.000	
				WT	0.333	0.236				
		D16	Short	KO	0.333	0.236		-0.449	0.730	
				WT	0.444	0.242				

HE frequency in 13–14 s window										
Table	Related Figure	Day	Group	Condition	Mean	SEM	Stastistical test	Z	P	*
Table S5	Figure 1H,I / Figure S7	D0	KO	Long	0.000	0.000	Wilcoxon Signed Ranks Test	-2.121	0.034	*
				Short	0.333	0.118				
		D1	KO	Long	0.167	0.118		-0.680	0.496	
				Short	0.389	0.232				
		D2	KO	Long	0.389	0.232		0.000	1.000	
				Short	0.389	0.162				
		D3	KO	Long	0.333	0.236		-1.361	0.174	
				Short	1.111	0.484				
		D4	KO	Long	1.000	0.333		-0.647	0.518	
				Short	0.667	0.289				
		D5	KO	Long	0.444	0.242		-0.707	0.480	
				Short	0.667	0.236				
		D6	KO	Long	1.111	0.351		-0.647	0.518	
				Short	1.444	0.444				
		D7	KO	Long	0.556	0.242		-0.689	0.491	
				Short	1.333	0.624				
		D8	KO	Long	1.222	0.662		-1.225	0.221	
				Short	0.333	0.236				
		D9	KO	Long	0.889	0.512		-0.106	0.916	
				Long	0.889	0.309				
		D10	KO	Short	1.222	0.662		-0.333	0.739	
				Long	0.889	0.261				
		D11	KO	Short	1.000	0.373		-0.276	0.783	
				Long	1.111	0.351				
		D12	KO	Short	0.556	0.556		-0.378	0.705	
				Long	0.333	0.167				
		D13	KO	Short	0.667	0.441		0.000	1.000	
				Long	0.778	0.547				
		D14	KO	Short	0.444	0.338		-0.816	0.414	
				Long	0.111	0.111				
		D15	KO	Short	0.556	0.338		-0.412	0.680	
				Long	0.333	0.236				
		D16	KO	Short	0.778	0.324		-0.966	0.334	
				Long	0.333	0.236				
Table S5	Figure 1H,I / Figure S7	D0	WT	Long	0.056	0.056	Wilcoxon Signed Ranks Test	-0.816	0.414	
				Short	0.167	0.118				
		D1	WT	Long	0.111	0.111		-1.633	0.102	
				Short	0.333	0.144				
		D2	WT	Long	0.556	0.256		-1.289	0.197	
				Short	0.222	0.121				
		D3	WT	Long	0.333	0.236		-1.406	0.160	
				Short	0.889	0.261				
		D4	WT	Long	0.889	0.261		-0.707	0.480	
				Short	0.667	0.236				
		D5	WT	Long	1.667	0.726		-0.284	0.777	
				Short	1.111	0.455				
		D6	WT	Long	0.778	0.465		-0.431	0.666	
				Short	0.778	0.222				
		D7	WT	Long	0.222	0.147		-1.342	0.180	
				Short	0.556	0.176				
		D8	WT	Long	1.000	0.408		-0.425	0.671	
				Short	0.778	0.278				
		D9	WT	Long	1.111	0.655		-0.258	0.796	
				Long	0.778	0.222				
		D10	WT	Short	0.889	0.455		-0.412	0.680	
				Long	0.667	0.236				
		D11	WT	Short	1.444	0.530		-2.236	0.025	
				Long	0.889	0.389				
		D12	WT	Short	0.556	0.338		-1.207	0.227	
				Long	1.111	0.261				
		D13	WT	Short	0.667	0.289		-0.966	0.334	
				Long	0.333	0.236				
		D14	WT	Short	0.111	0.111		-1.897	0.058	
				Long	0.778	0.222				
		D15	WT	Short	0.222	0.147		-0.378	0.705	
				Long	0.333	0.236				
		D16	WT	Short	0.444	0.176		0.000	1.000	
				Long	0.444	0.242				

6_1

CR								
Table	Related Figure	Condition	Mean	SEM	Stastistical test	Z	P	*
Table S6	Figure 2B	Long	3316.374	116.818	Wilcoxon Signed Ranks Test	-4.107	< 0.0001	***
		Short	2575.683	17.594				

6_2

RT								
Table	Related Figure	Condition	Mean	SEM	Stastistical test	Z	P	*
Table S6	Figure 2B	Long	99.542	0.146	Wilcoxon Signed Ranks Test	-5.511	< 0.00000001	***
		Short	97.333	0.536				

6_3

Correlation between Long and Short in RT								
Table	Related Figure	Condition	Mean	SEM	Stastistical test	Spearman’s ρ	P	*
Table S6	Figure 2B	Long	99.542	0.146	Spearman's rank correlation coefficient	0.601	< 0.0001	***
		Short	97.333	0.536				

Supplementary Table 6. CR and RT. Differences between conditions were analyzed using the Wilcoxon signed-rank test. Correlation analysis for RT was conducted using Spearman’s rank correlation coefficient. ***$p < 0.001$ ($N = 40$). Abbreviations: CR, correct rate; RT, reaction time.

7_1

Table	Related Figure	Condition	Mean	SEM	Stastistical test	Spearman's ρ	Bonferroni corrected P	*
Human: Correlation between LATENCY (L-S difference in RT) and ACCURACY (CR)								
Table S7	Figure 3C	RT difference	740.69	110.733	Spearman's rank correlation coefficient	0.094	1.000	
		CR for Long	99.542	0.146				
		RT difference	740.69	110.733		0.265	0.198	
		CR for Short	97.333	0.536				

Table	Related Figure	Condition	Mean	SEM	Stastistical test	Spearman's ρ	Bonferroni corrected P	*
Human: Correlation between LATENCY (RT) and ACCURACY (CR)								
Table S7	Figure S8A	RT for Long	3316.37	116.82	Spearman's rank correlation coefficient	0.032	1.0000	
		CR for Long	99.542	0.146				
		RT for Short	2575.68	17.59		0.275	1.0000	
		CR for Short	97.333	0.536				

Supplementary Table 7. Correlation between RT/Latency and CR/Accuracy. Correlation analyses in human and mouse data were conducted separately using Spearman's rank correlation coefficient. *p < 0.05, **p < 0.01 (N = 40 for humans; N = 9 for mice). Abbreviations: CR, correct rate; RT, reaction time; HE, head entry; D, training day; KO, CRBN KO mice; WT, wild-type mice.

7_2

Mouse: Correlation between LATENCY (L-S difference in HE peak latency) and ACCURACY (hit and correct rejection rates) in KO mice

Table	Related Figure	Day	Condition	Pair	Mean	SEM	Statistical test	Spearman's ρ	P	*
Table S7	Figure 3C / Figure S8C	D0	Long	LATENCY	0.333	2.021	Spearman's rank correlation coefficient	-0.157	1.000	
				ACCURACY	27.780	8.780				
		D1	Long	LATENCY	-2.333	1.213		-0.095	1.000	
				ACCURACY	18.890	7.720				
		D2	Long	LATENCY	-2.000	2.055		0.613	0.159	
				ACCURACY	32.220	9.830				
		D3	Long	LATENCY	-2.000	1.944		0.047	1.000	
				ACCURACY	38.890	10.060				
		D4	Long	LATENCY	-0.556	2.199		0.551	0.248	
				ACCURACY	56.670	12.020				
		D5	Long	LATENCY	2.000	1.756		0.494	0.354	
				ACCURACY	50.000	9.130				
		D6	Long	LATENCY	1.444	1.556		0.302	0.859	
				ACCURACY	75.560	7.660				
		D7	Long	LATENCY	0.778	1.942		-0.017	1.000	
				ACCURACY	68.890	11.480				
		D8	Long	LATENCY	-0.111	1.867		0.433	0.489	
				ACCURACY	87.780	7.950				
		D9	Long	LATENCY	2.444	0.852		-0.337	0.751	
				ACCURACY	87.780	8.620				
		D10	Long	LATENCY	2.889	0.964		-0.345	0.726	
				ACCURACY	80.000	10.410				
		D11	Long	LATENCY	1.556	1.355		-0.309	0.837	
				ACCURACY	70.000	11.550				
		D12	Long	LATENCY	1.000	1.014		0.334	0.759	
				ACCURACY	83.330	9.130				
		D13	Long	LATENCY	2.556	0.899		-0.397	0.581	
				ACCURACY	77.780	7.030				
		D14	Long	LATENCY	2.222	0.778		-0.196	1.000	
				ACCURACY	83.330	7.640				
		D15	Long	LATENCY	3.889	1.328		-0.672	0.095	
				ACCURACY	82.220	9.250				
		D16	Long	LATENCY	3.556	1.345		-0.808	0.017	*
				ACCURACY	87.780	4.940				
Table S7	Figure 3C / Figure S8C	D0	Short	LATENCY	0.333	2.021	Spearman's rank correlation coefficient	-0.568	0.222	
				ACCURACY	95.560	2.420				
		D1	Short	LATENCY	-2.333	1.213		0.055	1.775	
				ACCURACY	93.330	2.360				
		D2	Short	LATENCY	-2.000	2.055		-0.383	0.618	
				ACCURACY	88.890	2.610				
		D3	Short	LATENCY	-2.000	1.944		-0.183	1.276	
				ACCURACY	88.890	3.510				
		D4	Short	LATENCY	-0.556	2.199		-0.722	0.056	
				ACCURACY	78.890	6.110				
		D5	Short	LATENCY	2.000	1.756		-0.735	0.048	*
				ACCURACY	84.440	3.380				
		D6	Short	LATENCY	1.444	1.556		-0.690	0.080	
				ACCURACY	81.110	4.230				
		D7	Short	LATENCY	0.778	1.942		-0.337	0.752	
				ACCURACY	70.000	7.070				
		D8	Short	LATENCY	-0.111	1.867		0.139	1.442	
				ACCURACY	77.780	5.470				
		D9	Short	LATENCY	2.444	0.852		0.056	1.773	
				ACCURACY	71.110	4.230				
		D10	Short	LATENCY	2.889	0.964		0.407	0.553	
				ACCURACY	73.330	5.000				
		D11	Short	LATENCY	1.556	1.355		0.470	0.403	
				ACCURACY	76.670	7.640				
		D12	Short	LATENCY	1.000	1.014		0.000	2.000	
				ACCURACY	71.110	6.550				
		D13	Short	LATENCY	2.556	0.899		0.291	0.894	
				ACCURACY	73.330	8.160				
		D14	Short	LATENCY	2.222	0.778		0.419	0.523	
				ACCURACY	63.330	7.990				
		D15	Short	LATENCY	3.889	1.328		0.798	0.020	*
				ACCURACY	67.780	8.460				
		D16	Short	LATENCY	3.556	1.345		0.788	0.023	*
				ACCURACY	70.000	7.260				

Mouse: Correlation between LATENCY (L-S difference in HE peak latency) and ACCURACY (hit and correct rejection rates) in WT mice

Table	Related Figure	Day	Condition	Pair	Mean	SEM	Statistical test	Spearman's ρ	P	*
Table S7	Figure 3C / Figure S8C	D0	Long	LATENCY	-2.222	2.093	Spearman's rank correlation coefficient	0.409	0.550	
				ACCURACY	6.670	2.360				
		D1	Long	LATENCY	-2.333	1.302		-0.431	0.493	
				ACCURACY	13.330	5.270				
		D2	Long	LATENCY	1.222	1.801		-0.389	0.603	
				ACCURACY	24.440	8.520				
		D3	Long	LATENCY	1.556	2.001		0.621	0.149	
				ACCURACY	24.440	7.090				
		D4	Long	LATENCY	-0.222	1.928		0.321	0.800	
				ACCURACY	56.670	10.270				
		D5	Long	LATENCY	2.778	1.778		0.178	1.000	
				ACCURACY	54.440	11.320				
		D6	Long	LATENCY	3.333	1.472		0.127	1.000	
				ACCURACY	62.220	9.090				
		D7	Long	LATENCY	0.111	1.837		0.025	1.000	
				ACCURACY	71.110	6.330				
		D8	Long	LATENCY	-1.222	1.441		-0.070	1.000	
				ACCURACY	81.110	4.840				
		D9	Long	LATENCY	0.111	0.949		-0.083	1.000	
				ACCURACY	78.890	6.330				
		D10	Long	LATENCY	-0.889	1.467		0.191	1.000	
				ACCURACY	83.330	5.770				
		D11	Long	LATENCY	1.667	1.354		-0.569	0.220	
				ACCURACY	84.440	5.560				
		D12	Long	LATENCY	0.556	1.415		-0.411	0.545	
				ACCURACY	86.670	4.710				
		D13	Long	LATENCY	0.667	0.687		0.411	0.545	
				ACCURACY	86.670	4.710				
		D14	Long	LATENCY	0.556	1.314		-0.416	0.532	
				ACCURACY	84.440	3.770				
		D15	Long	LATENCY	1.000	0.577		-0.101	1.000	
				ACCURACY	87.780	4.010				
		D16	Long	LATENCY	3.778	1.051		-0.009	1.000	
				ACCURACY	92.220	3.240				
Table S7	Figure 3C / Figure S8C	D0	Short	LATENCY	-2.222	2.093	Spearman's rank correlation coefficient	0.000	1.000	
				ACCURACY	98.890	1.110				
		D1	Short	LATENCY	-2.333	1.302		0.000	0.000	
				ACCURACY	100.000	0.000				
		D2	Short	LATENCY	1.222	1.801		-0.487	0.368	
				ACCURACY	94.440	1.760				
		D3	Short	LATENCY	1.556	2.001		-0.377	0.634	
				ACCURACY	88.890	2.610				
		D4	Short	LATENCY	-0.222	1.928		-0.272	0.957	
				ACCURACY	77.780	4.340				
		D5	Short	LATENCY	2.778	1.778		0.064	1.000	
				ACCURACY	83.330	2.360				
		D6	Short	LATENCY	3.333	1.472		0.134	1.000	
				ACCURACY	90.000	4.080				
		D7	Short	LATENCY	0.111	1.837		0.550	0.250	
				ACCURACY	86.670	1.670				
		D8	Short	LATENCY	-1.222	1.441		0.438	0.477	
				ACCURACY	78.890	4.550				
		D9	Short	LATENCY	0.111	0.949		-0.305	0.849	
				ACCURACY	86.670	2.890				
		D10	Short	LATENCY	-0.889	1.467		-0.106	1.000	
				ACCURACY	82.220	4.940				
		D11	Short	LATENCY	1.667	1.354		-0.507	0.328	
				ACCURACY	77.780	3.640				
		D12	Short	LATENCY	0.556	1.415		0.026	1.000	
				ACCURACY	78.890	4.840				
		D13	Short	LATENCY	0.667	0.687		-0.301	0.863	
				ACCURACY	85.560	2.940				
		D14	Short	LATENCY	0.556	1.314		0.207	1.000	
				ACCURACY	81.110	4.550				
		D15	Short	LATENCY	1.000	0.577		0.017	1.000	
				ACCURACY	81.110	4.840				
		D16	Short	LATENCY	3.778	1.051		0.681	0.087	
				ACCURACY	77.780	3.640				

7_3

Mouse: Correlation between LATENCY (HE peak latency) and ACCURACY (hit and correct rejection rates) in KO mice

Table	Related Figure	Day	Condition	Pair	Mean	SEM	Stastistical test	Spearman's ρ	P	*
Table S7	Figure S8B	D0	Long	LATENCY	4.000	1.093	Spearman's rank correlation coefficient	-0.035	1.000	
				ACCURACY	27.780	8.780				
		D1	Long	LATENCY	1.000	0.408		0.401	0.568	
				ACCURACY	18.890	7.720				
		D2	Long	LATENCY	4.222	1.310		0.382	0.621	
				ACCURACY	32.220	9.830				
		D3	Long	LATENCY	3.667	1.291		0.096	1.000	
				ACCURACY	38.890	10.060				
		D4	Long	LATENCY	2.889	1.296		0.715	0.061	
				ACCURACY	56.670	12.020				
		D5	Long	LATENCY	4.667	1.202		0.430	0.497	
				ACCURACY	50.000	9.130				
		D6	Long	LATENCY	4.222	1.164		0.342	0.736	
				ACCURACY	75.560	7.660				
		D7	Long	LATENCY	3.556	1.215		0.274	0.952	
				ACCURACY	68.890	11.480				
		D8	Long	LATENCY	3.222	1.382		0.228	1.000	
				ACCURACY	87.780	7.950				
		D9	Long	LATENCY	5.000	1.333		-0.666	0.100	
				ACCURACY	87.780	8.620				
		D10	Long	LATENCY	5.111	1.172		-0.646	0.121	
				ACCURACY	80.000	10.410				
		D11	Long	LATENCY	5.556	1.600		-0.369	0.657	
				ACCURACY	70.000	11.550				
		D12	Long	LATENCY	3.111	1.252		-0.076	1.000	
				ACCURACY	83.330	9.130				
		D13	Long	LATENCY	5.111	1.348		-0.797	0.020	*
				ACCURACY	77.780	7.030				
		D14	Long	LATENCY	3.889	0.889		-0.101	1.000	
				ACCURACY	83.330	7.640				
		D15	Long	LATENCY	5.222	1.579		-0.787	0.024	*
				ACCURACY	82.220	9.250				
		D16	Long	LATENCY	5.000	1.414		-0.850	0.007	*
				ACCURACY	87.780	4.940				
Table S7	Figure S8B	D0	Short	LATENCY	3.667	1.312	Spearman's rank correlation coefficient	0.661	0.105	
				ACCURACY	95.560	2.420				
		D1	Short	LATENCY	3.333	1.312		-0.283	0.923	
				ACCURACY	93.330	2.360				
		D2	Short	LATENCY	6.222	1.289		0.314	0.821	
				ACCURACY	88.890	2.610				
		D3	Short	LATENCY	5.667	1.607		0.148	1.000	
				ACCURACY	88.890	3.510				
		D4	Short	LATENCY	3.444	1.271		0.570	0.218	
				ACCURACY	78.890	6.110				
		D5	Short	LATENCY	2.667	0.645		0.679	0.089	
				ACCURACY	84.440	3.380				
		D6	Short	LATENCY	2.778	0.741		0.846	0.008	**
				ACCURACY	81.110	4.230				
		D7	Short	LATENCY	2.778	1.392		0.036	1.000	
				ACCURACY	70.000	7.070				
		D8	Short	LATENCY	3.333	1.067		0.563	0.228	
				ACCURACY	77.780	5.470				
		D9	Short	LATENCY	2.556	0.801		0.223	1.000	
				ACCURACY	71.110	4.230				
		D10	Short	LATENCY	2.222	0.683		0.436	0.481	
				ACCURACY	73.330	5.000				
		D11	Short	LATENCY	4.000	1.190		0.299	0.868	
				ACCURACY	76.670	7.640				
		D12	Short	LATENCY	2.111	0.772		0.711	0.064	
				ACCURACY	71.110	6.550				
		D13	Short	LATENCY	2.556	0.899		0.757	0.037	*
				ACCURACY	73.330	8.160				
		D14	Short	LATENCY	1.667	0.408		0.745	0.043	*
				ACCURACY	63.330	7.990				
		D15	Short	LATENCY	1.333	0.577		0.589	0.190	
				ACCURACY	67.780	8.460				
		D16	Short	LATENCY	1.444	0.377		0.565	0.225	
				ACCURACY	70.000	7.260				

Mouse: Correlation between LATENCY (HE peak latency) and ACCURACY (hit and correct rejection rates) in WT mice

Table	Related Figure	Day	Condition	Pair	Mean	SEM	Stastistical test	Spearman's ρ	P	*
Table S7	Figure S8B	D0	Long	LATENCY	1.778	1.222	Spearman's rank correlation coefficient	0.650	0.116	
				ACCURACY	6.670	2.360				
		D1	Long	LATENCY	2.000	1.443		-0.042	1.000	
				ACCURACY	13.330	5.270				
		D2	Long	LATENCY	3.111	1.438		0.122	1.000	
				ACCURACY	24.440	8.520				
		D3	Long	LATENCY	5.111	1.728		0.205	1.000	
				ACCURACY	24.440	7.090				
		D4	Long	LATENCY	2.111	1.111		0.405	0.559	
				ACCURACY	56.670	10.270				
		D5	Long	LATENCY	5.444	1.608		-0.154	1.000	
				ACCURACY	54.440	11.320				
		D6	Long	LATENCY	5.111	1.476		-0.399	0.574	
				ACCURACY	62.220	9.090				
		D7	Long	LATENCY	4.222	0.572		-0.125	1.000	
				ACCURACY	71.110	6.330				
		D8	Long	LATENCY	3.222	1.278		-0.396	0.582	
				ACCURACY	81.110	4.840				
		D9	Long	LATENCY	4.000	1.179		0.022	1.000	
				ACCURACY	78.890	6.330				
		D10	Long	LATENCY	3.111	1.060		-0.502	0.337	
				ACCURACY	83.330	5.770				
		D11	Long	LATENCY	5.556	1.365		-0.618	0.152	
				ACCURACY	84.440	5.560				
		D12	Long	LATENCY	3.778	1.245		-0.614	0.157	
				ACCURACY	86.670	4.710				
		D13	Long	LATENCY	2.889	0.633		-0.130	1.000	
				ACCURACY	86.670	4.710				
		D14	Long	LATENCY	4.333	0.833		0.204	1.000	
				ACCURACY	84.440	3.770				
		D15	Long	LATENCY	4.333	0.667		-0.351	0.708	
				ACCURACY	87.780	4.010				
		D16	Long	LATENCY	5.778	1.103		-0.069	1.000	
				ACCURACY	92.220	3.240				
Table S7	Figure S8B	D0	Short	LATENCY	4.000	1.616	Spearman's rank correlation coefficient	0.000	1.000	
				ACCURACY	98.890	1.110				
		D1	Short	LATENCY	4.333	1.258		0.000	0.000	
				ACCURACY	100.000	0.000				
		D2	Short	LATENCY	1.889	1.020		0.095	1.000	
				ACCURACY	94.440	1.760				
		D3	Short	LATENCY	3.556	1.692		0.279	0.934	
				ACCURACY	88.890	2.610				
		D4	Short	LATENCY	2.333	1.202		0.018	1.000	
				ACCURACY	77.780	4.340				
		D5	Short	LATENCY	2.667	0.816		0.587	0.192	
				ACCURACY	83.330	2.360				
		D6	Short	LATENCY	1.778	0.969		0.412	0.541	
				ACCURACY	90.000	4.080				
		D7	Short	LATENCY	4.111	1.567		-0.334	0.760	
				ACCURACY	86.670	1.670				
		D8	Short	LATENCY	4.444	1.271		-0.388	0.604	
				ACCURACY	78.890	4.550				
		D9	Short	LATENCY	3.889	0.484		0.076	1.000	
				ACCURACY	86.670	2.890				
		D10	Short	LATENCY	4.000	1.404		0.561	0.232	
				ACCURACY	82.220	4.940				
		D11	Short	LATENCY	3.889	1.047		0.513	0.317	
				ACCURACY	77.780	3.640				
		D12	Short	LATENCY	3.222	0.862		0.296	0.880	
				ACCURACY	78.890	4.840				
		D13	Short	LATENCY	2.222	0.521		0.701	0.071	
				ACCURACY	85.560	2.940				
		D14	Short	LATENCY	3.778	1.064		-0.190	1.000	
				ACCURACY	81.110	4.550				
		D15	Short	LATENCY	3.333	0.726		0.332	0.766	
				ACCURACY	81.110	4.840				
		D16	Short	LATENCY	2.000	0.471		0.265	0.982	
				ACCURACY	77.780	3.640				
