# Supplementary material for: Cereblon-related mild intellectual disability disrupts response inhibition and uniformity of group–individual strategies
Source: Front Neurosci. 2026 Jul 13;20:1782687. doi: 10.3389/fnins.2026.1782687 (PMC13402522; doi:10.3389/fnins.2026.1782687)
Supplement: Supplementary file 2 [file Data_sheet_2.pdf]

Supplementary figures

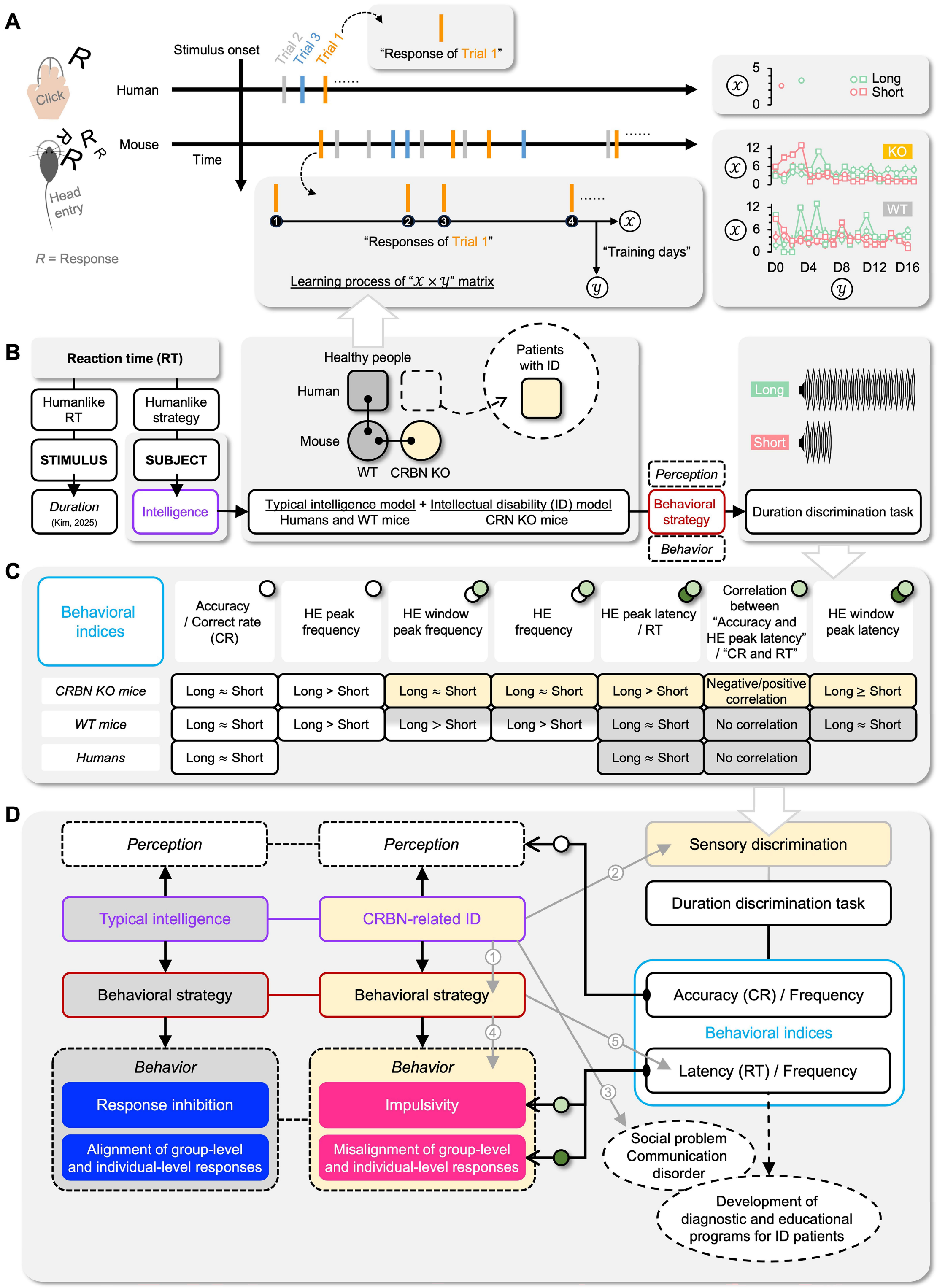

Supplementary Figure 1. Categorization of behavioral indices in the duration discrimination task. (A) Human participants completed multiple trials within a single experimental day (x-axis). In contrast, mice underwent operant chamber conditioning over 16 days (x–y axis). Humans typically make a single response per trial, whereas mice exhibit repeated responses. Humans may have relied on prior knowledge to develop behavioral strategies, whereas mice likely developed their strategies gradually through daily task training. (B) Based on a previous study demonstrating human-like reaction times (RT) in mice in relation to stimulus duration (1), we designed a duration discrimination task that incorporated the distinct behavioral characteristics of each species. We focused on human-like behavioral strategies in mice, as reflected in RT patterns associated with intelligence. Through these models, we expected to predict the behavioral characteristics of patients with ID. RTs to 10-s and 2-s conditions may reveal differences in response patterns across species. RT has been associated with several behavioral processes, including impulsivity (2), response inhibition (3), and behavioral strategies involved in decision-making (4). These RT-based behavioral indices may be useful for characterizing heterogeneous behavioral phenotypes associated with intellectual disability. (C) In this study, behavioral indices differed across groups. CRBN KO mice, a model of intellectual disability (ID), differed from WT mice and humans, who both represent typical intelligence. Behavioral indices include accuracy, frequency, and latency in mice, and CR and RT in humans. Boxes of the same color indicate significant results with similar response patterns. White boxes indicate behavioral characteristics related to perception that are common across all subjects regardless of ID. Yellow indicates characteristics specific to KO mice with ID, and gray indicates those specific to WT mice and humans without ID. White, green, and dark green circles correspond to the individual behavioral indices shown in panel D. During the duration discrimination task, differences between groups and conditions were observed. (D) Differences in intelligence between subjects affects cognitive strategy (①) (5) and sensory discrimination (②) (6), and are associated with social problems and communication disorders (③) (7). Cognitive strategy affects behavioral responses (④) (8, 9) such as RT (⑤) (10–12), but not perception. This schematic model is presented as a conceptual framework integrating the present findings with previous literature and should not be interpreted as evidence of causal relationships directly demonstrated in the current study. Our data support classifying behavioral indices into two domains: “perception” and “behavior.” The former included accuracy (CR) and certain frequency measures, whereas the latter included latency (RT) and other frequency measures. In our data, behavioral indices—particularly HE window peak latency and HE frequency in WT mice—explained both perceptual processes (similar to KO mice) and behavioral processes (different from KO mice). While ID affected behavioral responses, perceptual ability remained intact during task performance. Characteristics specific to each subject, such as intelligence and behavioral strategy, led to consistent pattern of response across various behavioral indices. Behavioral indices differentiated two properties: “response inhibition versus impulsivity” and “alignment versus misalignment between group- and individual-level responses.”

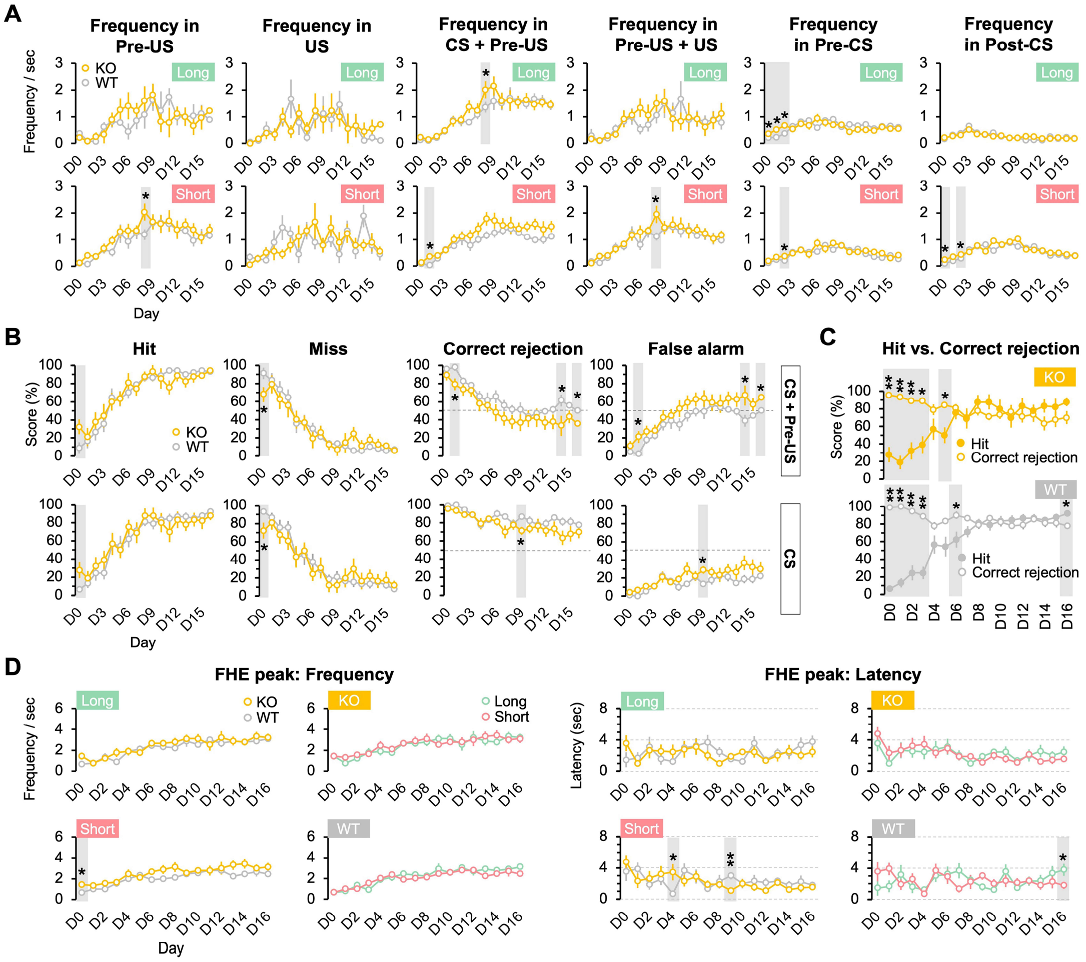

Supplementary Figure 2. HE frequency and accuracy. (A) HE frequencies across different time windows. (B) Accuracy rate within the "CS + Pre-US" and "CS" time windows. (C) Difference between hit and correction rates. (D) Fastest HE (FHE) peak frequency and latency were comparable across groups and conditions. See Table S1–S3 for statistical details. Wilcoxon signed-ranks test and Mann–Whitney U test, *, $p < 0.05$; **, $p < 0.01$. Abbreviations: D, day; CS, conditioned stimulus; US, unconditioned stimulus; Long, 10-s condition; Short, 2-s condition; WT, wild-type mice; KO, CRBN KO mice; HE, head entry; FHE, fastest HE.

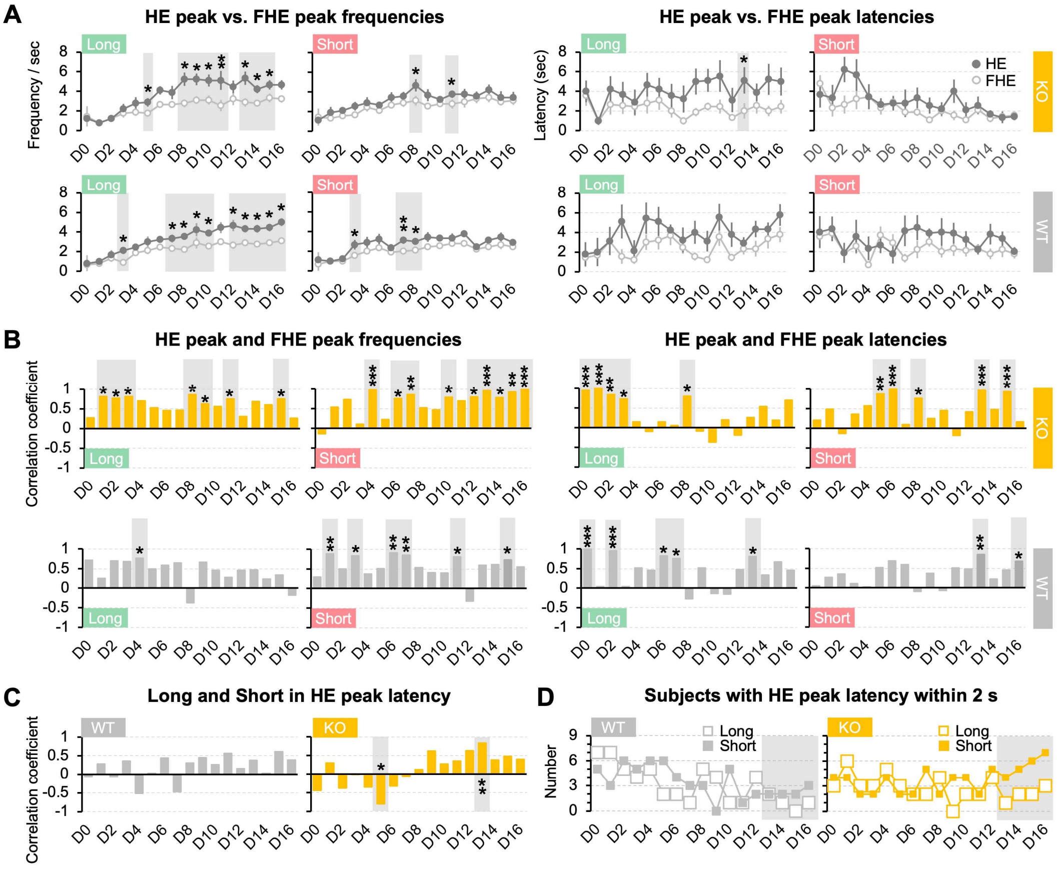

Supplementary Figure 3. HE and FHE peaks. (A) Difference between the HE and FHE peaks were more prominent for frequency in both groups. (B) Correlation between the HE and FHE peaks in the late training days was more prominent in KO mice. (C) Correlations between HE peak latencies for 10-s and 2-s conditions were not significant, except on D5 and D13 in KO mice. (D) The number of KO mice exhibiting HE peak latencies within 2 s increased linearly, from four subjects on D13 to seven subjects on D16. See Table S3 for statistical details. Wilcoxon signed-ranks test, Mann–Whitney U test, and Spearman correlation test. *, $p < 0.05$; **, $p < 0.01$; ***, $p < 0.001$. Error bars denote 95% confidence intervals. Abbreviations: D, day; CS, conditioned stimulus; US, unconditioned stimulus; Long, 10-s condition; Short, 2-s condition; WT, wild-type mice; KO, CRBN KO mice; HE, head entry; FHE, fastest HE.

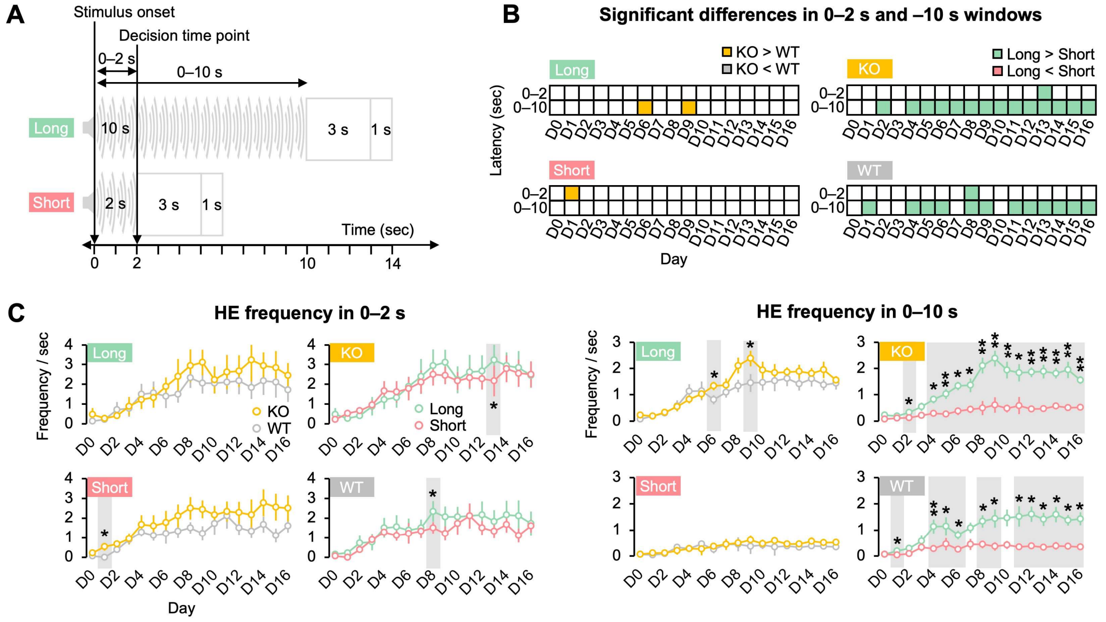

Supplementary Figure 4. HE frequencies in 0–2 and 0–14 after the stimulus onset. (A) Analysis time windows were segmented into 0–2 s, 0–10 s, and successive 1-s bins (0–14 s). (B) Group and condition differences rarely occurred within the 0–2 s window. In the 0–10 s window, both groups showed clear difference between 10-s and 2-s conditions. Significant differences are indicated by green- and orange-colored boxes. (C) The green- and orange-colored boxes in panel B were derived from the plots presented in panel C. See Table S4 for statistical details. Wilcoxon signed-ranks test and Mann–Whitney U test. *, $p < 0.05$; **, $p < 0.01$. Error bars denote 95% confidence intervals. Abbreviations: D, day; CS, conditioned stimulus; US, unconditioned stimulus; Long, 10-s condition; Short, 2-s condition; WT, wild-type mice; KO, CRBN KO mice; HE, head entry.

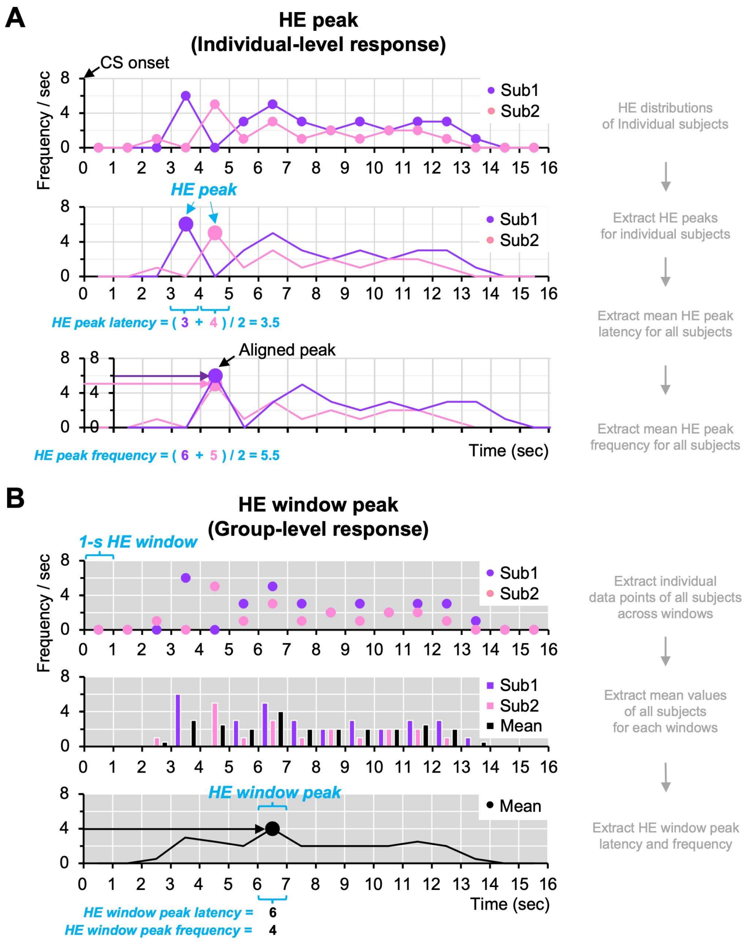

Supplementary Figure 5. Extraction of HE peak and HE window peak. The HE peak latency may not be consistent with the HE window peak latency. Abbreviations: CS, conditioned stimulus; HE, head entry; Sub, subject.

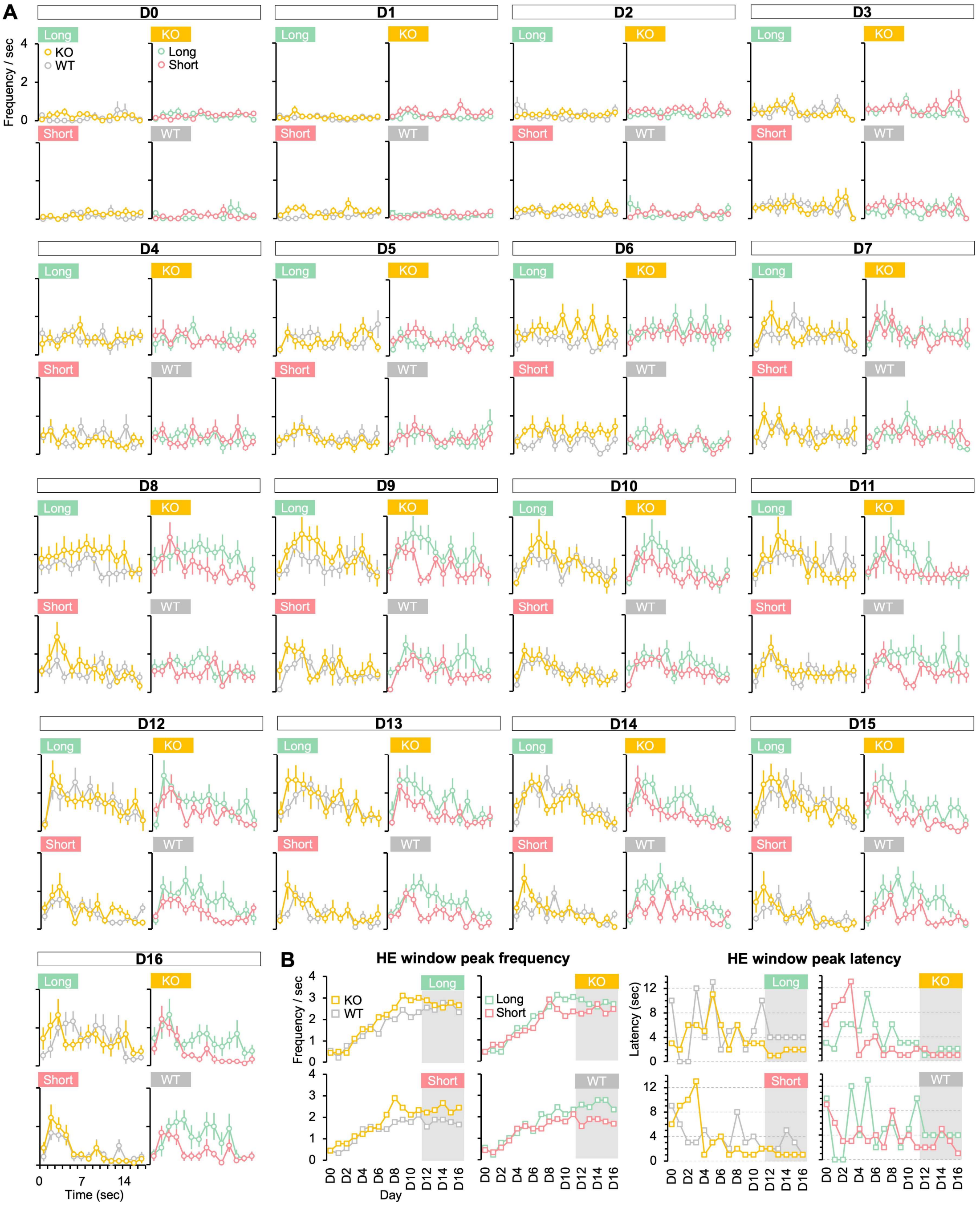

Supplementary Figure 6. Daily HE frequency change across 1-s latency bins and HE window peak. (A) Distribution of HE frequency per 1-s bin was presented for individual training days. The peak of frequency on each day was defined as the HE window peak. (B) During D12–D16, when HE frequency had reached a plateau, the HE window peak frequency was lower in the 2-s condition than in the 10-s condition in WT mice, a pattern that differed from that observed in KO mice. Regarding HE window peak latency, WT

mice exhibited relatively slower in 10-s condition, whereas KO mice consistently responded rapidly in 2-s condition. The HE window peak occurred within 2 s in KO mice, whereas it remained consistently between 4 and 5 s in the 10-s condition and fluctuated between 2 and 6 s in the 2-s condition in WT mice. Error bars denote 95% confidence intervals. See also Figure 1H. Abbreviations: D, day; CS, conditioned stimulus; US, unconditioned stimulus; Long, 10-s condition; Short, 2-s condition; WT, wild-type mice; KO, KO mice; HE, head entry.

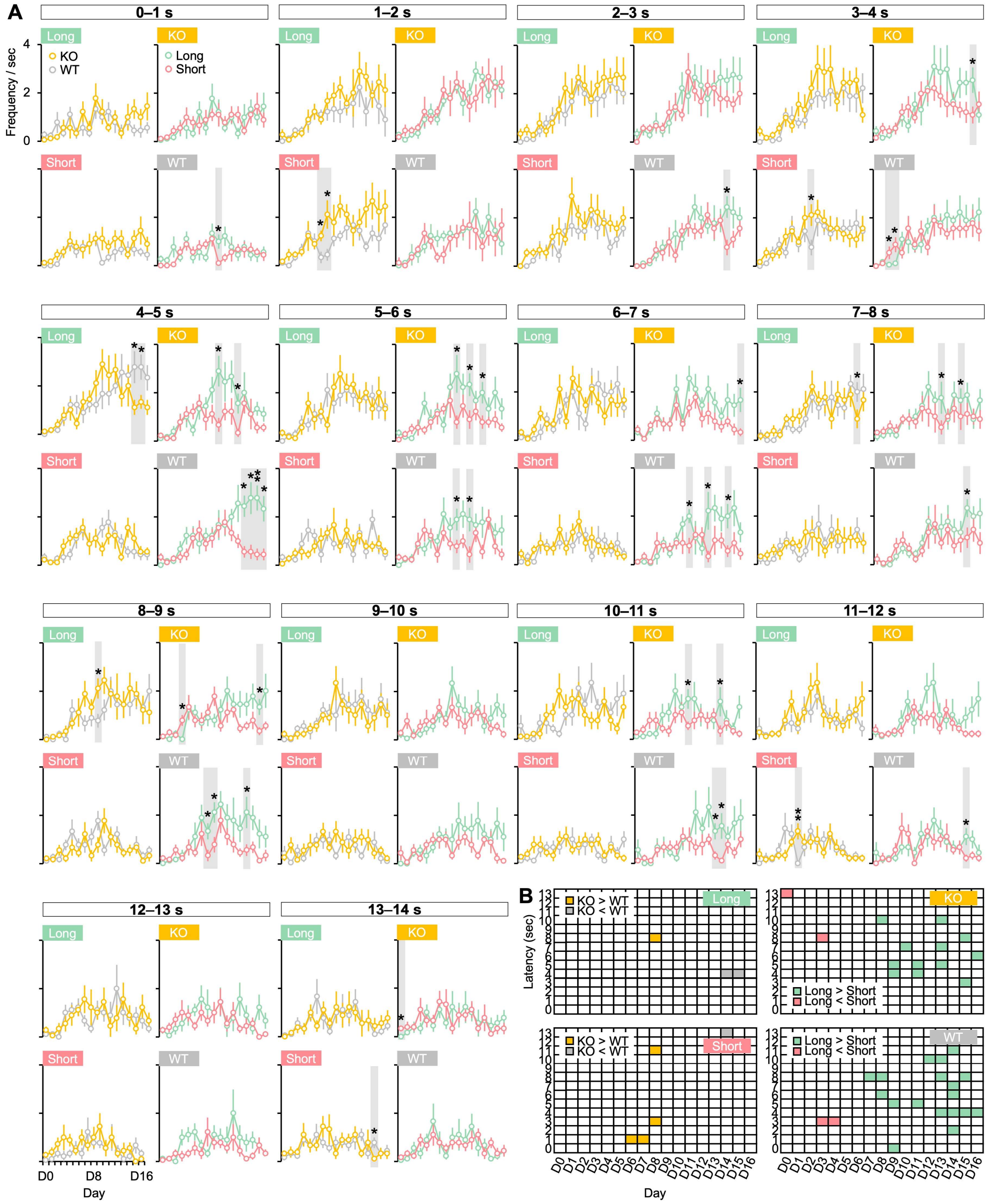

Supplementary Figure 7. Difference between groups and conditions for HE frequencies per 1-s latency bins across training days. The green- and orange-colored boxes in panel B were derived from the plots presented in panel A. See also Figure 1I and Table S5 for full statistics. Wilcoxon signed-ranks test and Mann–Whitney U test, *, $p < 0.05$. Error bars denote 95% confidence intervals. Abbreviations: D, day; CS, conditioned

stimulus; US, unconditioned stimulus; Long, 10-s condition; Short, 2-s condition; WT, wild-type mice; KO, CRBN KO mice.

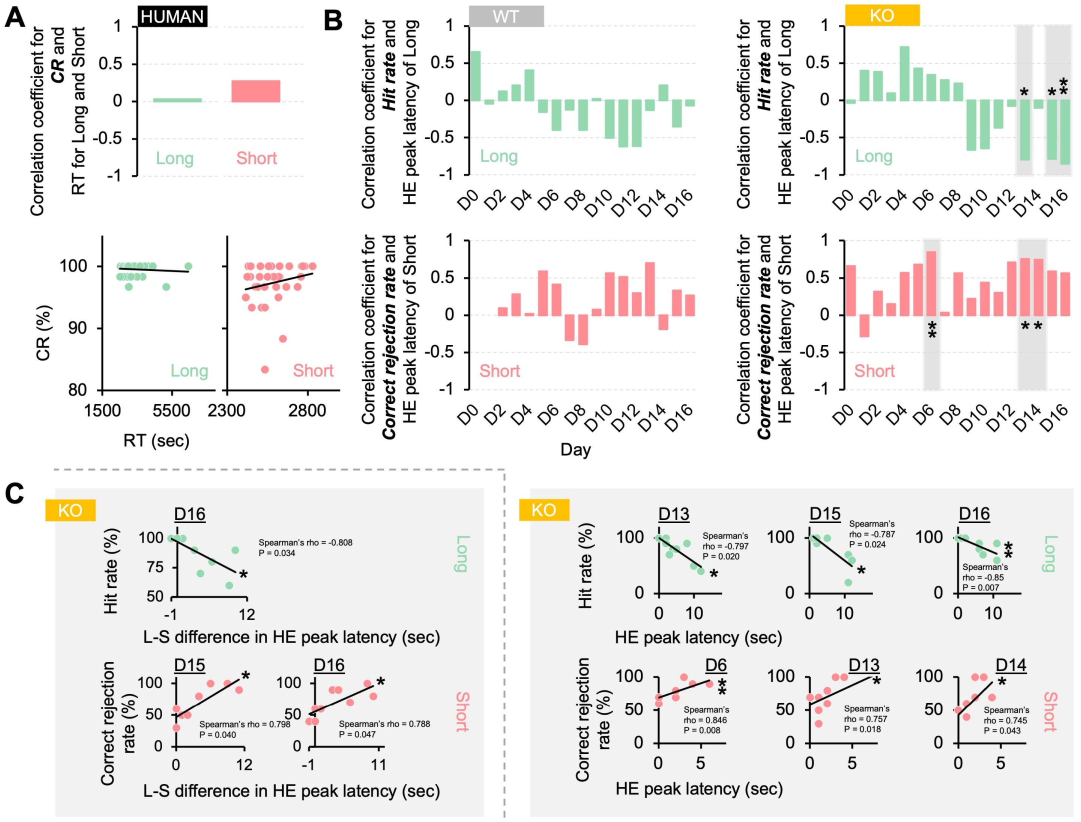

Supplementary Figure 8. Correlation between RT (latency) and CR (accuracy). (A–B) Correlation between RT (HE peak latency) and CR (hit and correct rejection rates). (C) Correlation between the L–S difference in HE peak latency and accuracy (hit and correct rejection rates). See Figure 3C and Table S7 for statistical details. Spearman correlation. *, p < 0.05; **, p < 0.01. Abbreviations: D, day; CS, conditioned stimulus; US, unconditioned stimulus; Long, 10-s condition; Short, 2-s condition; L–S, Long–Short; WT, wild-type mice; KO, CRBN KO mice; HE, head entry.

References

1. C. H. Kim, Harmonization of the fastest and densest responses reflects humanlike reaction time in mice. *Front Neurosci* **19**, 1501374 (2025).
2. de Wit, H. (2009). Impulsivity as a determinant and consequence of drug use: a review of underlying processes. Addict Biol *14*, 22-31. 10.1111/j.1369-1600.2008.00129.x.
3. Burle, B., Vidal, F., Tandonnet, C., and Hasbroucq, T. (2004). Physiological evide nce for response inhibition in choice reaction time tasks. Brain Cogn *56*, 153-164. 10.1016/j.bandc.2004.06.004.
4. Wierda, T.S., Dora, S., Pennartz, C.M.A., and Mejias, J.F. (2025). Diverse and fle xible behavioral strategies arise in recurrent neural networks trained on multisenso ry decision making. PLoS Comput Biol *21*, e1013559. 10.1371/journal.pcbi.1013 559.
5. J. Glascher *et al.*, Lesion mapping of cognitive abilities linked to intelligence. *Neuron* **61**, 681-691 (2009).
6. M. D. Melnick, B. R. Harrison, S. Park, L. Bennetto, D. Tadin, A strong interactive link between sensory discriminations and intelligence. *Curr Biol* **23**, 1013-1017 (2013).
7. F. Fernandez, C. C. Garner, Over-inhibition: a model for developmental intellectual disability. *Trends Neurosci* **30**, 497-503 (2007).
8. S. E. Whitely, Individual inconsistency: Implications for test reliability and behavioral predictability. *Applied Psychological Measurement* **2**, 571-579 (1978).
9. R. Hertwig, G. Gigerenzer, Behavioral inconsistencies do not imply inconsistent strategies. *Frontiers in Psychology* **2**, 292 (2011).
10. K. C. Chen, C. Y. Weng, S. Hsiao, W. L. Tsao, M. Koo, Cognitive decline and slower reaction time in elderly individuals with mild cognitive impairment. *Psychogeriatrics* **17**, 364-370 (2017).
11. P. De Boeck, M. Jeon, An overview of models for response times and processes in cognitive tests. *Frontiers in psychology* **10**, 102 (2019).
12. W. E. Hockley, Analysis of response time distributions in the study of cognitive processes. *Journal of Experimental Psychology: Learning, Memory, and Cognition* **10**, 598 (1984).
